# Iminosugars: Effects of Stereochemistry, Ring Size, and *N*-Substituents on Glucosidase Activities

**DOI:** 10.3390/ph12030108

**Published:** 2019-07-12

**Authors:** Luís O. B. Zamoner, Valquiria Aragão-Leoneti, Ivone Carvalho

**Affiliations:** School of Pharmaceutical Sciences of Ribeirão Preto, University of São Paulo, Av. do Café s/n, Monte Alegre, CEP14040-903 Ribeirão Preto, Brazil

**Keywords:** iminosugars, polyhydroxypiperidines, polyhydroxyazepanes, glucosidase inhibition, miglustat, miglitol

## Abstract

*N*-substituted iminosugar analogues are potent inhibitors of glucosidases and glycosyltransferases with broad therapeutic applications, such as treatment of diabetes and Gaucher disease, immunosuppressive activities, and antibacterial and antiviral effects against HIV, HPV, hepatitis C, bovine diarrhea (BVDV), Ebola (EBOV) and Marburg viruses (MARV), influenza, Zika, and dengue virus. Based on our previous work on functionalized isomeric 1,5-dideoxy-1,5-imino-D-gulitol (L-*gulo*-piperidines, with inverted configuration at C-2 and C-5 in respect to glucose or deoxynojirimycin (DNJ)) and 1,6-dideoxy-1,6-imino-D-mannitol (D-*manno*-azepane derivatives) cores *N*-linked to different sites of glucopyranose units, we continue our studies on these alternative iminosugars bearing simple *N*-alkyl chains instead of glucose to understand if these easily accessed scaffolds could preserve the inhibition profile of the corresponding glucose-based *N*-alkyl derivatives as DNJ cores found in miglustat and miglitol drugs. Thus, a small library of iminosugars (14 compounds) displaying different stereochemistry, ring size, and *N*-substitutions was successfully synthesized from a common precursor, D-mannitol, by utilizing an S_N_2 aminocyclization reaction via two isomeric bis-epoxides. The evaluation of the prospective inhibitors on glucosidases revealed that merely D-*gluco*-piperidine (miglitol, **41a**) and L-*ido*-azepane (**41b**) DNJ-derivatives bearing the *N*-hydroxylethyl group showed inhibition towards α-glucosidase with IC_50_ 41 µM and 138 µM, respectively, using DNJ as reference (IC_50_ 134 µM). On the other hand, β-glucosidase inhibition was achieved for glucose-inverted configuration (C-2 and C-5) derivatives, as novel L-*gulo*-piperidine (**27a**) and D-*manno*-azepane (**27b**), preserving the *N*-butyl chain, with IC_50_ 109 and 184 µM, respectively, comparable to miglustat with the same *N*-butyl substituent (**40a**, IC_50_ 172 µM). Interestingly, the seven-membered ring L-*ido*-azepane (**40b)** displayed near twice the activity (IC_50_ 80 µM) of the corresponding D-*gluco*-piperidine miglustat drug (**40a**). Furthermore, besides α-glucosidase inhibition, both miglitol (**41a**) and L-*ido*-azepane (**41b**) proved to be the strongest β-glucosidase inhibitors of the series with IC_50_ of 4 µM.

## 1. Introduction

The major groups of glucosidase inhibitors that have been discovered are polyhydroxylated alkaloids containing piperidines, pyrrolidines, nor-tropanes, pyrrolizidines, and indolizidines as mono and bicyclic systems [1]. A great variety of these compounds, named iminosugars, have been isolated from natural sources, such as plants (*Morus alba*, *Commelina communis*), bacteria (*Bacillus, Streptomyces*), and fungi (*Zygosaccharomyces rouxii* for mulberry leaf fermentation) [2], and produced by synthetic strategies with potential inhibition properties not only over α- and β-glucosidases but also glycosyltransferases, glycogen phosphorylase [3,4,5], nucleoside phosphorylases [6], and sugar-nucleotide mutases (UDP-Galp mutase) [7]. High activity and specificity of iminosugars are associated with the ability of the nitrogen ring to mimic the transition state of pyranosidic or furanosidic units of natural glucosidase substrates that positively influence their shape and charge for enzyme binding.

**Figure 1:**
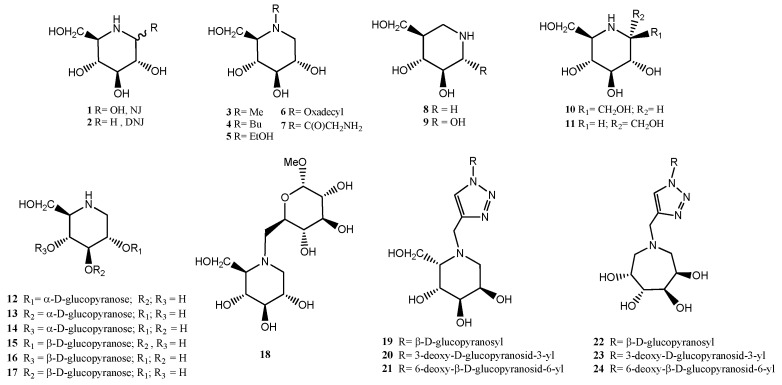
Examples of polyhydroxylated piperidine and azepane iminosugars reported, some of them displaying glucosidase inhibition.

Access to iminosugar analogues with *N*-substituted side chains has led to a variety of potent glucosidase and glycosyltransferases inhibitors with broad therapeutic applications, such as treatment of diabetes [8] and Gaucher disease [9], and even immunosuppressive activities [10] and antibacterial [11] and antiviral effects [12,13] against HIV [14], HPV [15], hepatitis C [16], bovine diarrhea (BVDV) [17], Ebola (EBOV) [18] and Marburg viruses (MARV) [19], influenza [20], Zika [21], and dengue virus [22,23]. Despite the α- and β-glucosidase inhibition promoted by nojirimycin itself (**1**), it was achieved a better profile for the corresponding 1-deoxynojirimycin (DNJ, **2**) due to better stability and potency. Furthermore, a combination of these structural features can promote the cellular uptake of *N*-alkylated DNJ analogues, as shown by those containing long and linear alkyl chains, which displayed better activity in whole cells (human hepatoblastoma cells, HepG2) than purified pork glucosidase I [24]. Conversely, *N*-alkyl-less lipophilic *N*-alkyl groups, or even containing an oxygen atom, displayed lower cytotoxicity and significant activity against α-glucosidase, as described for *N*-methyl- (**3**) [25], *N*-butyl- (*N*-Bu-DNJ, miglustat, **4**) [26], *N*-hydroxyethyl- (*N*-EtOH-DNJ, miglitol, **5**), *N*-7-oxadecyl- (*N*-7-oxadecyl-DNJ, **6**) [27,28], and *N*-glycyl-deoxynojirimycin (**7**) [29]. In fact, miglustat is particularly useful in the control of type I Gaucher disease [9] and Niemann–Pick type C (NPC) lysosomal storage diseases, via “substrate reduction therapy”, as well as miglitol in the treatment of non-insulin-dependent diabetes (type II) (Figure 1) to impair carbohydrate processing in the gut [30]. In addition, inhibition of the target human acid β-glucosidase (glucocerebrosidase, GCase) has been achieved by a set of derived iminosugars as new pharmacological chaperones for the treatment of Gaucher disease [31,32].

Besides the *N*-alkyl variations, several studies have pointed to the impact of the modification of DNJ hydroxyl groups involving C-2 to C-6 positions, assessing the influence of both stereochemistry and substituent variations on glycosidase activities. In general, the loss of α-glucosidase (I and II) and ceramide glycosyltransferase activities was evident by modifying C-2, C-3, and C-4, with the exception of *N*-butyl-1-deoxy-galactonojirimycin (migalastat, used for the treatment of Fabry disease) [30]. On the other hand, changes at C-1 and the ring nitrogen were allowed, based on the high inhibition revealed by DNJ and 1-azasugars (isofagomine (**8**), for instance), the latter obtained by replacing the anomeric carbon and the ring oxygen of glucose by nitrogen and carbon, respectively, with significant activity only against β-glucosidase. Interestingly, introduction of a hydroxyl group at the carbon (C-2) neighboring the nitrogen afforded a potent α-glucosidase inhibitor (noeuromycin, **9**) that preserved the original β-glucosidase activity. Additionally, the extra hydroxymethylene group at the anomeric position of DNJ gave rise to α-homonojirimycin (**10**) and β-homonojirimycin (**11**) with the ability to inhibit α-glucosidase, which was even higher whilst bearing *N*-methyl or *N*-butyl substituents (Figure 1) [1]. Furthermore, seven-membered ring iminosugar have shown potential glucosidase inhibition [33,34,35]. Comprehensive studies on iminosugar derivatives can be found in literature reviews [36,37,38,39].

Inspired by a series of reported deoxynojirimycin disaccharides that were decorated with equal α- or β-glucopyranose units at C-2, C-3, and C-4 DNJ positions (**12**-**17**) [40,41], along with *N*-glycosylated deoxynojirimycin, MDL 73,945 (**18**) [42], we had reported an alternative approach, using functionalized isomeric 1,5-dideoxy-1,5-imino-D-gulitol (L-*gulo*-piperidines, with inverted configurations at C-2 and C-5 with respect to glucose or DNJ, **19**-**21**) and 1,6-dideoxy-1,6-imino-D-mannitol (D-*manno*-azepane derivatives, **22**-**24**) cores *N*-linked to different sites of glucopyranose units, such as C-1, C-3, and C-6 positions [43]. To reach this goal, we used a CuAAC reaction (copper azide alkyne cycloaddition reaction), as a click chemistry strategy, to connect six- and seven-membered iminosugars to glucose in different arrangements through triazole bridges to produce the most active α-glucosidase inhibitor (**21**) of the pseudo-disaccharide series, L-gulopiperidine attached to glucose C-6 position, with IC_50_ approximately three-fold lower than that of DNJ (Figure 1) [43].

Despite the reported loss of α-glucosidase activity under modification at the C-5 position of the iminosugar, such as displayed by 1-deoxy-L-*ido*-nojirimycin (with an inverted configuration at C-5 with respect to DNJ) [24] and for 1,5-dideoxy-1,5-iminoxylitol (lacking the C-5 hydroxymethyl group of DNJ) [27,28], we have been encouraged to continue our studies on L-*gulo*-piperidines based on the remarkable α-glucosidase inhibition previously obtained for pseudo-disaccharides with simultaneous inverted configurations at C-2 and C-5 positions in relation to glucose stereochemistry [43]. Thus, to understand the relative contribution of the ring size, stereochemistry, and *N*-alkyl substitution on glycosidase inhibition, we proceeded with the synthesis of a small library of *N*-substituted 1-deoxy-L-*gulo*-nojirimycin and D-*manno*-azepane derivatives and compared them with the corresponding classical *N*-substituted of 1-deoxy-D-*gluco*-piperidine (DNJ) and L-*ido*-azepane counterparts as glucose-type carbohydrate mimetics. To reach this goal, *N*-hydroxyethyl and *N*-butyl groups of miglitol and miglustat drugs, respectively, were investigated as highly important *N*-alkyl substitutions for glucosidase inhibition, besides *N*-phenethyl [44], and *N*-propynyl [43] as a less-active counterpart.

## 2. Results and Discussion

### 2.1. Chemistry

**Scheme 1:**
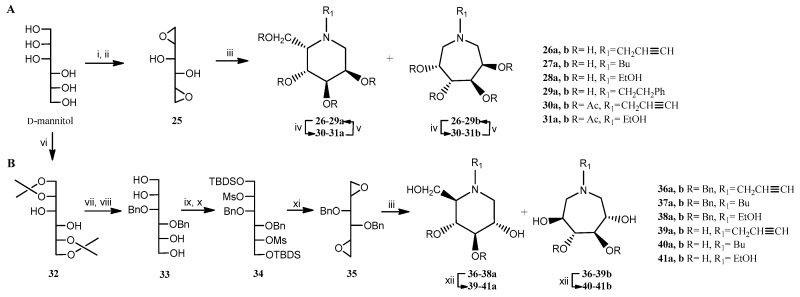
(**A**) Synthesis of iminosugars **26**-**29a** and **26**-**29b** from D-mannitol, via bis-epoxide **25**. Reagents and conditions: (i) TsCl, py, 71%; (ii) NaOH, CH_3_CN:H_2_O, 40 °C, 29%; (iii) Primary amine: propargylamine, butylamine, ethanolamine, or phenethylamine, MeOH, MW, 90 °C; (iv) Ac_2_O, Py; for yields over two steps see Table 1; (v) NaOMe, MeOH (quant). (**B**) Synthesis of iminosugars **39**-**41a** and **39**-**41b** from D-mannitol, via bis-epoxide **35;** (vi) 2,2-dimethoxypropane, TsOH, 96%; (vii) NaH, BnBr, *n*-Bu_4_NI, THF, 93%; (viii) HCl, MeOH, 0 °C, (quant); (ix) TBDMS chloride, imidazole, DMF, 0 °C, 88%; (x) MsCl, NEt_3_, DCM, 92%; (xi) HCl, MeOH, then NaOH, H_2_O, 70%; and (xii) TMSI, DCM, rt, then MeOH, 45–100%.

Initially, target 1-deoxy-L-*gulo*-nojirimycin (**26**-**29a**) and D-*manno*-azepane derivatives (**26**-**29b**) were synthesized by a regiospecific C_2_-symmetric unprotected bis-epoxide opening strategy in the presence of primary amines, followed by an S_N_2 aminocyclization reaction to give a mixture of both six- and seven-membered iminosugar isomers [45,46,47]. As previously reported, the synthesis of unprotected bis-epoxide **25** was promptly achieved from the simple and commercially available starting material, D-mannitol, in two steps, by tosylation of both D-mannitol primary alcohols (71%), followed by a base-promoted intramolecular S_N_2 reaction to give 1,2:5,6-dianhydro-D-mannitol **25** (29%), Scheme 1A [43]. Opening of the homochiral C_2_-symmetric bis-epoxide **25** by alkylamines (at the less-hindered position of one epoxy function) led to the formation of secondary amines, which promoted an S_N_2 aminocyclization reaction to give a mixture of polyhydroxy-piperidine and -azepane by 6-exo-tet or 7-endo-tet processes, respectively. In this series, azepane was isolated in a slightly higher proportion than DNJ, indicating the free 3,4 diol in bis-epoxide **25** did not affect the regioselectivity. Conversely, higher yields of DNJ than azepane derivatives were previously reported using benzyl protecting groups at C-3 and C-4 of bis-epoxide in the presence of several amines (benzylamine, for instance) [46]. Interestingly, this ratio can be reversed in the presence of a Lewis acid (perchloric acid), which catalyzes epoxide opening to mainly give azepane derivatives. Furthermore, the exclusive formation of seven-membered azasugars using a more rigid *trans*-acetonide protecting group, as observed in 1,2:5,6-dianhydro-3,4-*O*-isopropylidene-D-mannitol or L-iditol, led to the conclusion that formation of both polyhydroxy-piperidine and -azepane regioisomers can be achieved by an aminocyclization of a flexible bis-epoxide bearing a free or acyclic hydroxyl protecting groups at C-3 and C-4, and the ratio varies according to the experimental conditions [46].

Therefore, the microwave-assisted aminocyclization reaction of bis-epoxide **25** was carried out with four different primary amines (propargylamine, butylamine, ethanolamine, or phenethylamine), which resulted in a mixture of *N*-alkyl substituted polyhydroxypiperidine **26**-**29a** and azepane **26b**-**29b** regioisomers, being iminosugars **27a,b** and **28a,b** novel compounds. To prevent laborious separation of **a** and **b** regioisomers, some mixtures were treated with acetic anhydride and pyridine for prompt separation of the per-*O*-acetylated 1-deoxy-L-*gulo*-nojirimycin and D-*manno*-azepane by column chromatography, isolated in different ratio and yields over two steps, as depicted in Table 1. However, the separation of protected regioisomers **31a,b** was more demanding and required HPLC purification, mainly because the deprotected products **28a,b** were inseparable by chromatographic column or even HPLC under test conditions. Lastly, deprotection of the regioisomers in the presence of sodium methoxide gave products **26**-**29a** and **26**-**29b** in quantitative yields. Eventually, derivatives **27a,b** and **29a,b** bearing a more lipophilic side chain (butyl and phenethyl, respectively) were separated without the need of previous protection by using chromatography column eluted with DCM/MeOH (4:1). However, the yields of pure polyhydroxy-piperidines and -azepanes were much lower (approximately 10%, 0.8:1 ratio, respectively) than using protection/deprotection strategies (Table 1).

In order to keep the stereo-control during the reaction and obtain the iminosugars with the same stereochemistry of glucose, we pursued the classical procedure based on the protection of 1,2- and 5,6- positions of D-mannitol to produce the diisopropylidene intermediate **32** [48], which was benzylated at 3,4- positions and then deprotected under acid catalysis to give compound **33** (Scheme 1B) [47]. Briefly, selective protection of primary hydroxyl functions with bulk groups, followed by activation of the O-2 and O-5 with mesyl chloride and treatment of **34** in MeOH with concentrated HCl allowed the preparation of bis-epoxide **35** because of the intramolecular attack of the released primary hydroxyl functions that displaces the leaving mesyl groups. Then, bis-epoxide **35**, comprising inverted configurations at C-2 and C-5 comparatively to **25**, was converted to the corresponding mixture of *N*-substituted 1-deoxy-D-*gluco*-nojirimycin (**36**-**38a**) and 1,6-dideoxy-1,6-imino-L-*ido*-azepane derivatives (**36**-**38b**) in approximately 1:1 ratio under treatment with propargylamine, butylamine, ethanolamine, or phenethylamine for the aminocyclization reaction, as described for bis-epoxide **25**. Attempts to generate the *N*-phenethyl derivative of this series were unsuccessful since D-glucitol was isolated as a major product, possibly because phenethylamine promoted a regioselective opening of partially protected 1,2-epoxide (**35**) and then an *O*-cyclization leading to glucitol, as reported using ammonium formate [49].

After chromatographic separation of regioisomers, removal of the benzyl groups was better achieved under treatment with trimethylsilyl iodine [50] rather than hydrogenation conditions [47] to give final products **39**-**41a**-**b** in moderate to quantitative yields (45–100%).

### 2.2. Biological Assays

Initially, the small library of iminosugar derivatives (**26-29a,b** and **39-41a,b**) was screened for α-glucosidase inhibition (from *Saccharomyces cerevisiae*) activities using *p*-nitrophenyl α-D-glucopyranoside as substrate and prospective inhibitors at 1.0 mM concentration. To broaden the scope of the analysis, β-glucosidase (almond) activity of the same set of compounds was conducted using the corresponding *p*-nitrophenyl β-D-glucopyranoside.

#### 2.2.1. Yeast α-glucosidase Activities

Based on the IC_50_ values using α-glucosidase, the greatest inhibition was verified for both piperidine and azepane DNJ derivatives bearing the *N*-hydroxylethyl group, D-*gluco*-piperidine (miglitol, **41a**, IC_50_ 41 µM) and L-*ido*-azepane (**41b**, IC_50_ 138 µM), using the DNJ as the reference (IC_50_ 134 µM) (Table 2). The α-glucosidase inhibition promoted by L-*ido*-azepane **41b** was significant and related to DNJ, although with a three-fold lower activity than D-*gluco*-piperidine (**41a**). In spite of finding a patent for azepane **38b**, the data were inaccessible [51], and mixed results were found for nonsubstituted L-*ido*-azepane with inhibition properties (*K*_i_ 4.8 µM) lower than the corresponding D-*gluco*-piperidine (DNJ, *K*_i_ 0.44 µM) assayed on *Bacillus stearothermophilus* α-glucosidase [46] and high (*K*_i_ 29.4 µM) [52] to weak activity (IC_50_ 772 µM [33] or 35% inhibition at 1 mM [53]) using yeast α-glucosidase. In addition, weak or no α-glucosidase inhibition was observed for *N*-propynyl (**39a,b**) and *N*-butyl (**40a,b**) DNJ and azepane derivatives in these assays, confirming reported data for **39a** and miglustat (**40a**) [54] and **40b** (14% inhibition at 1 mM) [33] both using yeast α-glucosidase.

In respect to L-*gulo*-piperidine and D-*manno*-azepane series, with inverted configurations at C-2 and C-5, derivatives (**26-29a,b**) bearing *N*-hydroxyethyl, *N*-butyl, *N*-propynyl [43], or *N*-phenethyl chains on the endocyclic nitrogen proved to be inactive against yeast α-glucosidase at the tested concentration (15–2000 µM), leading to loss of activity even for the *N*-hydroxyethyl derivatives (**28a,b**) when compared to **41a,b**. Reported α-glucosidase inhibition data for nonsubstituted L-*gulo*-piperidine and D-*manno*-azepane were found as weak as 30% and 55%, respectively, tested at 1 mM in *Bacillus stearothermophilus* [46] or 21% in yeast α-glucosidase at 240 µM [52]. Thus, it was evident that α-glucosidase activities were considerably affected by iminosugar stereochemistry, ring size, and *N*-substitutions, and inversion of configuration was detrimental for activity regardless of the *N*-substituents here described, suggesting the wrong orientation of at least two hydroxyl groups attached at C-2 and C-5, which led to reduced binding affinity at yeast α-glucosidase active sites.

#### 2.2.2. Almond β-glucosidase Activities:

Conversely, the assessment of the series on β-glucosidase revealed that novel L-*gulo*-piperidine (**27a**) and D-*manno*-azepane (**27b**) derivatives (inverted configurations at C-2 and C-5 related to glucose) preserving the *N*-butyl chain showed significant activity, IC_50_ 109 and 184 µM, respectively, comparable to miglustat (**40a**, IC_50_ 172 µM), although three- to five-fold lower than DNJ (IC_50_ 33 µM) (Table 2). In this particular case, the *N*-butyl chain seems to play an important role in β-glucosidase inhibition since the reported data for nonsubstituted was low for both L-*gulo*-piperidine (13% at 1 mM) and D-*manno*-azepane (1% at 1 mM) or no inhibition at 240 µM on the same enzyme [46,52]. Interestingly, for the same set that preserve the *N*-butyl chain but display glucose stereochemistry, the seven-membered ring derivative L-*ido*-azepane **40b** displayed nearly twice the activity (IC_50_ 80 µM) of the corresponding D-*gluco*-piperidine drug (**40a**), which resembled the stronger β-glucosidase inhibition achieved for nonsubstituted L-*ido*-azepane (*K*_i_ 17 µM [46], 12.8 µM [52], or IC_50_ 38 µM [53]) than nonsubstituted D-*gluco*-piperidine (*K*_i_ 1700 µM) [46]. Furthermore, both derivatives bearing the *N*-hydroxyethyl chain, as occurs in miglitol (**41a**) and L-*ido*-azepane **41b,** proved to be the strongest β-glucosidase inhibitors with IC_50_ of 4 µM. Based on all these findings, it was possible to infer that almond β-glucosidase active sites can accept and interact with a wider range of iminosugars than yeast α-glucosidase.

See dose-response curves obtained from Yeast α-Glucosidase and Almond β-Glucosidase assays in Supplementary Materials.

## 3. Conclusions

In summary, a series of iminosugars were successfully synthesized from a common precursor, D-mannitol, to produce two alternative bis-epoxides, further modified by an S_N_2 aminocyclization reaction to give a mixture of both *N*-substituted six- and seven-membered iminosugar isomers. Besides the ring size, two additional structural variations were also pursued to broaden the scope of reported strategies, as stereochemistry (maintenance of glucose stereochemistry or inversion of configuration at C-2 and C-5 positions) and *N*-chain of the endocyclic nitrogen (*N*-propynyl, -butyl, -hydroxyethyl, and -phenethyl). Classical polyhydroxypiperidines, miglustat and miglitol drugs that maintain glucose configuration (D-*gluco*-nojirimycin, DNJ) and bear *N*-butyl and *N*-hydroxyethyl chains, respectively, were synthesized and used as reference for evaluation of the series towards α- and β-glucosidases. Assessment of α-glucosidase activity of iminosugars revealed solely miglitol as the most active of the series, followed by the corresponding L-*ido*-azepane isomer. All other iminosugars proved to not be inhibitors of yeast α-glucosidase. On the other hand, all *N*-butyl iminosugars having either glucose stereochemistry, D-*gluco*-piperidine miglustat drug and L-*ido*-azepane, or inverted configurations at C-2 and C-5 related to glucose, L-*gulo*-piperidine and D-*manno*-azepane derivatives, displayed significant inhibition of almond β-glucosidase. In spite of that, the strongest inhibition was achieved for D-*gluco*-piperidine miglitol drug and the corresponding L-*ido*-azepane iminosugars containing *N*-hydroxyethyl chains, but, in these tests, no activity was accomplished for inverted configuration counterparts. Thus, we observed that glucosidase inhibition promoted by some polyhydroxypiperidines was accompanied by proportional inhibition of the corresponding polyhydroxyazepane isomers bearing the same *N*-chain regardless of the ring stereochemistry. In addition, the findings of this study on β-glucosidase inhibition by L-*gulo*-piperidine and D-*manno*-azepane series are relevant considering their straightforward synthesis compared to DNJ series.

## 4. Material and Methods

^1^H (300 MHz) and ^13^C (75 MHz) NMR spectra were recorded on a Bruker^®^ Ultrashield 300 NMR spectrometer. ^1^H (400 MHz) and ^13^C (100 MHz) NMR spectra were recorded on a Bruker^®^ Avance 400 MHz NMR spectrometer. ^1^H (500 MHz) and ^13^C (125 MHz) NMR spectra were recorded on a Bruker^®^ Avance 500 MHz NMR spectrometer. All spectra were recorded at room temperature (~20 °C) in Sigma Aldrich^®^ deuterated solvents. Chemical shifts (δ) were expressed in parts per million (ppm) relative to the reference peak. Coupling constants (*J*) were expressed in Hertz (Hz). Splitting patterns in ^1^H NMR spectra were designated as s (singlet), br s (broad singlet), d (doublet), br d (broad doublet), t (triplet), q (quartet), dd (doublet of doublets), dt (doublet of triplets), ddd (doublet of doublet of doublets), and m (multiplet). Optical rotations were measured on a Jasco P-2000 polarimeter at 22.5 nd using a sodium lamp and wavelength of 589 nm at 22.5 °C. HPLC purifications were designed in a Shimadzu^®^ SCL-10A HPLC system, Diode Array Detector Shimadzu^®^ SPD-M10A, and processed on Class-VP software. Purification of the compounds **31a** and **31b** was performed in HPLC using a Macherey–Nagel CLC-ODS semiprep column, Methanol 40%, flowrate 4.0 mL/min, and 200 nm. High-resolution mass spectra (HRMS) were obtained on a Bruker Daltonics MicrOTOF-Q II ESI-TOF mass spectrometer, and an Exactive Plus Orbitrap mass spectrometer (Thermo Scientific, Germany) was equipped with an electrospray (H-ESI-II) probe and operated in negative ionization mode. The system was controlled by Xcalibur and Tune (Thermo Scientific). For biological assays, absorbance at 405 nm was measured using SpectraMax M2 Molecular Devices^®^.

**1,2:5,6-di-anhydro-D-mannitol** (**25**) [43]: D-Mannitol (5.05 g, 27.4 mmol) was solubilized in pyridine (25.0 mL) and heated to 120 °C for 15 min. After that, the solution was refrigerated to 0 °C, treated dropwise with tosyl chloride solution (13.10 g, 68.7 mmol/10 mL pyridine) for 1 h, and stirred at 0 °C for 3 h then at r.t. for 1 h. The mixture was coevaporated with toluene, and the solid was diluted in dichloromethane and washed with HCl 1 mol·L^−1^ and saturated NaHCO_3_. The organic layer was dried over MgSO_4_ and concentrated. The crude mixture of 1,6-di-O-tosyl-D-mannitol was solubilized in a mixture of acetonitrile and water (38.0 mL, 2:1, v/v), and a small portion of phenolphthalein was added to it. The mixture was stirred at 35–40 °C and titrated with NaOH 5 mol·L^−1^ until the solution remained pink. After that, a mixture of Na_2_CO_3_ (87.4 g) in ethyl acetate (324 mL) was added and stirred vigorously. The solid was filtrated and washed with ethyl acetate. The organic solution was dried over MgSO_4_, filtered, and concentrated. The resulting mixture was purified by flash chromatography (hexane and ethyl acetate, 7:3, v/v) to yield **1** (1.12 g, 7.70 mmol, 28%). ^1^H NMR (400 MHz, CD_3_OD): δ 2.77 (2H, dd, J 2.7 Hz, 5.3 Hz, H-1a, H-6a); 2.82 (2H, dd, J 4.0 Hz, 5.3 Hz, H-1b, H-6b); 3.11–3.17 (2H, m, H-2, H-5); 3.46–3.52 (2H, m, H-3, H-4). ^13^C NMR (100 MHz, CDCl_3_): δ 46.1 (C-1, C-6); 52.8 (C-2, C-5); 73.3 (C-3, C-4).

**1,2:5,6-Di-*O*-isopropilidene D-mannitol (32)** [48]

**1,2:5,6-Dianhydro-3,4-di-O-benzyl-L-iditol (35)**: prepared as described by Wilkinson et al. [47]

**General procedure for the synthesis ofL-*gulo*-piperidine and D-*manno*-azepane derivatives(26a,b-31a,b):** 1,2:5,6-dianhydro-D-glucitol was solubilized in methanol and 2.5 eq of primary amine (propargylamine, ethanolamine, butylamine, or phenethylamine) was added to the solution. The mixture was heated to 90 °C in a microwave for 5 min (150 W) in a sealed vessel. The solvents were evaporated in vacuum. Compounds **27a,b** and **29a,b** were separated in a flash column (dichloromethane and methanol, 8:2 v/v). Compounds **26a,b** and **28a,b** were acetylated by the addition of pyridine (8.0 mL) and acetic anhydride (22.8 mL) and stirred at r.t. After 3 h, ice was added to the mixture, the product was extracted with ethyl ether, and the organic layer was evaporated and dried with MgSO_4_. Compounds **30a,b** were separated by column chromatography (toluene and ethyl acetate, 3:7, v/v). Compounds **31a,b** were purified in a HPLC C-18 semiprep column in methanol/water 40% and flow rate 4.0 mL/min. 

See ^1^H, ^13^C and bidimensional NMR and ESI HRMS spectra of compounds **26-31a,b** and **36-41a,b** in Supplementary Materials**.**

**General procedure for removal of Acetyl groups:** Compounds **30a,b** and **31a,b** were dissolved in methanol (1.0 mL), and sodium methoxide 1 M was added dropwise to the solution until pH 9.0 and checked with Tornassol. After half an hour, TLC showed total consumption of starting material, then it was neutralized with ion exchange resin DOWEX^®^ 50WX4-50. After that, the mixture was filtered through a Celite^®^ pad and concentrated in vacuo.

**General procedure for removal o Benzyl groups [50]**: The corresponding product was solubilized in dichloromethane, and Me_3_SI (4 eq) was added slowly. The reaction was allowed to stir at r.t. for 15 min. TLC showed total consumption of starting material, then the reaction was quenched with methanol. The product was purified in a SPE-C18 silica pad with methanol/water 1:1 v/v.

***N*-Propynyl-1,5-dideoxy-1,5-imino-L-gulitol (26a) [43]:** 80% (0.0082 g, 0.041 mmol): ^1^H NMR (500 MHz, D_2_O): δ 2.71–2.77 (2H, m, H-1a, ≡CH); 2.85 (1H, t, *J* 10.9 Hz, H-1b); 2.88–2.93 (1H, m, H-5); 3.46 (1H, d, *J* 17.6 Hz, H-7a); 3.68 (1H, d, *J* 17.6 Hz, H-7b); 3.83 (1H, dd, *J* 5.4 Hz, 11.7 Hz, H-6a); 3.89 (1H, dd, *J* 3.9 Hz, 11.7 Hz, H-6b); 3.91–3.94 (1H, m, H-3); 4.05–4.14 (2H, m, H-2, H-4). ESI HRMS: [M+H]^+^ calculated for C_9_H_15_NO_4_ 202.1074; found 202.1076.

***N*-Propynyl-1,6-dideoxy-1,6-imino-D-mannitol (26b)** [43]: 86% (0.0088 g, 0.044 mmol): ^1^H NMR (500 MHz, D_2_O): δ 2.66 (1H, t, *J* 2.2 Hz, ≡CH); 2.85 (4H, m, H-1a; H-1b; H-6a; H-6b); 3.37 (1H, d, *J* 17.0 Hz, H-7a); 3.41 (1H, d, *J* 17.0 Hz, H-7b); 3.89–3.91 (2H, m, H-3, H-4); 4.12 (2H, t, *J* 4.8 Hz, H-2, H-5). ESI HRMS: [M+H]^+^ calculated for C_9_H_15_NO_4_ 202.1074; found 202.1073.

***N*-Butyl-1,5-dideoxy-1,5-imino-L-gulitol (27a):**${\left[\alpha \right]}_{D}^{22.5}$ 29.5 (*c* 1.8, MeOH), ^1^H NMR (500 MHz, D_2_O): δ 0.83 (3H, t, *J* 7.4 Hz, H-10); 1.17–1.28 (2H, m, H-9); 1.37–1.50 (2H, m, H-8); 2.50–2.92 (5H, m, 2xH-1, H-5, 2xH-7); 3.75 (1H, dd, *J* 5.6. 11.8, H-6a); 3.78–3.85 (2H, m, H-3, H-6b); 3.94–4.00 (1H, m, H-2); 4.05–4.11 (1H, m, H-4). ^13^C NMR (125 MHz, D_2_O): δ 13.3 (C-10); 20.0 (C-9); 27.4 (C-8); 53.1 (C-1 or C-7); 55.1 (C-1 or C-7); 58.5 (C-6); 68.2 (C-4); 70.1 (C-3); 70.4 (C-3); 72.8 (C-2). ESI HRMS: [M+H]^+^ calculated for C_10_H_22_NO_4_ 220.1544; found 220.1538. 

***N*-Butyl-1,6-dideoxy-1,6-imino-D-mannitol (27b):**${\left[\alpha \right]}_{D}^{22.5}$ −62.0 (*c* 2.2, MeOH), ^1^H NMR (500 MHz, D_2_O): δ 0.76 (3H, t, *J* 7.3 Hz, 3xH-10); 1.07–1.25 (2H, m, 2xH-9); 1.30–1.44 (2H, m, 2xH-8); 2.47–2.57 (2H, m, 2xH-7); 2.65–2.85 (4H, m, 2xH-1, 2xH-6); 3.73–3.79 (2H, m, H-3, H-4); 3.94–4.04 (2H, m, H-2, H-5). ^13^C NMR (125 MHz, D_2_O): δ 13.2 (C-10); 20.0 (C-9); 27.5 (C-8); 55.2 (C-1, C-6); 58.4 (C-7); 68.3 (C-2; C-5); 72.8 (C-4; C-3). ESI HRMS: [M+H]^+^ calculated for C_10_H_22_NO_4_ 220.1544; found 220.1543.

***N*-Hydroxyethyl-1,5-dideoxy-1,5-imino-L-gulitol (28a):**${\left[\alpha \right]}_{D}^{22.5}$ 16.1 (*c* 0.6, MeOH), ^1^H NMR (400 MHz, CD_3_OD): δ 2.53–2.71 (2H, m, H-1a, H-7a); 2.78–2.89 (2H, m, H-5, H-7b); 2.97 (1H, ddd, *J* 4.9; 7.0; 13.5 Hz, H-1b); 3.55–3.98 (7H, m, H-2, H-3, H-4, H-6a, H-6b, H-8a, H-8b). ^13^C NMR (100 MHz, CD_3_OD): δ 51.9 (C-7); 55.1 (C-1); 58.6 (C-8); 60.0 (C-6); 61.0 (C-5); 66.5, 70.9, 71.3 (C-2, C-3, C-4). ESI HRMS: [M+H]^+^ calculated for C_8_H_18_NO_5_ 208.1180; found 208.1177

***N*-Hydroxyethyl-1,6-imino-D-mannitol (28b):**${\left[\alpha \right]}_{D}^{22.5}$ −15.3 (*c* 0.5, MeOH), ^1^H NMR (400 MHz, CD_3_OD): δ 2.58–2.80 (4H, m, H-1a; H-6ª; 2xH-7); 2.89 (2H, dd, *J* 4.3; 13.2 Hz, H-1b, H-6b); 3.59 (2H, dd, *J* 6.0; 12.5 Hz, 2xH-8); 3.86–3.92 (2H, m, H-3, H-4); 4.00–4.08 (2H, m, H-2, H-5). ^13^C NMR (100 MHz, CD_3_OD): δ 58.4 (C-1; C-6); 60.4 (C-8); 61.8 (C-7); 70.9 (C-2; C-5); 74.0 (C-3; C-4). ESI HRMS: [M+H]^+^ calculated for C_8_H_18_NO_5_ 208.1180; found 208.1182. 

***N*-Phenethyl-1,5-dideoxy-1,5-imino-L-gulitol (29a)** [46]

***N*-Phenethyl-1,6-dideoxy-1,6-imino-D-mannitol (29b)** [46]

***N*-Propynyl-2,3,4,6-tetra-*O*-acetyl-1,5-dideoxy-1,5-imino-L-gulitol (30a) [43]**: ^1^H NMR (400 MHz, CDCl_3_): δ 2.03, 2.09, 2.13, 2.14 (12H, 4s, 4×CH_3_); 2.33 (1H, t, *J* 2.3 Hz, ≡CH); 2.80 (1H, dd, *J* 4.7 Hz, 11.2 Hz, H-1a); 2.97 (1H, dd, *J* 9.6 Hz, 11.2 Hz, H-1b); 3.28 (1H, ddd, *J* 3.3 Hz, 5.8 Hz, 10.0 Hz, H-5); 3.44 (1H, dd, *J* 2.3 Hz, 17.8 Hz, H-7a); 3.67 (1H, dd, *J* 2.3 Hz, 17.8 Hz, H-7b); 4.20 (1H, dd, *J* 5.8 Hz, 11.6 Hz, H-6b); 4.24 (1H, dd, *J* 3.3 Hz, 11.6 Hz, H-6a); 5.20–5.27 (3H, m, H-2, H-3, H-4). ^13^C NMR (100 MHz, CDCl_3_): δ 20.8; 20.9 (CH_3_); 43.9 (C-1); 49.6 (C-7); 55.5 (C-5); 61.4 (C-6); 66.5 (C-3, C-4); 68.7 (C-2); 77.2 (≡CH); 169.3; 169.7; 170.5 (C=O). ESI HRMS: [M+H]^+^ calculated for C_17_H_24_NO_8_ 370.1496; found 370.1496.

***N*-Propynyl-2,3,4,6-tetra-*O*-acetyl-1,6-dideoxy-1,6-imino-D-mannitol (30b)** [43]: ^1^H NMR (400 MHz, CDCl_3_): δ 2.05, 2.12 (12H, 2s, 4×CH_3_); 2.25 (t, *J* 2.3 Hz, ≡CH); 2.91 (2H, dd, *J* 5.6 Hz, 13.8 Hz, H-1a, H-6a); 2.99 (2H, dd, *J* 4.4 Hz, 13.8 Hz, H-1b, H-6b); 3.39 (1H, dd, *J* 2.3 Hz, 17.3 Hz, H-7a); 3.45 (1H, dd, *J* 2.3 Hz, 17.3 Hz, H-7b); 5.40 (2H, dt, *J* 1.2 Hz, 4.6 Hz, H-2, H-5); 5.48 (2H, t, *J* 1.2 Hz, H-3, H-4). ^13^C NMR (100 MHz, CDCl_3_): δ 20.8; 21.0 (CH_3_); 47.9 (C-7); 54.1 (C-1, C-6); 69.9 (C-2, C-5); 70.9 (C-3 e C-4); 77.2 (≡CH); 169.9; 170.1 (C=O). ESI HRMS: [M+H]^+^ calculated for C_17_H_24_NO_8_ 370.1496; found 370.1535.

***N*-Acetoxyethyl-2,3,4,6-tetra-*O*-acetyl-1,5-dideoxy-1,5-imino-L-gulitol (31a):**^1^H NMR (400 MHz, CDCl_3_): δ 2.06; 2.08; 2.10 (18H, 3s, 5xCH_3_); 2.89 (1H, dd, *J* 5.0; 13.7 Hz, H-1a); 2.96 (2H, t, H-7, *J* 5.9 Hz, H-7); 3.04 (1H, dd, *J* 2.6; 13.7 Hz, H-1b); 3.45 (1H, dd, *J* 4.8; 11.4 Hz, H-5); 4.05 (1H, dd, *J* 5.6; 11.3 Hz, H-6a); 4.10–4.21 (2H, m, 2xH-8); 4.37 (1H, dd, *J* 7.0; 11.8 Hz, H-6b); 5.15 (1H, dd, *J* 3.3; 9.0 Hz, H-3); 5.20–5.25 (1H, m, H-2); 5.20–5.25 (1H, m, H-2); 5.28 (1H, dd, *J* 4.9; 9.0 Hz, H-4). ^13^C NMR (100 MHz, CDCl_3_): δ 20.8, 20.9, 21.0 (5xCH_3_); 48.9 (C-1); 52.7 (C-7); 58.2 (C-5); 59.9 (C-6); 68.0 (C-3); 68.1, 68.3 (C-2, C-4).

***N*-Acetoxyethyl-2,3,4,6-tetra-*O*-acetyl-1,6-dideoxy-1,6-imino-D-mannitol (31b):**^1^H NMR (400 MHz, CDCl_3_): δ 2.03; 2.07; 2.10 (18H, 3s, 5xCH_3_); 2.85 (2H, t, J 5.8 Hz, H-4, H-4^′^); 2.91 (2H, dd, *J* 5.6; 14.0 Hz, H-1a, H-1^′^a); 3.04 (2H, dd, *J* 4.3; 14.0 Hz, H-1b, H-1^′^b); 4.11 (2H, dd, J 5.7; 9.1 Hz, H-5, H-5^′^); 5.29-5.37 (2H, m, H-2, H-2^′^); 5.43-5.47 (2H, m, H-3, H-3^′^). ^13^C NMR (100 MHz, CDCl_3_): δ 20.8; 20.9; 21.0 (5xCH_3_); 54.8 (C-1, C-1^′^); 56.4 (C-4); 62.2 (C-5); 70.2 (C-2, C-2^′^); 70.6 (C-3, C-3^′^).

***N*-Propynyl-2,3,4,6-tetra-*O*-acetyl-1,5-dideoxy-1,5-imino-D-glucitol (36a):**^1^H NMR (400 MHz, CDCl_3_): δ 2.27 (1H, t, *J* 2.2 Hz, ≡CH); 2.50 (1H, d, *J* 9.5 Hz, H-5); 2.60 (1H, t, *J* 10.7 Hz, H-1a); 2.92 (1H, dd, *J* 4.9; 10.9 Hz, H-1b); 3.31–3.44 (2H, m, 2xH-7); 3.62–3.91 (5H, m, H-2, H-3, H-4, 2xH-6); 4.77 (2H, dd, *J* 3.8; 11.2 Hz, CH_2_Ph); 4.98 (2H, dd, *J* 11.2; 13.4 Hz, CH_2_Ph); 7.29–7.45 (10H, m, H-Ph). ^13^C RMN (100 MHz, CDCl_3_): δ 42.1 (C-1); 56.1 (C-7); 57.3 (C-6); 63.1 (C-2 or C-5); 69.4 (C-2 or C-5); 74.6 (C≡CH); 75.2 (CH_2_Ph); 87.3 (C-3; C-4); 127.9, 128.6, 128.7 (Ar); 138.1 (C_q_). ESI HRMS: [M+H]^+^ calculated for C_23_H_28_NO_4_ 382.2013; found 382.2002.

***N*-Propynyl-2,3,4,6-tetra-*O*-acetyl-1,6-dideoxy-1,6-imino-L-iditol (36b):**^1^H NMR (400 MHz, CDCl_3_): δ 2.31 (1H, t, *J* 2.3 Hz, ≡CH); 2.77 (2H, dd, J 8.2; 12.3 Hz, H-1a, H-6a); 2.98 (2H, dd, *J* 1.1; 12.7, H-1b, H6b); 3.40–3.56 (2H, m, 2xH-7); 3.63–3.70 (2H, m, H-3, H-4); 3.80–3.93 (2H, m, H-2, H-5); 4.67 (2H, d, *J* 11.2 Hz, CH_2_Ph); 4.81 (2H, d, *J* 11.2 Hz, CH_2_Ph); 7.29–7.42 (10H, m, H-Ph). ^13^C RMN (100 MHz, CDCl_3_): δ 48.8 (C-7); 56.9 (C-1, C-6); 68.0 (C-2, C-5); 73.8 (CH_2_Ph); 78.2 (C≡CH); 86.5 (C-3, C-4); 127.9, 128.0, 128.6 (Ar); 137.9 (C_q_). ESI HRMS: [M+H]^+^ calculated for C_23_H_28_NO_4_ 382.2013; found 382.2001.

***N*-Butyl-2,3,4,6-tetra-*O*-acetyl-1,5-dideoxy-1,5-imino-D-glucitol (37a):**^1^H NMR (400 MHz, CDCl_3_): δ 0.94 (3H, t, *J* 6.9 Hz, 3xH-10); 1.21–1.38 (2H, m, 2xH-9); 1.39–1.56 (2H, m, 2xH-8); 2.26 (1H, t, *J* 10.6Hz, H-5); 2.32–2.54 (3H, m, H-1a, H-1b, H-7a); 2.70–2.82 (1H, m, H-7b); 3.13 (1H, dd, *J* 4.6; 11.2 Hz, H-6a); 3.38 (1H, t, *J* 8.8 Hz, H-3); 3.60–3.73 (2H, m, H-4, H-6b); 3.77–3.93 (2H, m, H-2, H-XX); 4.75 (2H, dd, *J* 5.6; 11.2 Hz, CH_2_Ph); 4.96 (2H, t ap., *J* 11.0 Hz, CH_2_Ph); 7.29–7.48 (10H, m, Ar). ^13^C RMN (100 MHz, CDCl_3_): δ 14.0 (C-10); 20.6 (C-9); 27.3 (C-8); 52.1 (C-1 or C-7); 55.1 (C-1 or C-7); 57.5 (C-6); 64.9 (C-2 or C-5); 69.3(C-2 or C-5); 74.9 (CH_2_Ph); 75.1 (CH_2_Ph); 78.1 (C-3 or C-4); 86.8 (C-3 or C-4); 127.8, 127.9, 128.0, 128.5, 128.7 (C-Ar); 138.1 (C_q_); 138.5 (C_q_). ESI HRMS: [M+H]^+^ calculated for C_24_H_34_NO_4_ 400.2483; found 400.2477.

***N*-Butyl-2,3,4,6-tetra-*O*-acetyl-1,6-dideoxy-1,6-imino-L-iditol (37b):**^1^H NMR (400 MHz, CDCl_3_): δ 0.93 (3H, t, *J* 7.2 Hz, 3xH-10); 1.25–1.40 (2H, m, 2xH-9); 1.42–1.58 (2H, m, 2xH-8); 2.52–2.69 (4H, m, H-1a, H-6a, 2xH-7); 2.92 (2H, d, *J* 12.5 Hz, H-1b, H-6b); 3.60–3.70 (2H, m, H-3, H-4); 3.78–3.89 (2H, m, H-2, H-5); 4.66 (2H, d, *J* 11.2 Hz, CH_2_Ph); 4.79 (2H, d, *J* 11.2 Hz, CH_2_Ph); 7.29–7.43 (10H, m, H-Ph). ^13^C RMN (100 MHz, CDCl_3_): δ 13.9 (C-10); 20.4 (C-9); 29.3 (C-8); 57.6 (C-7); 59.0 (C-1; C-6); 67.7 (C-2, C-5); 73.6 (CH_2_Ph); 87.0 (C-3, C-4); 127.9, 128.6 (Ar); 137.0 (C_q_). ESI HRMS: [M+H]^+^ calculated for C_24_H_34_NO_4_ 400.2483; found 400.2475.

***N*-Hydroxyethyl-2,3,4,6-tetra-*O*-acetyl-1,5-dideoxy-1,5-imino-D-glucitol (38a):**^1^H NMR (400 MHz, CDCl_3_): δ 2.29 (1H, dd, *J* 9.7, 11.3 Hz, H-1a); 2.37–2.51 (2H, m, H-5, H-7a); 2.94–3.08 (1H, m, H-7b); 3.16 (1H, dd, *J* 4.4; 11.5 Hz, H-1b); 3.43 (1H, t, *J* 8.3 Hz, H-3); 3.54–3.77 (4H, m, H-2, H-4, H-8a, H-8b); 3.88 (2H, qd, *J* 2.8; 12.3 Hz, H-6a, H-6b); 4.67–4.81 (2H, m, CH_2_Ph); 4.84–4.97 (2H, m, CH_2_Ph); 7.29–7.45 (10H, m, Ph-H). ^13^C RMN (100 MHz, CDCl_3_): δ 53.2 (C-7); 55.3 (C-1); 57.8 (C-6); 59.6 (C-8); 65.7 (C-5); 69.0 (C-2 or C-4); 74.8 (CH_2_Ph); 78.0 (C-2 or C-4); 85.9 (C-3); 128.0, 128.6 (Ar); 138.4 (C_q_). ESI HRMS: [M+H]^+^ calculated for C_22_H_30_NO_5_ 388.2119; found 388.2120.

***N*-Hydroxyethyl-2,3,4,6-tetra-*O*-acetyl-1,6-dideoxy-1,6-imino-L-iditol (38b):**^1^H NMR (400 MHz, CDCl_3_): δ 2.66–2.80 (4H, m, 2xH-1, 2xH-2); 2.99 (2H, dd, *J* 2.8; 13.1 Hz, 2xH-7); 3.62–3.72 (4H, m, H-2, H-5, 2xH-8); 3.80–3.90 (2H, m, H-3, H-4); 4.65 (2H, d, *J* 11.3 Hz, CH_2_Ph); 4.84 (2H, d, *J* 11.3 Hz, CH_2_Ph); 7.28–7.42 (10H, m, H-Ph). ^13^C RMN (100 MHz, CDCl_3_): δ 59.0 (C-7); 59.7 (C-8); 60.7 (C-1; C-6); 70.0 (C-3; C-4); 74.2 (CH_2_Ph); 85.4 (C-2; C-5); 127.9, 128.0, 128.6, 137.9 (Ar). ESI HRMS: [M+H]^+^ calculated for C_22_H_30_NO_5_ 388.2119; found 388.2102.

***N*-Propynyl-1,5-dideoxy-1,5-imino-D-glucitol (39a)** [47]

***N*-Propynyl-1,6-dideoxy-1,6-imino-L-iditol (39b)** [47]

***N*-Butyl-1,5-dideoxy-1,5-imino-D-glucitol (40a)** [55]

***N*-Butyl-1,6-dideoxy-1,6-imino-L-iditol (40b):**${\left[\alpha \right]}_{D}^{22.5}$ 1.3 (*c* 1.1, MeOH), ^1^H NMR (400 MHz, D_2_O): δ 0.86 (3H, t, *J* 8.0 Hz, 3xH-10); 1.24–1.36 (2H, m, 2xH-9); 1.56–1.74 (2H, m, 2xH-8); 3.14–3.23 (2H, m, H-7); 3.26–3.37 (4H, m, 2xH-1, 2xH-6); 3.58–3.67 (2H, m, H-2, H-5); 4.01–4.08 (2H, m, H-3, H-4). ^13^C RMN (100 MHz, D_2_O): δ 12.9 (C-10); 19.3 (C-9); 25.6 (C-8); 58.6 (C-1; C-6; C-7) 67.1 (C-3 or C-4); 67.7 (C-3 or C-4); (C-2; C-5). ESI HRMS: [M+H]^+^ calculated for C_10_H_22_NO_4_ 220.1544; found 220.1545. 

***N*-Hydroxyethyl-1,5-dideoxy-1,5-imino-D-glucitol** (41a) [55]

***N*-Hydroxyethyl-1,6-dideoxy-1,6-imino-L-iditol (41b):**${\left[\alpha \right]}_{D}^{22.5}$ −9.1 (*c* 0.6, MeOH), ^1^H NMR (400 MHz, D_2_O): δ 2.62–2.77 (4H, m, H-1a; H-1b; H-6a; H-6b); 2.91 (2H, dd, *J* 3.8;13.7 Hz, H-7a, H-7b); 3.40–3.48 (2H, m, H-2, H-3); 3.69 (4H, m, H-4, H-5, H-8a, H-8b,). ^13^C RMN (100 MHz, D_2_O): δ 58.4 (C-7), 59.3 (C-1, C-6, C-8), 70.8 (C-4, C-5), 75.6 (C-2, C-3). ESI HRMS: [M+H]^+^ calculated for C_8_H_18_NO_5_ 208.1185; found 208.1176.

**Biological assays [43]**: Yeast α-glucosidase (EC 3.2.1.20) and almond β-glucosidase (EC 3.2.1.21) activity was assessed using a 96-well plate assay. Assays contained 20 mM NaOAc at pH 6.8 (α-glucosidase) and pH 6.2 (β-glucosidase), 10 mM PIPES (piperazine-N,N′-bis(2-ethanesulfonic acid), 0.1 mM EDTA, α-glucosidase (5 μg/mL), β-glucosidase (6 μg/mL), and inhibitor (0.1–2 mM). Enzyme and inhibitor were equilibrated at 37 °C for 30 min. The reaction was initiated by the addition of *p*-nitrophenyl α-D-glucopyranoside (200 μM) or *p*-nitrophenyl β-D-glucopyranoside (200 μM), and then it was quenched with 100 μL of sodium carbonate 3.0 M after 25 min incubation at 37 °C. Assays were repeated in duplicate and data averaged.

## Figures and Tables

**Table 1:** Yields obtained from microwave-assisted aminocyclization reaction of bis-epoxides **25** and **35**.

Primary Amine for Aminocyclization Reaction	Yield (%)			
	Polyhydroxy-Piperidine		Polyhydroxy-Azepane	
	1-deoxy-L-*gulo*-nojirimycin 26-29a	1-deoxy-D-*gluco*-nojirimycin (DNJ) 39-41a	D-*manno*-azepane 26-29b	L-*ido*-azepane 39-41b
Propargylamine	32	40	35	37
Butylamine	20	33	24	38
Ethanolamine	17	22	21	28
Phenethylamine*	4	**-**	5	**-**

* Low yields obtained when the reaction mixture was purified directly by chromatographic column, without previous acetylation.

**Table 2:** α- and β-Glucosidase activities of synthesized iminosugars having alternative stereochemistry, ring size, and *N*-alkyl and *N*-arylalkyl chains on the endocyclic nitrogen.

Iminosugars with Inverted Configuration at C-2 and C-5 with Respect to Glucose				Iminosugars Preserving Glucose Stereochemistry			
Inhibition (µM)				Inhibition (µM)			
		α-Glucosidase	β-Glucosidase			α-Glucosidase	β-Glucosidase
**-**	-	-	-	**DNJ**	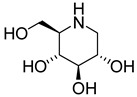	134.4 ± 2.1	33.1 ± 3.1
**26a**	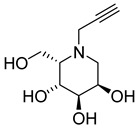	NI	1716 ± 12.8	**39a**	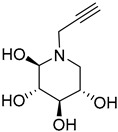	2527 ± 82.2	635.7 ± 8.5
**26b**	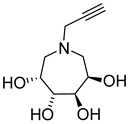	NI	NI	**39b**	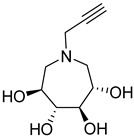	NI	3437 ± 70.6
**27a**	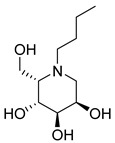	NI	109.7 ± 9.3	**40a**	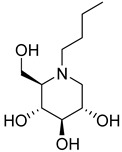	NI	172.8 ± 1.7
**27b**	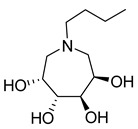	2031 ± 17.1	184.6 ± 2.6	**40b**	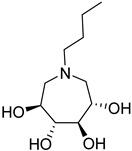	NI	80.0 ± 4.9
**28a**	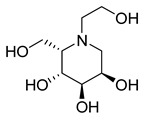	NI	NI	**41a**	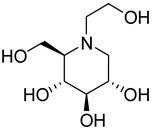	41.3 ± 10.1	4.0 ± 1.5
**28b**	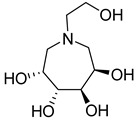	NI	NI	**41b**	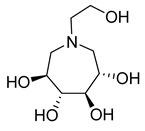	138.8 ± 1.2	4.0 ± 1.4
**29a**	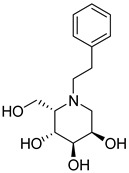	NI	NI	-	-	-	-
**29b**	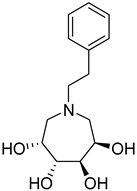	NI	NI	-	-	-	-

Enzyme inhibition: IC_50_ in µM, α-Glucosidase from *Saccharomyces cerevisiae* and β-Glucosidase from almonds. NI: no inhibition. DNJ; deoxynojirimycin

## Supplementary Materials

The following are available online at https://www.mdpi.com/1424-8247/12/3/108/s1, ^1^H, ^13^C and bidimensional NMR and ESI HRMS spectra of compounds 26-31a,b and 36-41a,b; Table: IC_50_ of final compounds and Dose-response curves obtained from Yeast α-Glucosidase and Almond β-Glucosidase assays.
