# Supplementary material for: Iminosugars: Effects of Stereochemistry, Ring Size, and N-Substituents on Glucosidase Activities
Source: Pharmaceuticals (Basel). 2019 Jul 12;12(3):108. doi: 10.3390/ph12030108 (PMC6789487; doi:10.3390/ph12030108)

Iminosugars: effects of stereochemistry, ring size and *N*-substituents on glucosidase activities

Luís O. B. Zamoner, Valquiria Aragão-Leoneti and Ivone Carvalho*

*School of Pharmaceutical Sciences of Ribeirão Preto, University of São Paulo, Av. do Café s/n, Monte Alegre,Ribeirão Preto,CEP14040-930, Brazil.*carronal@usp.br*

Table of contents

Content	Page
N-Propynyl-1,5-dideoxy-1,5-imino-L-gulitol (**26a**)	*2*
N-Propynyl-1,6-dideoxy-1,6-imino-D-mannitol (**26b**)	*6*
N-Butyl-1,5-dideoxy-1,5-imino-L-gulitol (**27a**)	*10*
N-Butyl-1,6-dideoxy-1,6-imino-D-mannitol (**27b**)	*13*
N-Hydroxyethyl-1,5-dideoxy-1,5-imino-L-gulitol (**28a**)	*16*
N-Hydroxyethyl-1,6-imino-D-mannitol (**28b**)	*19*
N-Phenethyl-1,5-dideoxy-1,5-imino-L-gulitol (**29a**)	*22*
N-Phenetyl-1,6-dideoxy-1,6-imino-D-mannitol (**29b**)	*23*
N-Propynyl-2,3,4,6-tetra-*O*-acetyl-1,5-dideoxy-1,5-imino-L-gulitol (**30a**)	*26*
N-Propynyl-2,3,4,6-tetra-*O*-acetyl-1,6-dideoxy-1,6-imino-D-mannitol (**30b**)	*29*
N-Acetoxyethyl-2,3,4,6-tetra-*O*-acetyl-1,5-dideoxy-1,5-imino-L-gulitol (**31a**)	*32*
N-Acetoxyethyl-2,3,4,6-tetra-*O*-acetyl-1,6-dideoxy-1,6-imino-D-mannitol (**31b**)	*35*
N-Propynyl-2,3,4,6-tetra-*O*-acetyl-1,5-dideoxy-1,5-imino-D-glucitol (**36a**)	*38*
N-Propynyl-2,3,4,6-tetra-*O*-acetyl-1,6-dideoxy-1,6-imino-L-iditol (**36b**)	*41*
N-Butyl-2,3,4,6-tetra-*O*-acetyl-1,5-dideoxy-1,5-imino-D-glucitol (**37a**)	*44*
N-Butyl-2,3,4,6-tetra-*O*-acetyl-1,6-dideoxy-1,6-imino-L-iditol (**37b**)	*47*
N-Hydroxyethyl-2,3,4,6-tetra-*O*-acetyl-1,5-dideoxy-1,5-imino-D-glucitol (**38a**)	*50*
N-Hydroxyethyl-2,3,4,6-tetra-*O*-acetyl-1,6-dideoxy-1,6-imino-L-iditol (**38b**)	*53*
N-Propynyl-1,5-dideoxy-1,5-imino-D-glucitol (**39a**)	*56*
N-Propynyl-1,6-dideoxy-1,6-imino-L-iditol (**39b**)	*57*
N-Butyl-1,5-dideoxy-1,5-imino-D-glucitol (**40a**)	*58*
N-Butyl-1,6-dideoxy-1,6-imino-L-iditol (**40b**)	*61*
N-Hydroxyethyl-1,5-dideoxy-1,5-imino-D-glucitol (**41a**)	*64*
N-Hydroxyethyl-1,6-dideoxy-1,6-imino-L-iditol (**41b**)	*67*
IC50 in α and β-Glucosidase	*70*
Graphics of α-Glucosidase Yeast assays	*72*
Graphics of β-Glucosidase Almond assays	*74*

N-Propynyl-1,5-dideoxy-1,5-imino-L-gulitol (26a):

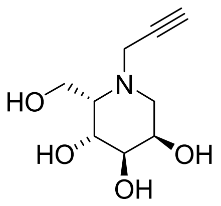

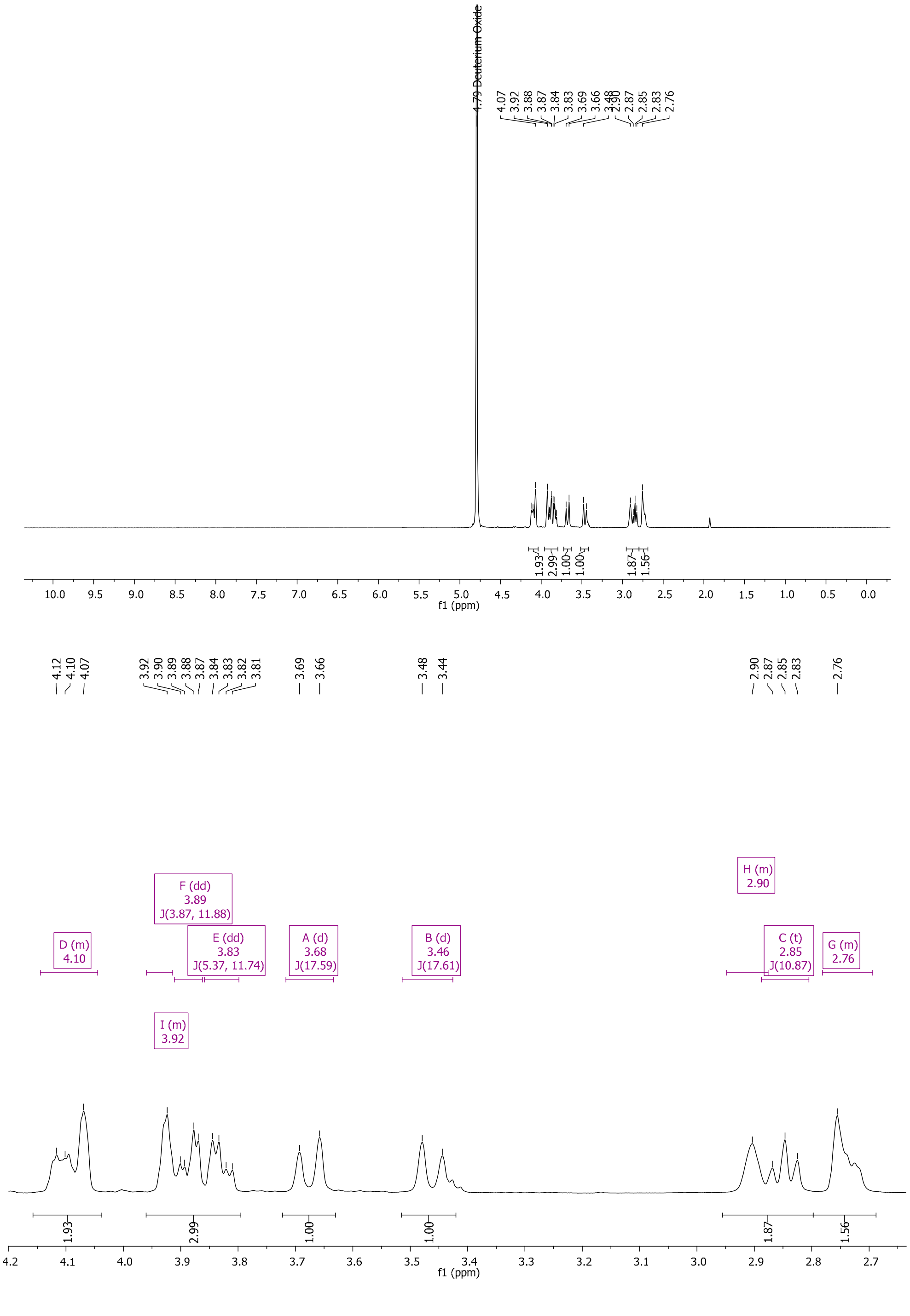

^{1}H NMR (500 MHz, D_2O)

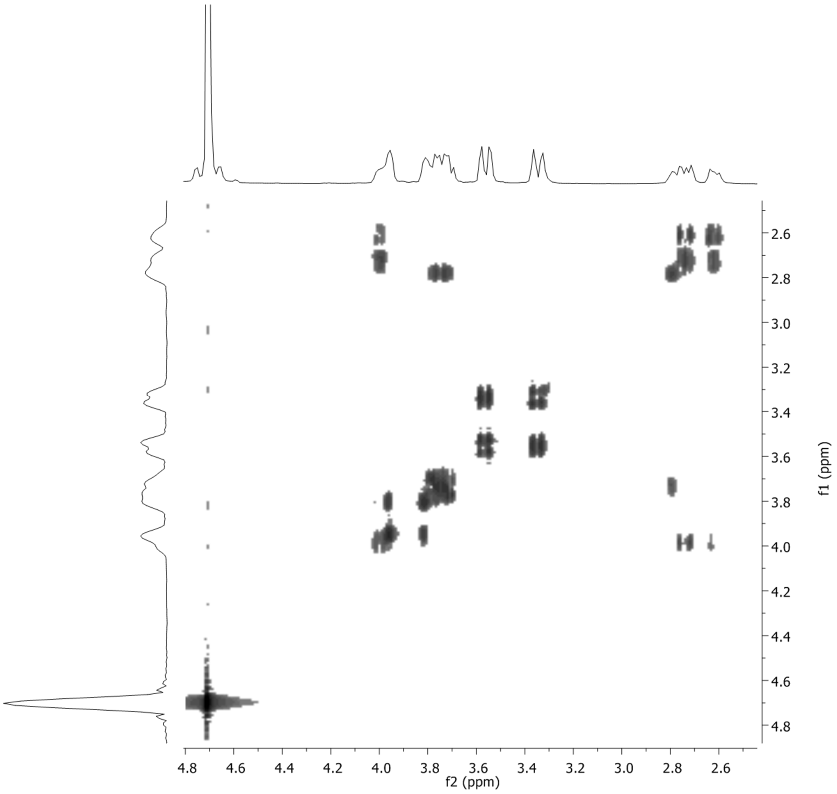

COSY NMR

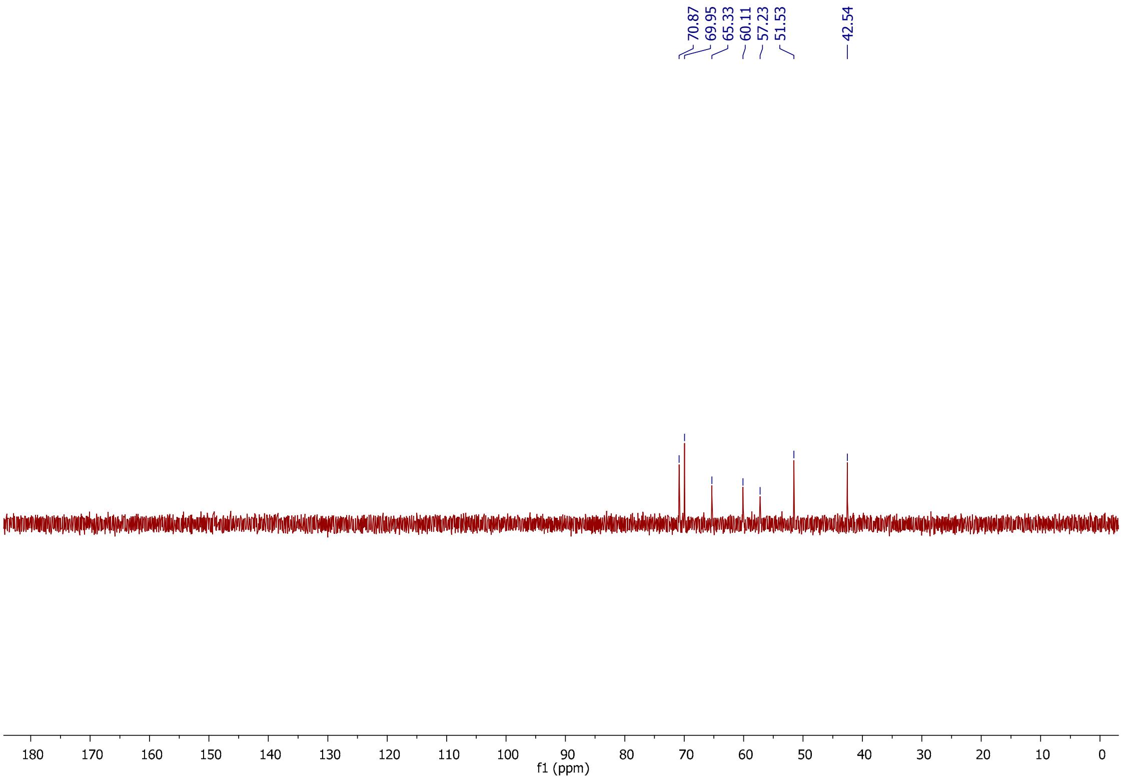

^{13}C NMR (125 MHz, D_2O)

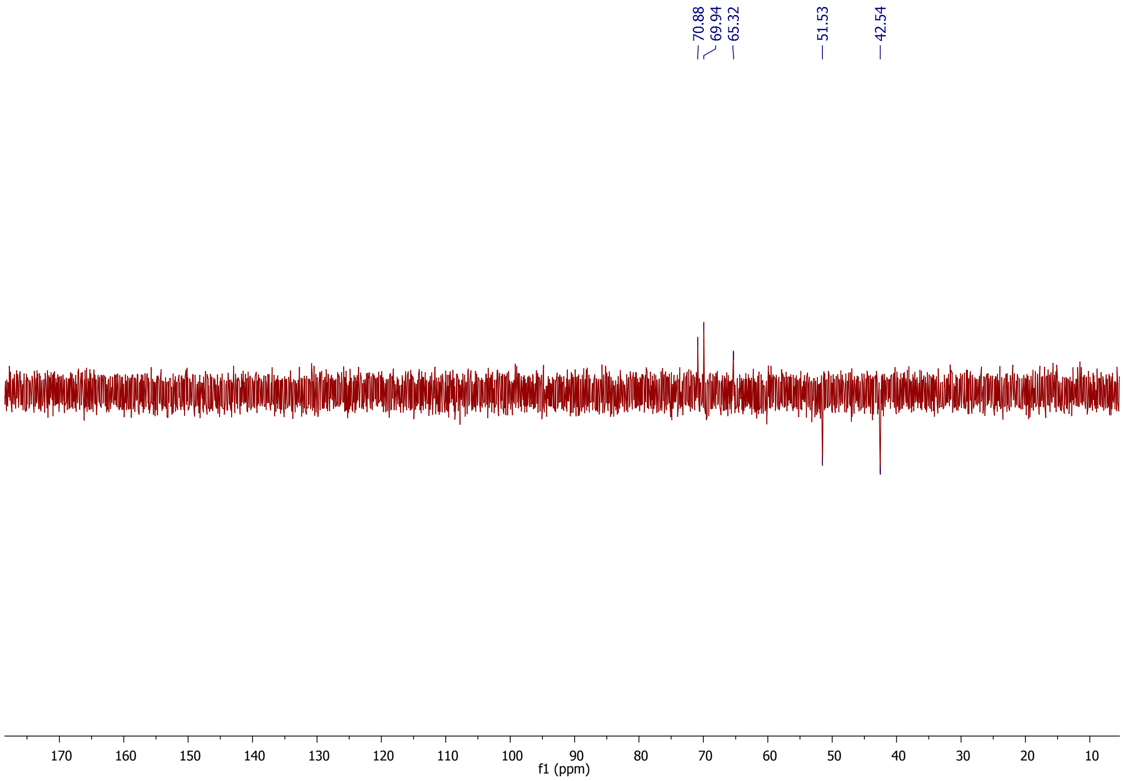

^{13}C NMR DEPT (125 MHz, D_2O)

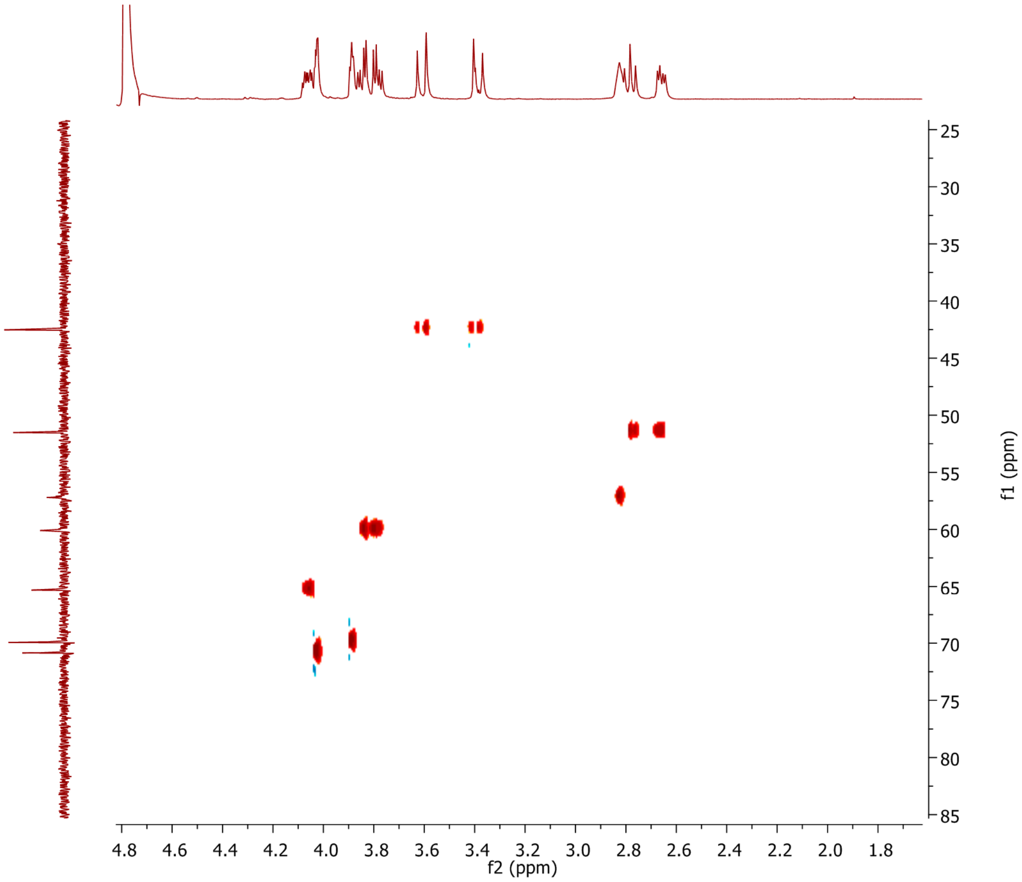

HMQC NMR

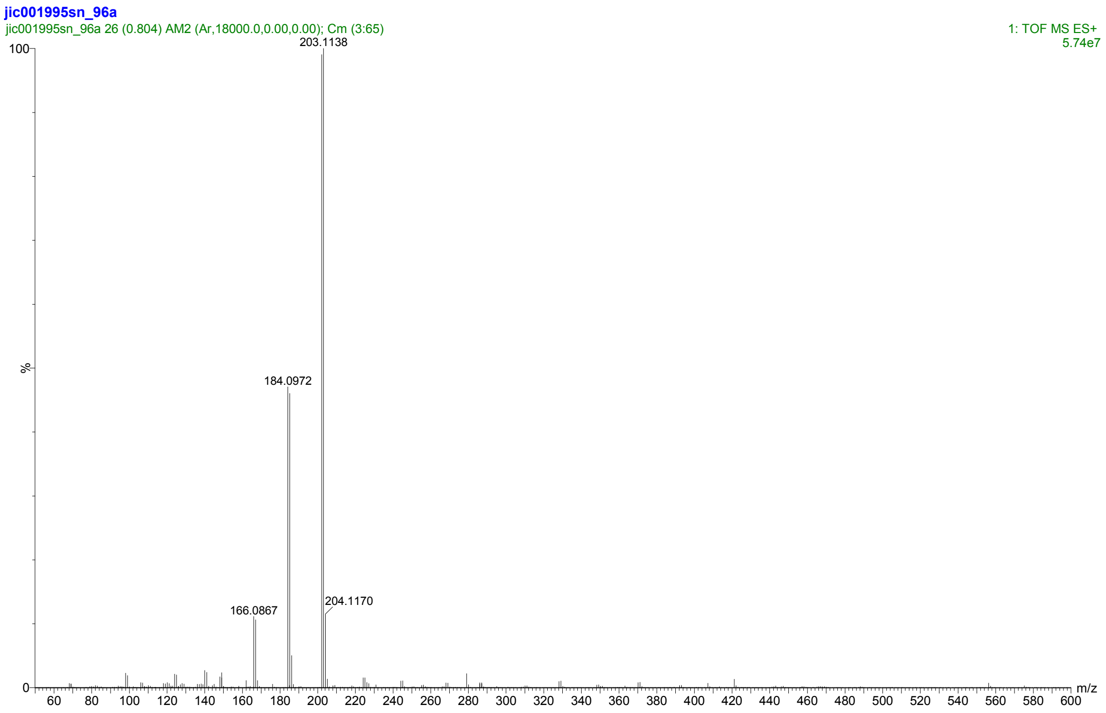

ESI HRMS: $[M+H]^+$ calcd for $C_9H_{15}NO_4$ 202.1074; found 202.1076.

***N*-Propynyl-1,6-dideoxy-1,6-imino-D-mannitol (26b):**

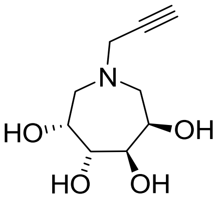

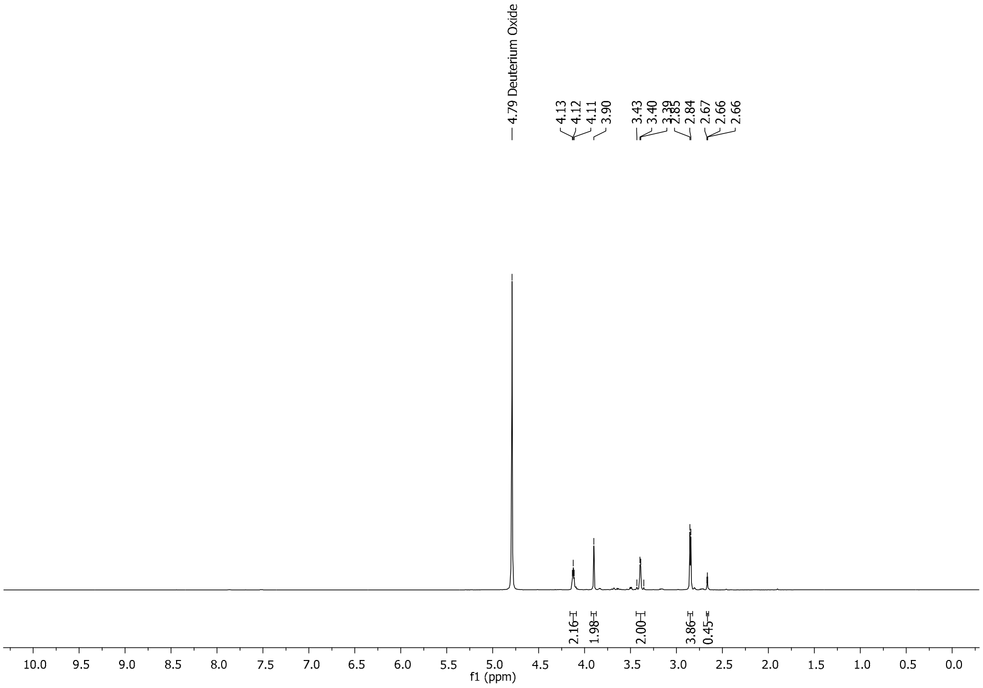

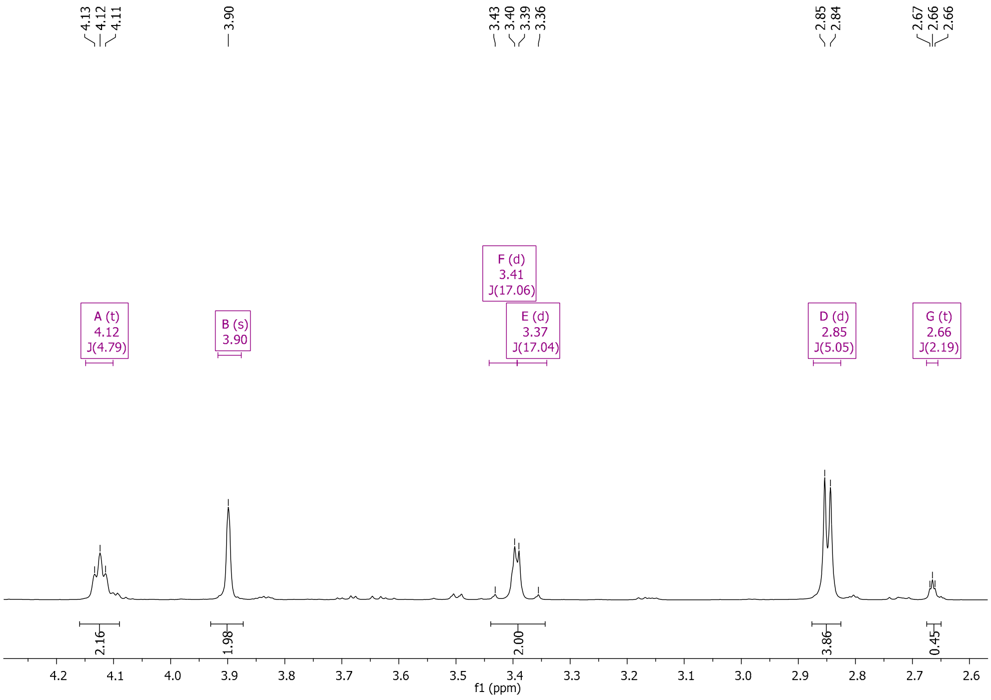

^{1}H NMR (500 MHz, D_2O)

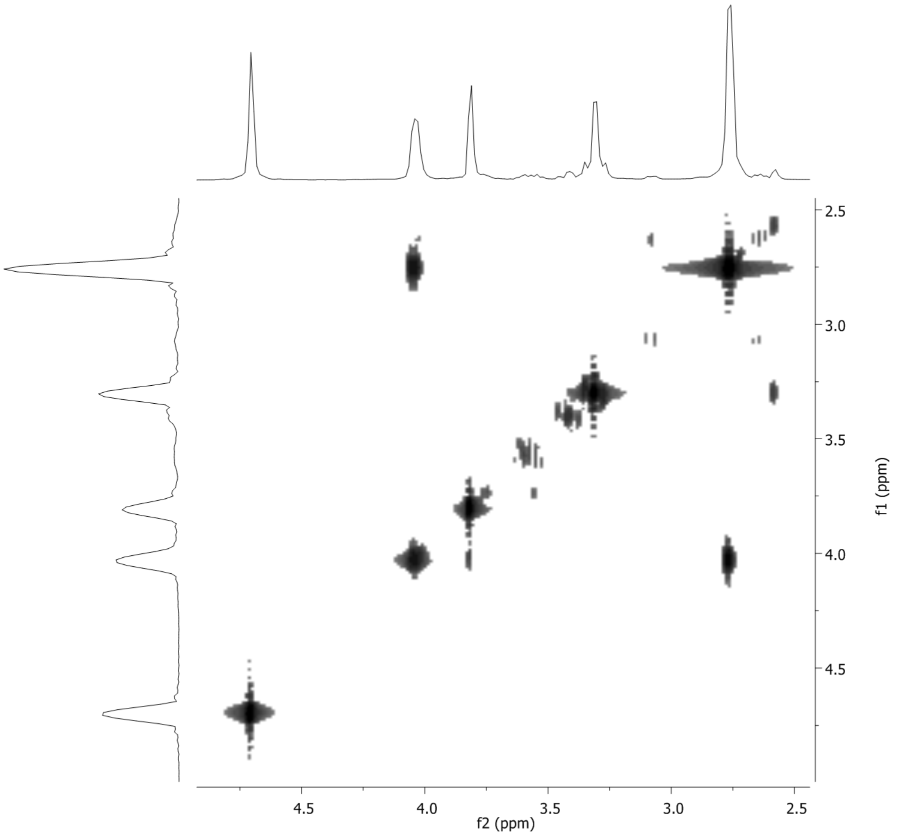

COSY NMR

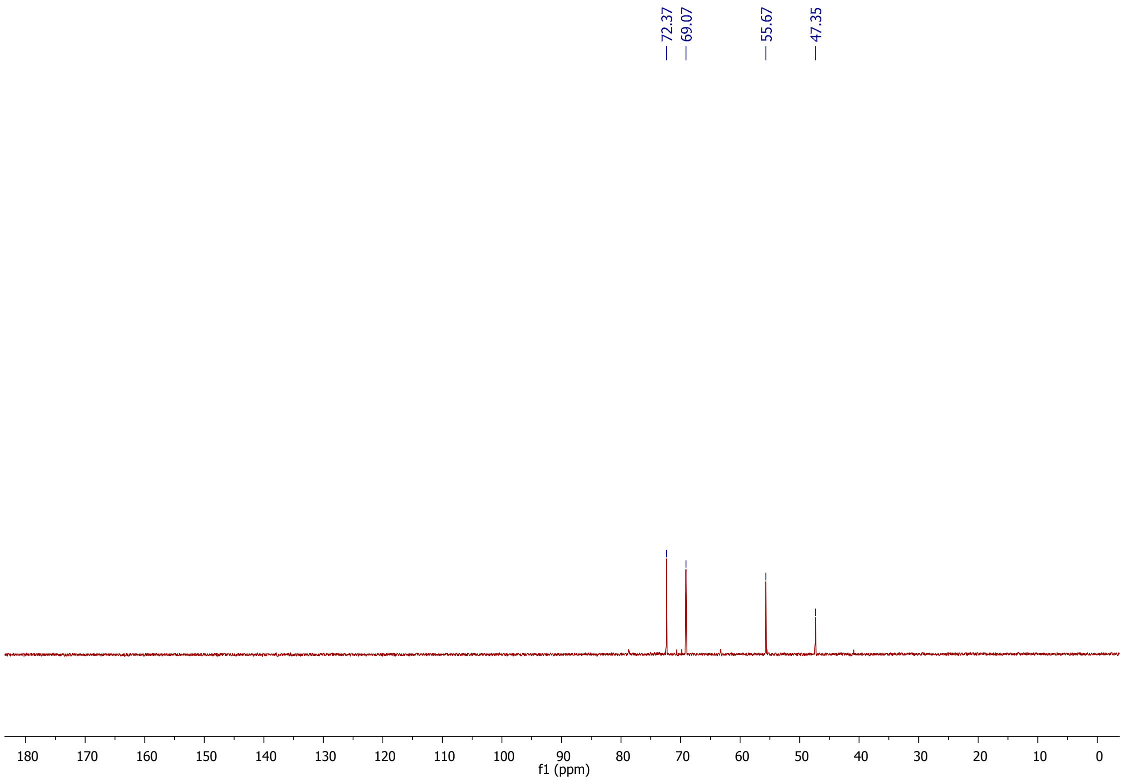

^{13}C NMR (100 MHz, D_2O)

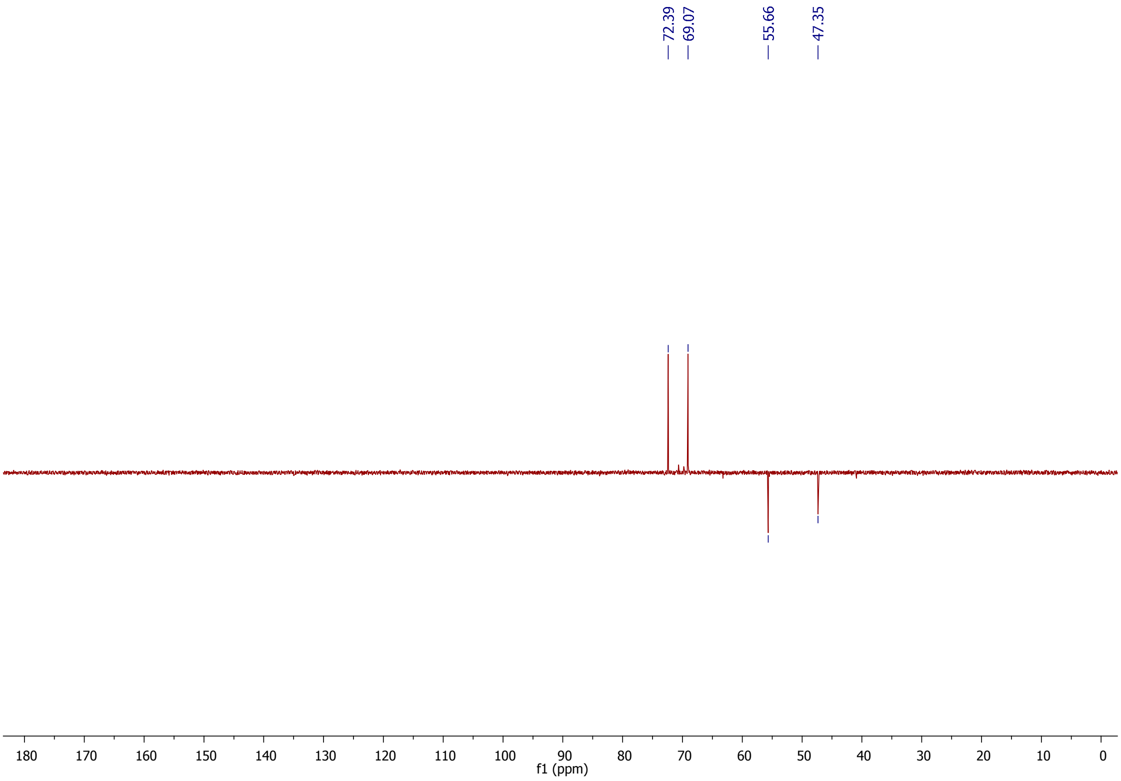

^{13}C NMR DEPT (100 MHz, D_2O)

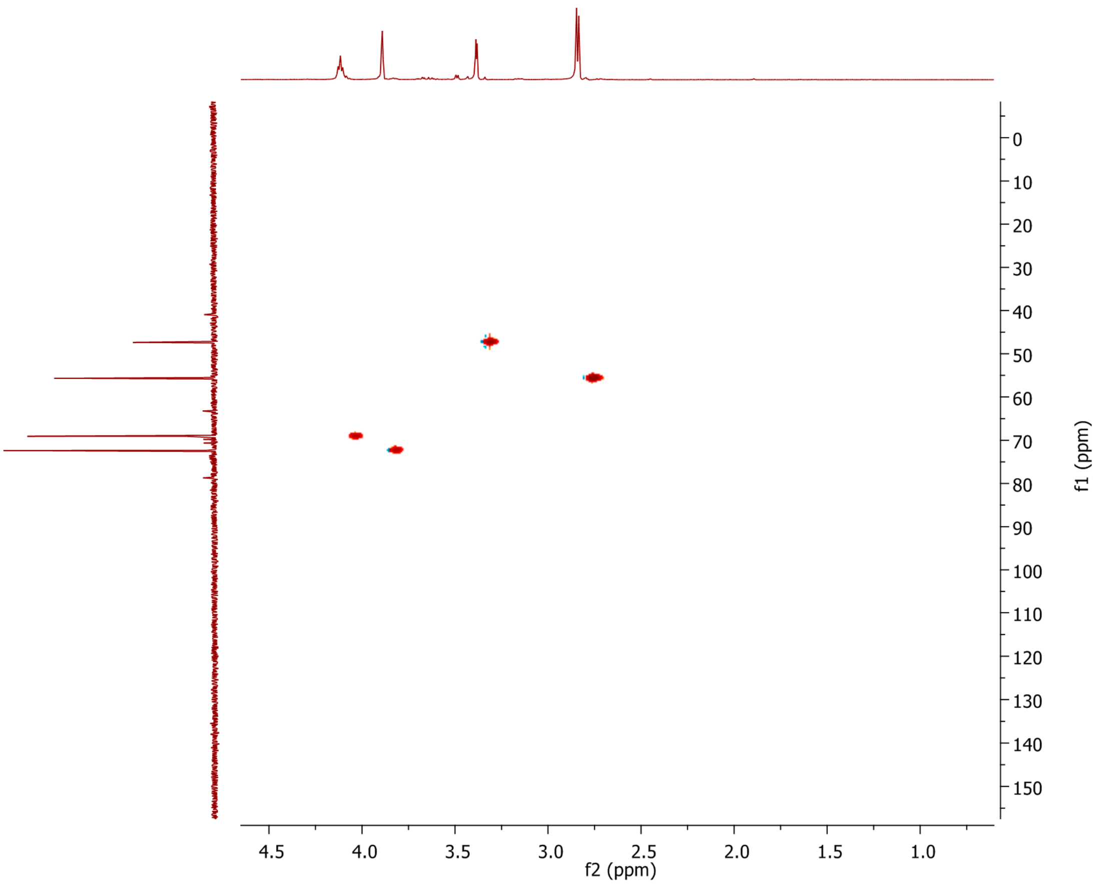

HMQC NMR

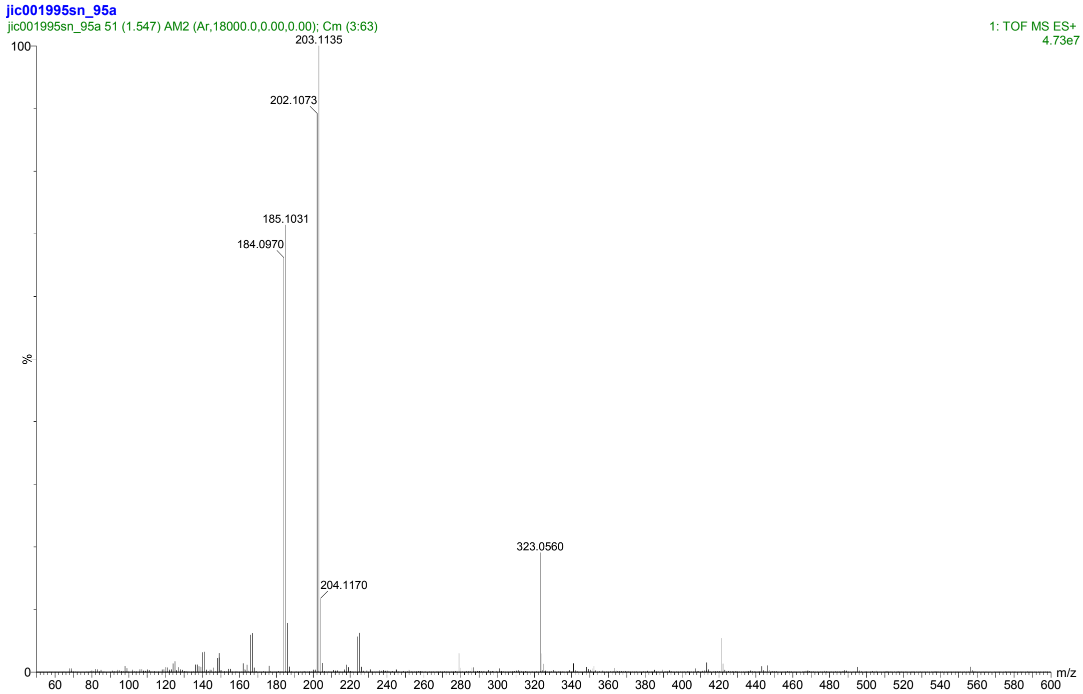

ESI HRMS: $[M+H]^+$ calcd for $C_9H_{15}NO_4$ 202.1074; found 202.1073.

N-Butyl-1,5-dideoxy-1,5-imino-L-gulitol (27a):

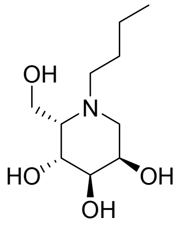

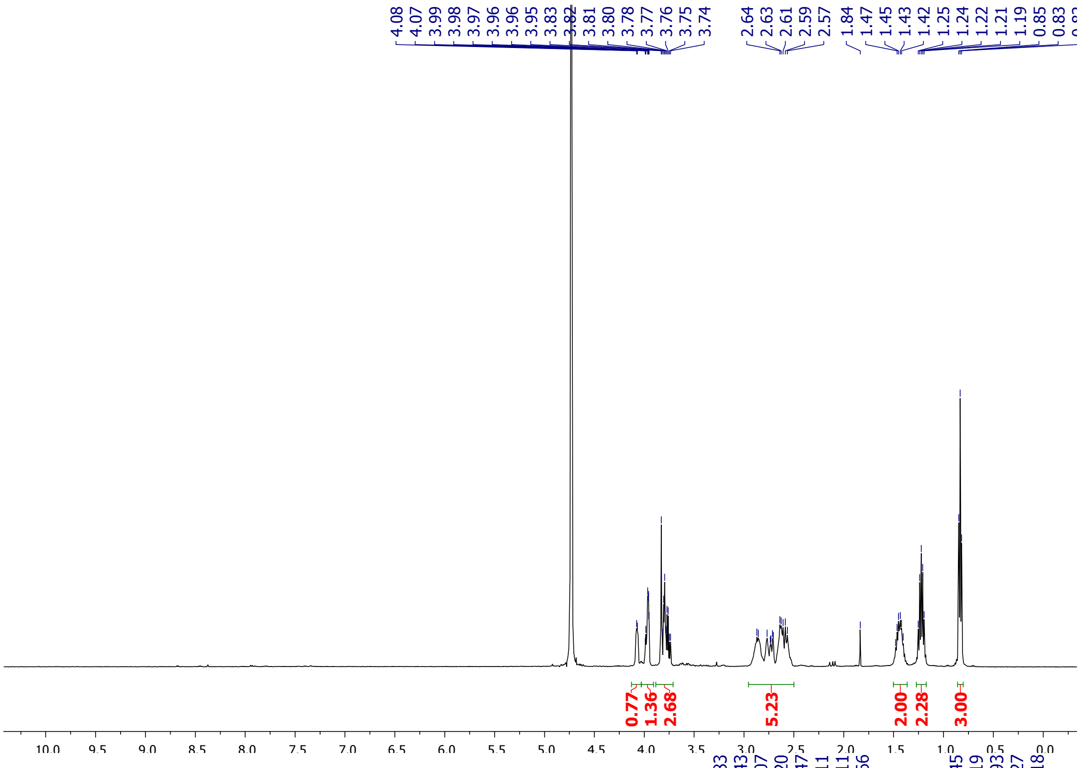

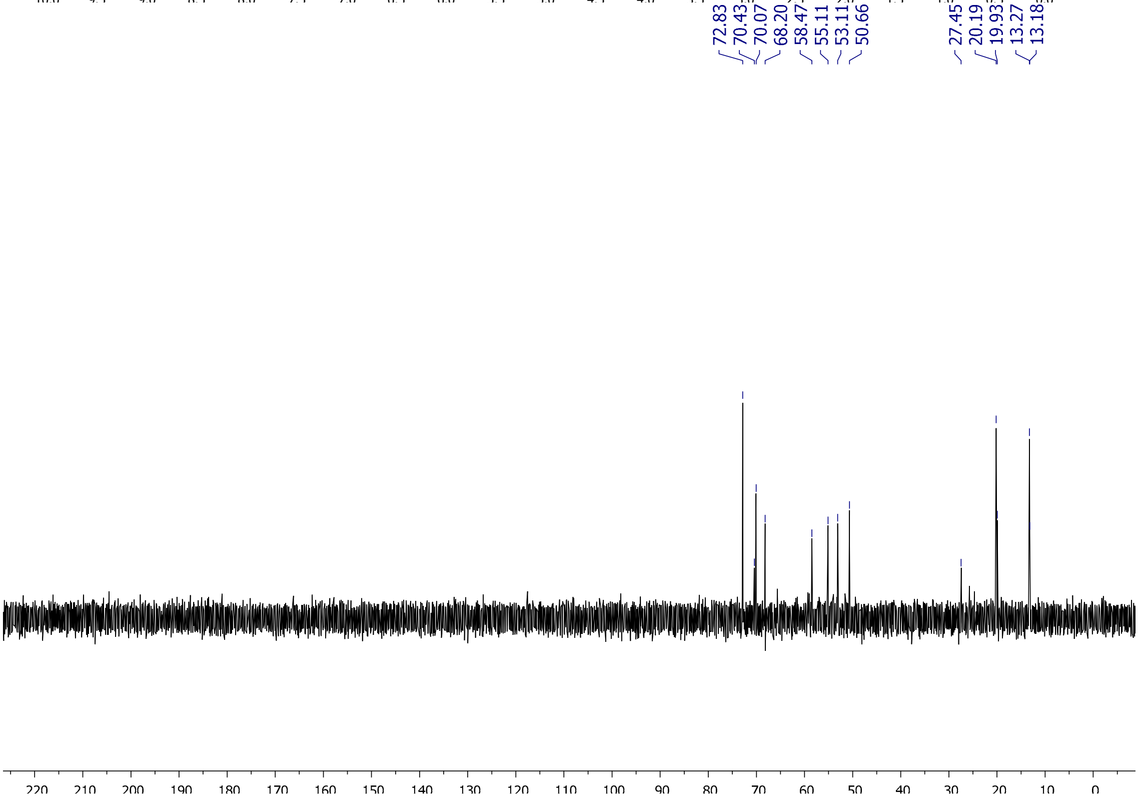

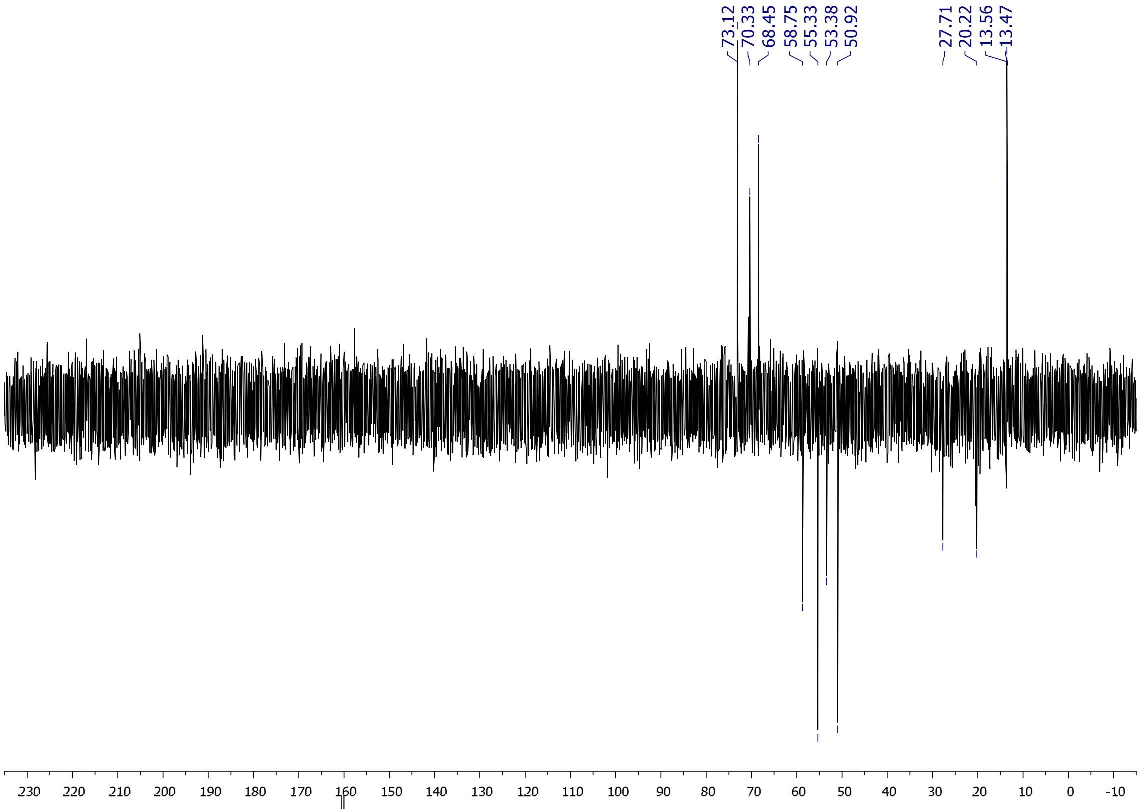
73.12
70.33
68.45
58.75
55.33
53.38
50.92
27.71
20.22
13.56
13.47
230 220 210 200 190 180 170 160 150 140 130 120 110 100 90 80 70 60 50 40 30 20 10 0 -10

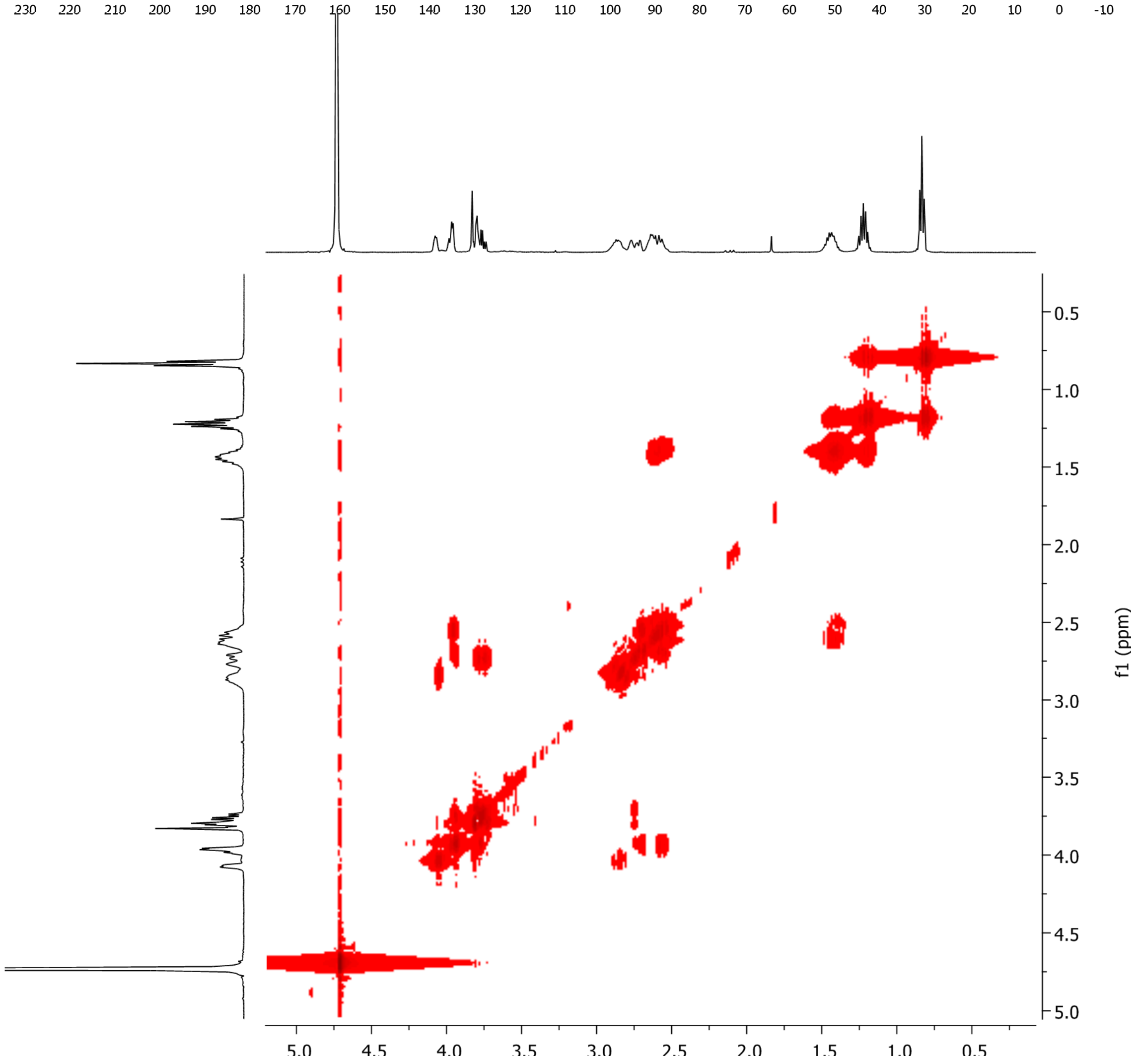
0.5
1.0
1.5
2.0
2.5
3.0
3.5
4.0
4.5
5.0
f1 (ppm)
5.0 4.5 4.0 3.5 3.0 2.5 2.0 1.5 1.0 0.5

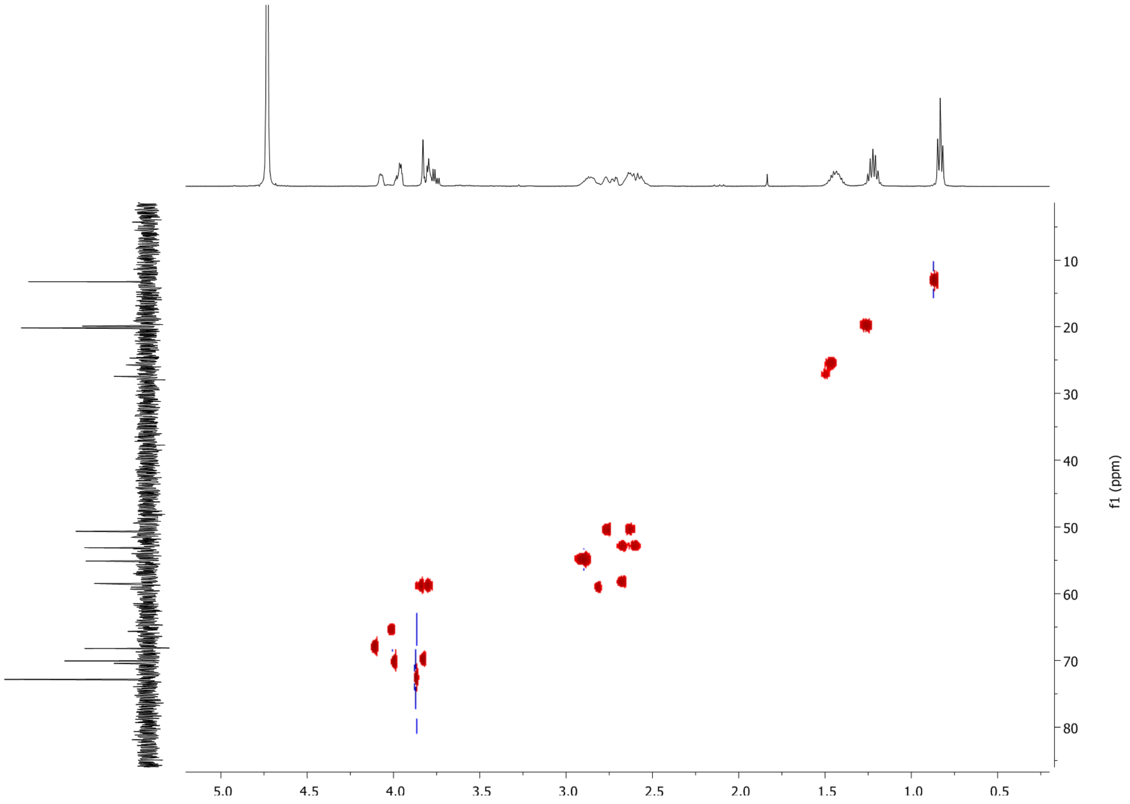

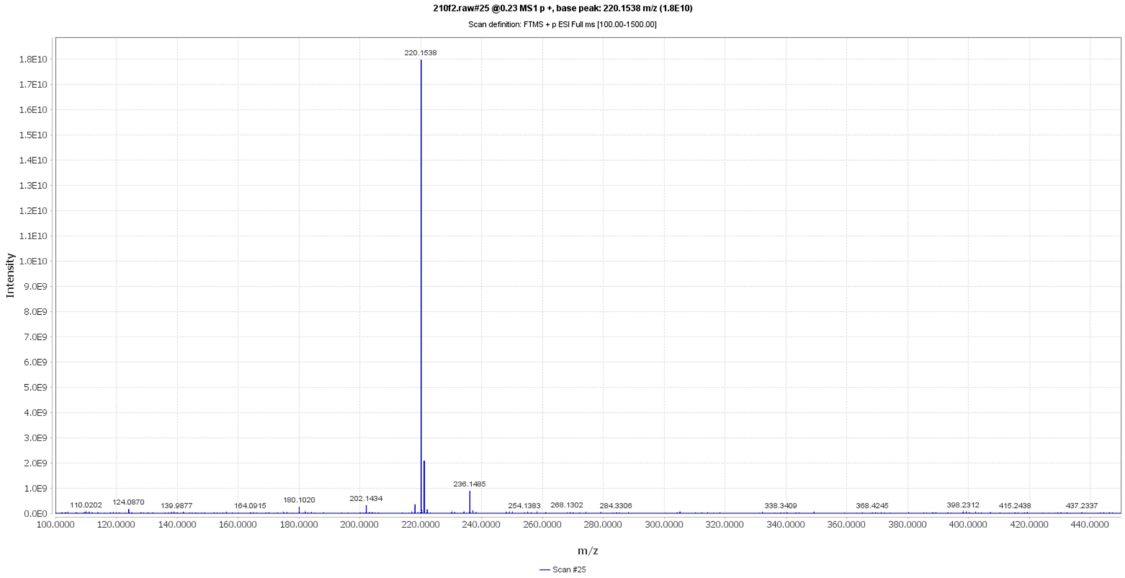

ESI HRMS: $[M+H]^+$ calcd for $C_{10}H_{22}NO_4$ 220.1544; found 220.1538.

_N_-Butyl-1,6-dideoxy-1,6-imino-D-mannitol (27b):

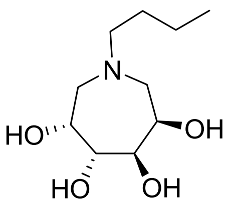

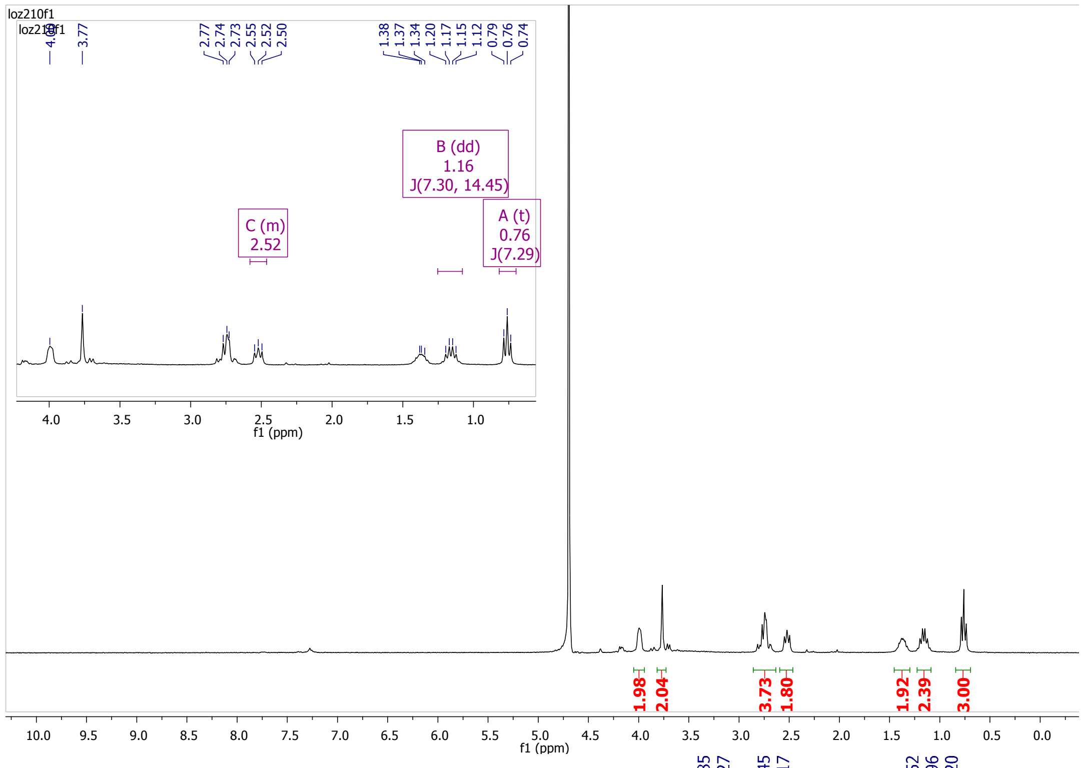

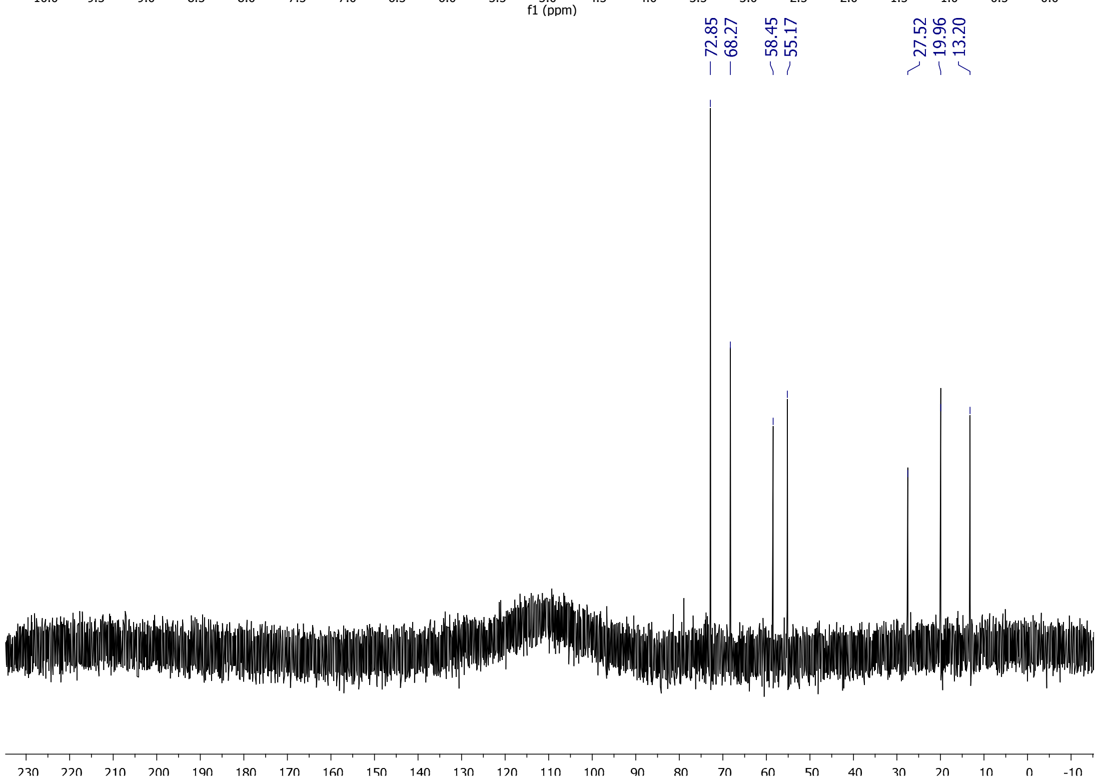

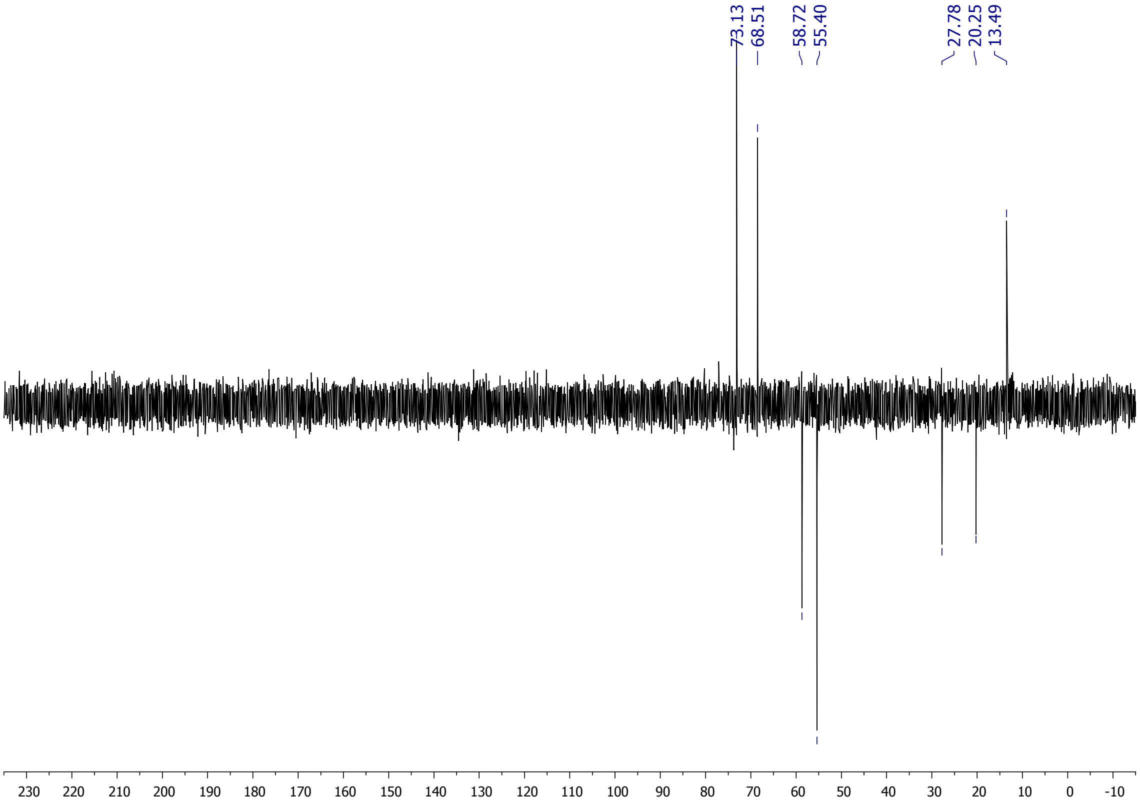
73.13
68.51
58.72
55.40
27.78
20.25
13.49
230 220 210 200 190 180 170 160 150 140 130 120 110 100 90 80 70 60 50 40 30 20 10 0 -10

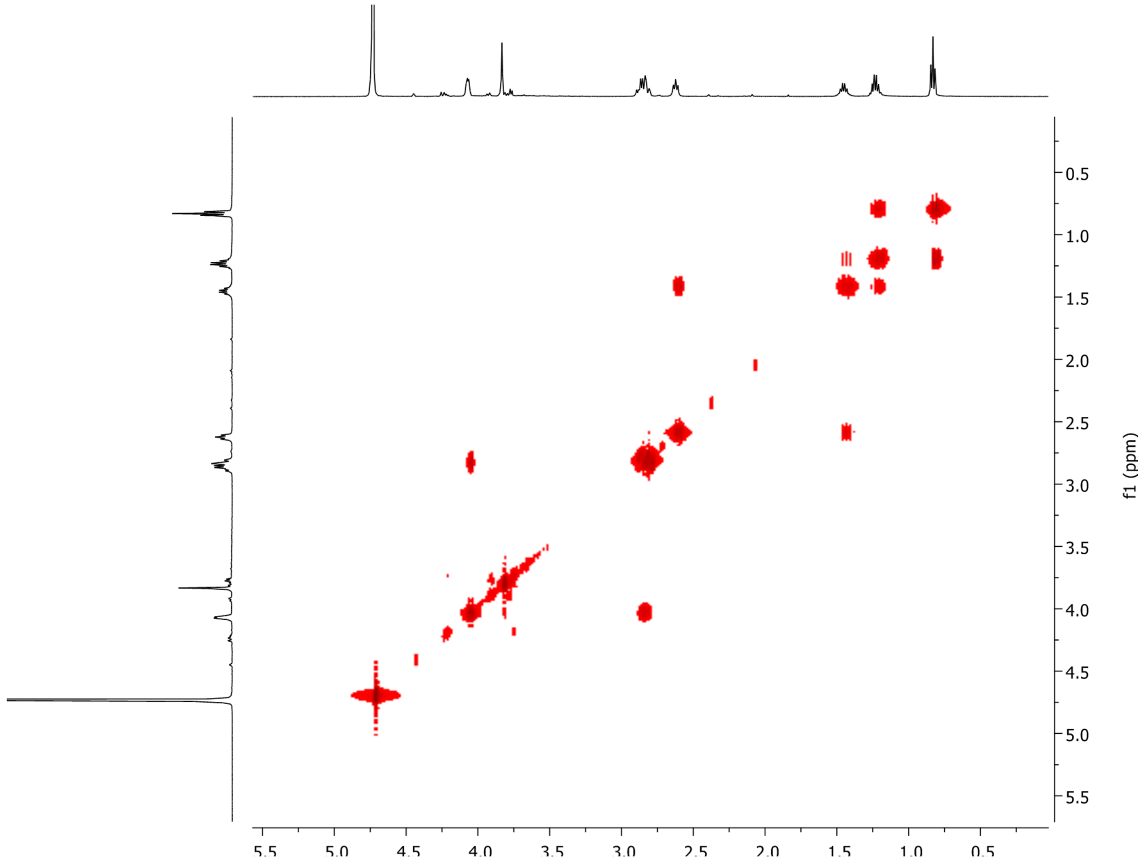
0.5
1.0
1.5
2.0
2.5
3.0
3.5
4.0
4.5
5.0
5.5
f1 (ppm)
5.5 5.0 4.5 4.0 3.5 3.0 2.5 2.0 1.5 1.0 0.5

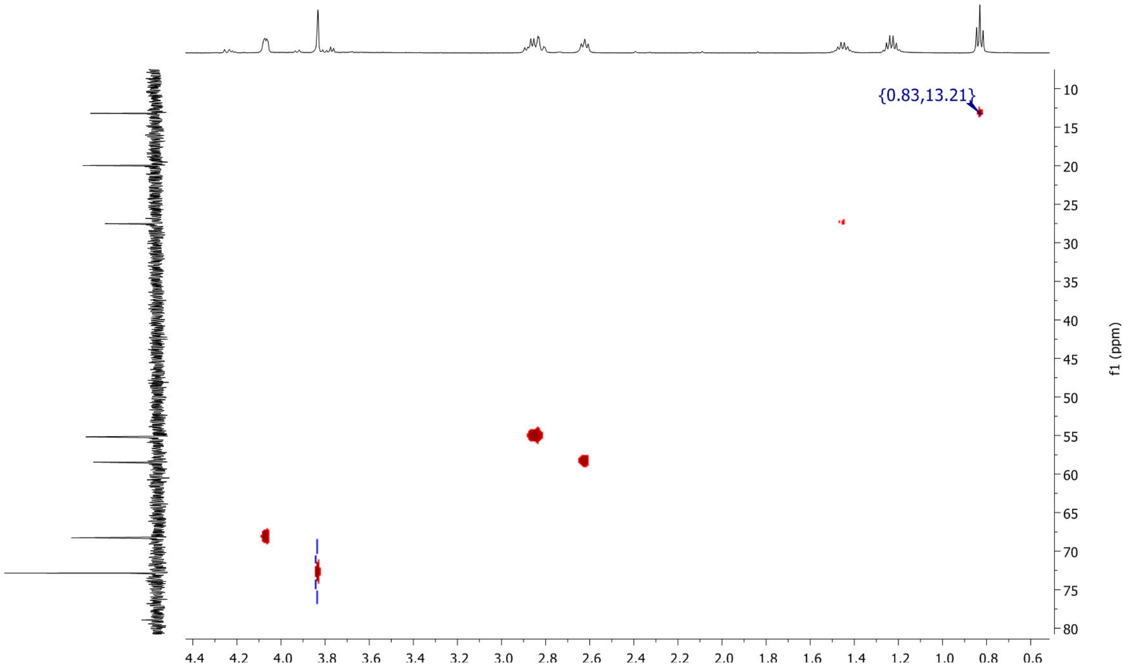

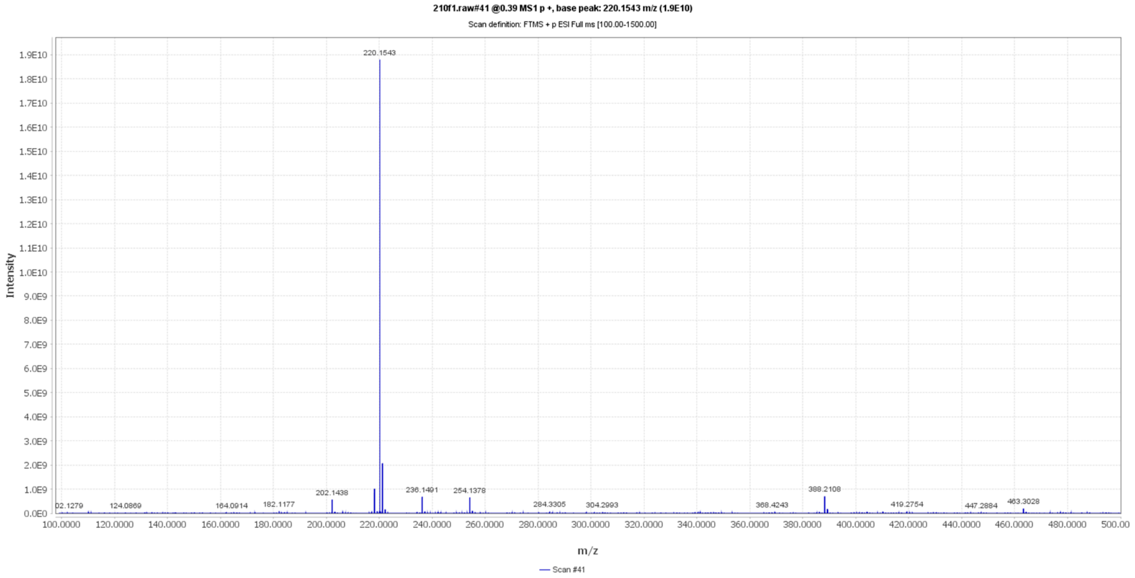

ESI HRMS: $[M+H]^+$ calcd for $C_{10}H_{22}NO_4$ 220.1544; found 220.1543

N-Hydroxyethyl-1,5-dideoxy-1,5-imino-L-gulitol (28a):

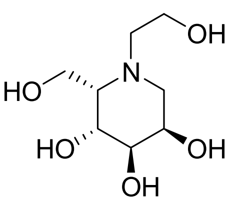

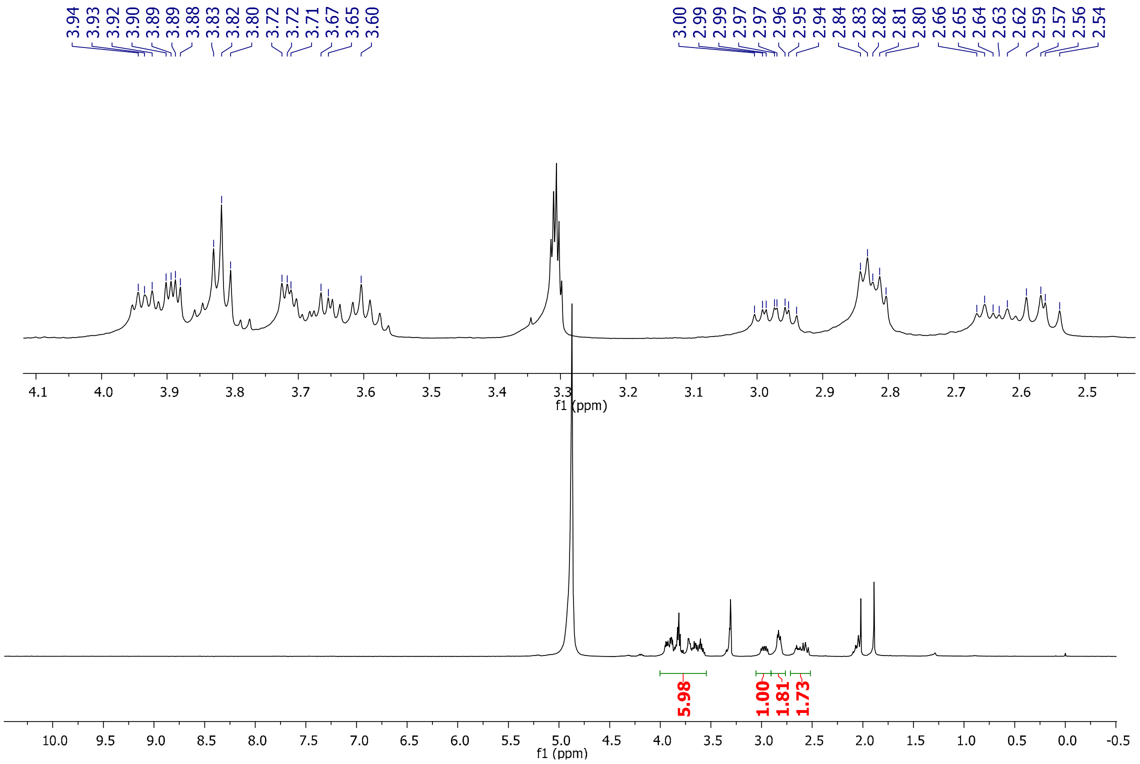

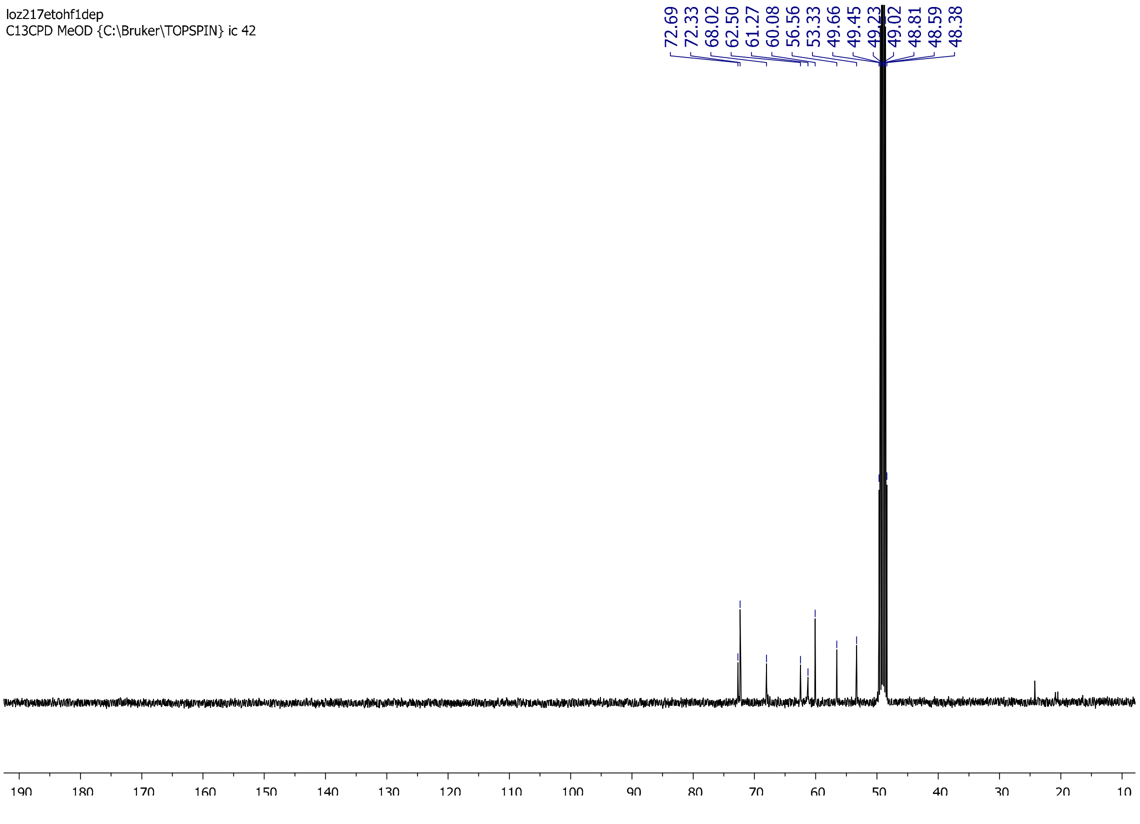

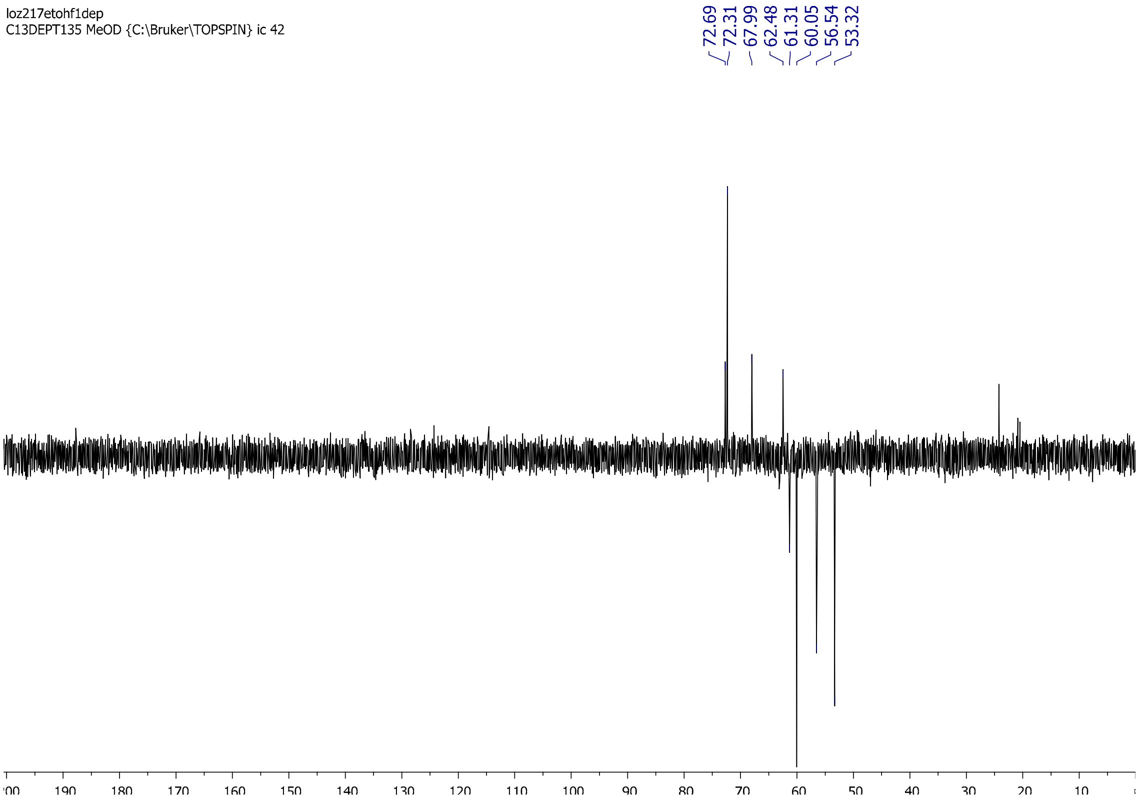
loz217etohf1dep
C13DEPT135 MeOD {C:\Bruker\TOPSPIN} ic 42
72.69
72.31
67.99
62.48
61.31
60.05
56.54
53.32
190
180
170
160
150
140
130
120
110
100
90
80
70
60
50
40
30
20
10

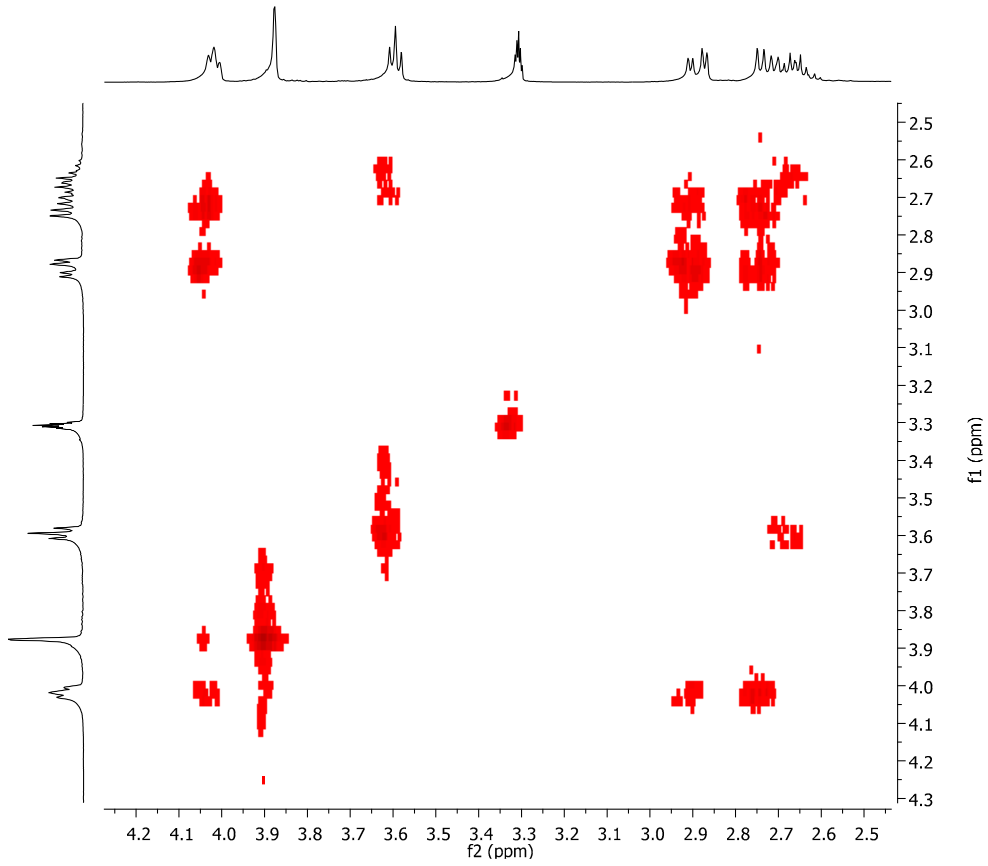
f2 (ppm)
f1 (ppm)
4.2
4.1
4.0
3.9
3.8
3.7
3.6
3.5
3.4
3.3
3.2
3.1
3.0
2.9
2.8
2.7
2.6
2.5
4.3

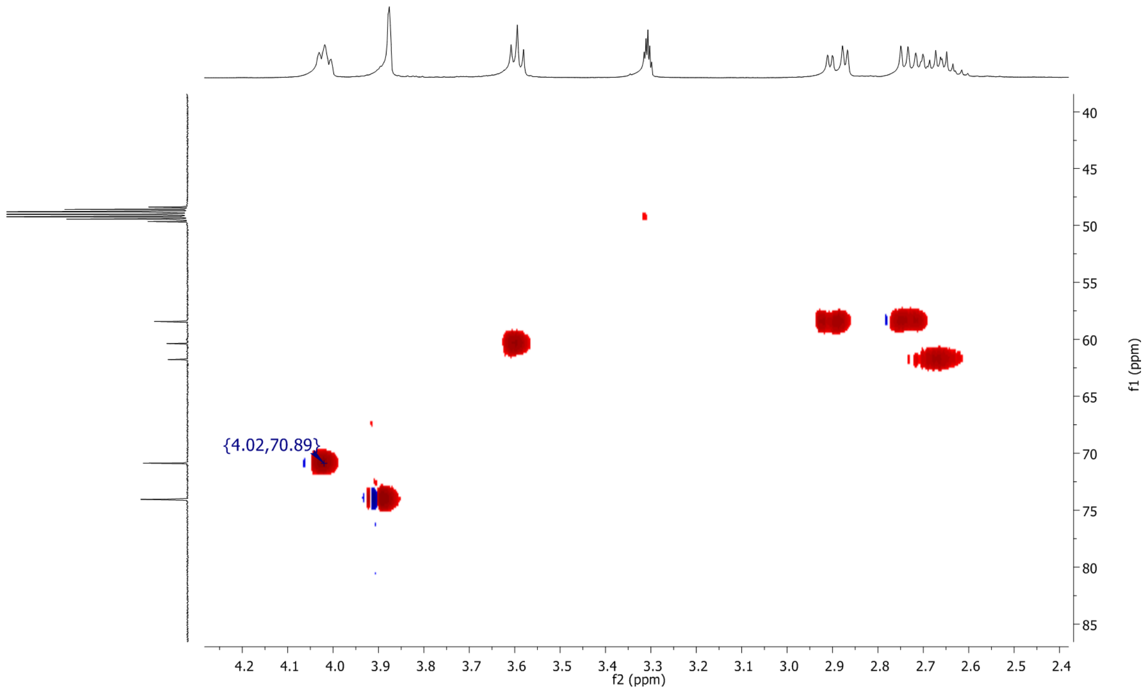

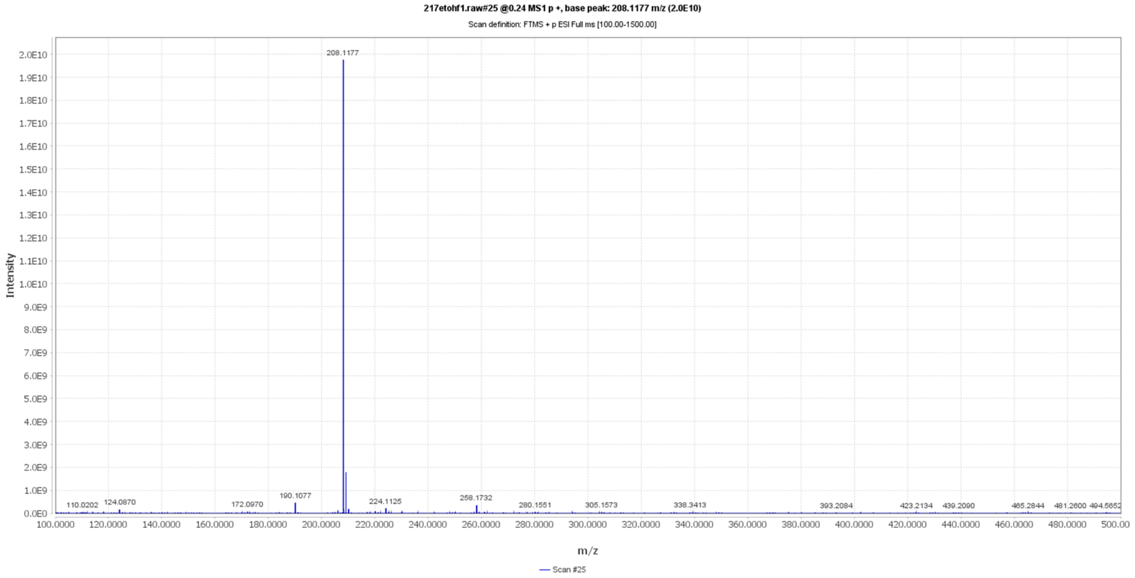

ESI HRMS: $[M+H]^+$ calcd for $C_8H_{18}NO_5$ 208.1180; found 208.1177

***N*-Hydroxyethyl-1,6-imino-D-mannitol (28b):**

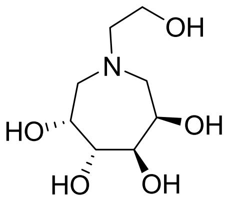

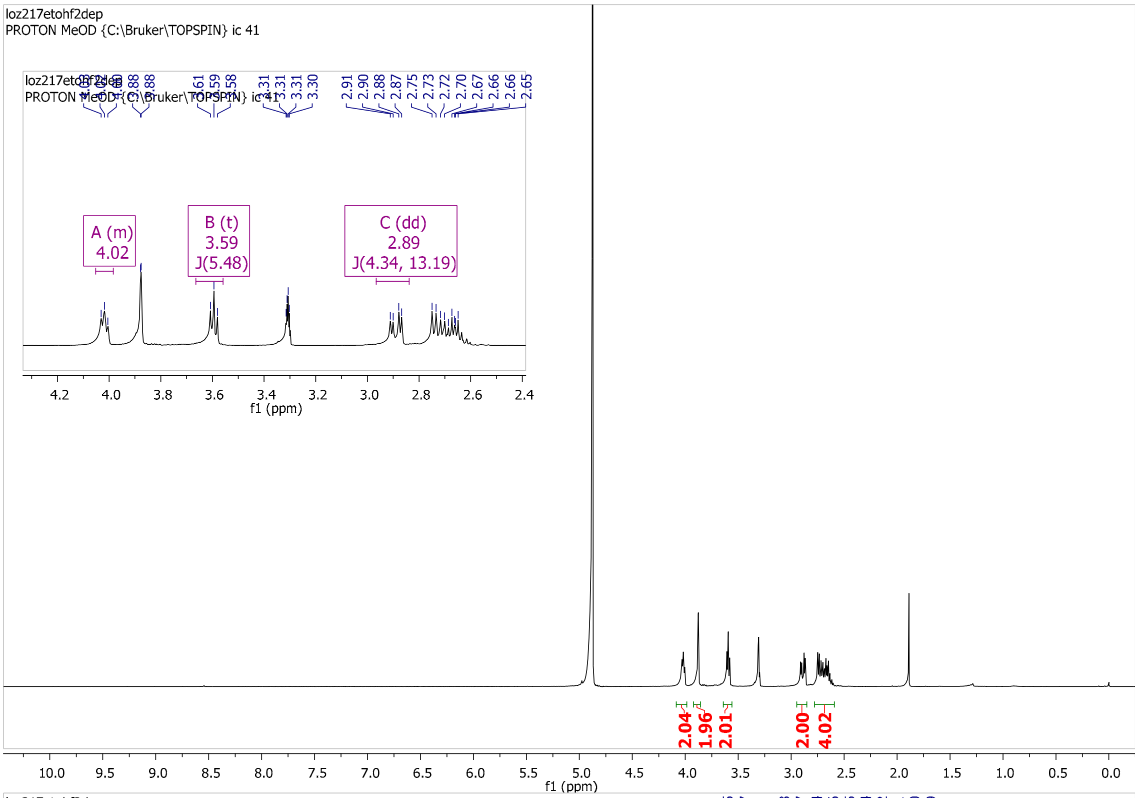

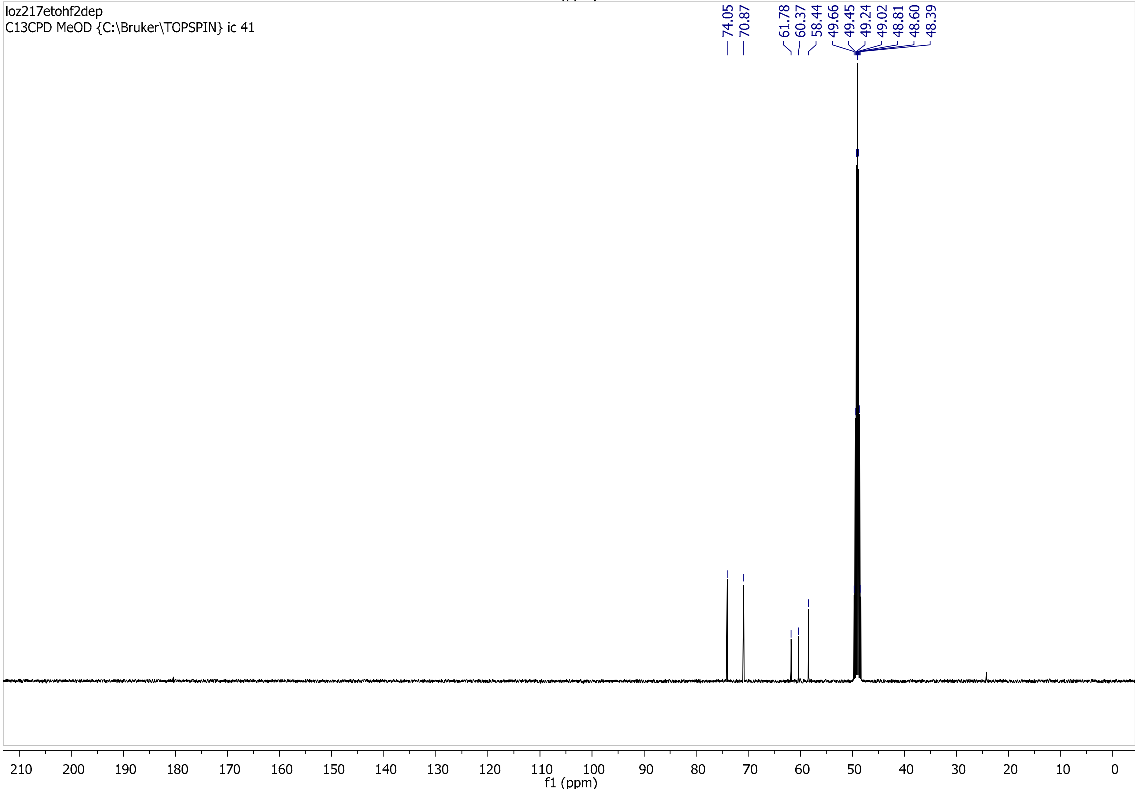

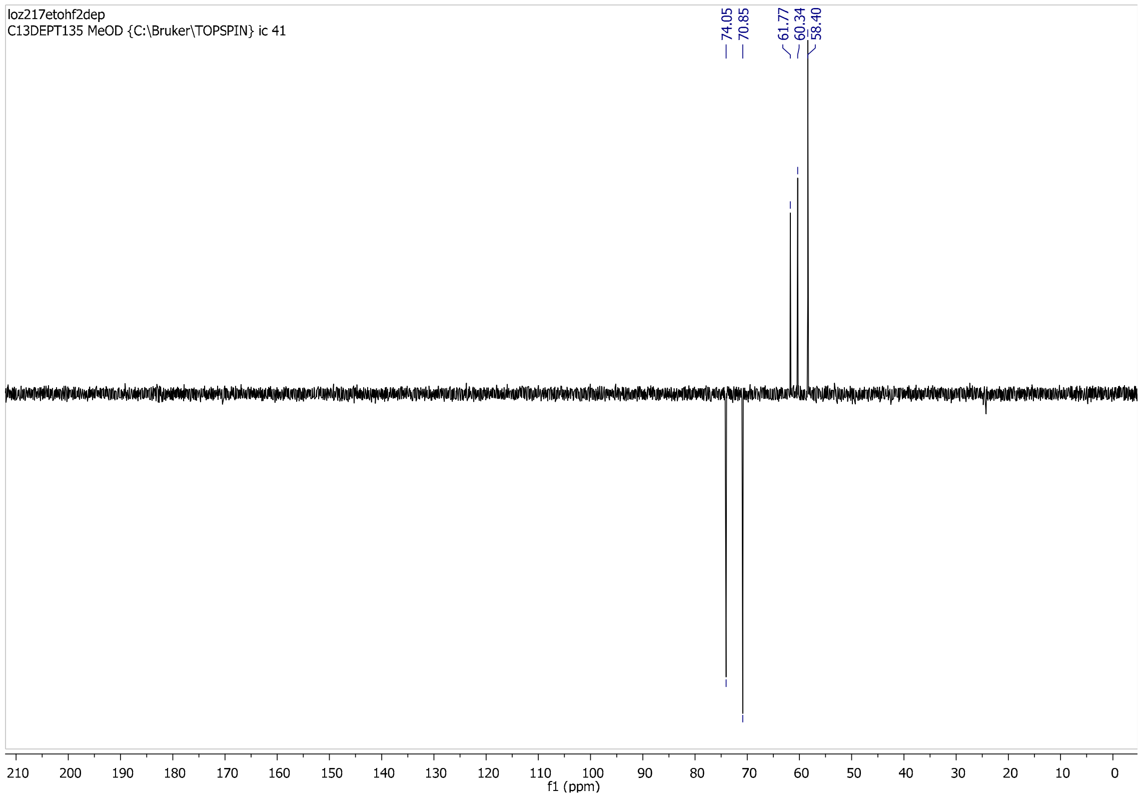
loz217etohf2dep
C13DEPT135 MeOD {C:\Bruker\TOPSPIN} ic 41
74.05
70.85
61.77
60.34
58.40
f1 (ppm)

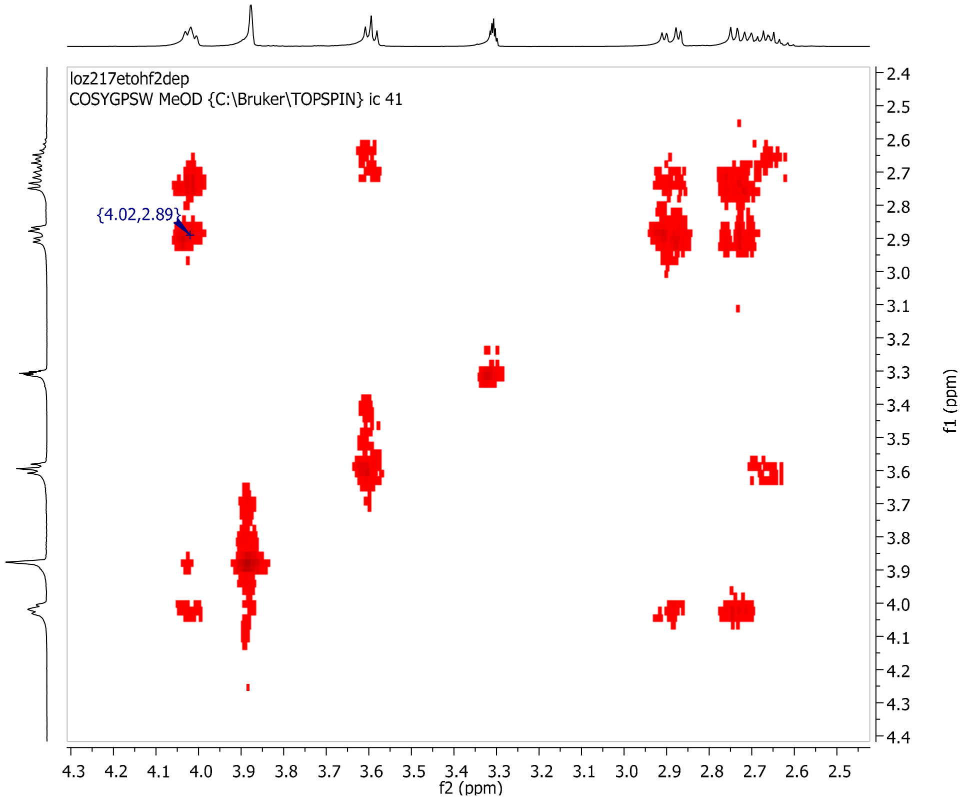
loz217etohf2dep
COSYGPSW MeOD {C:\Bruker\TOPSPIN} ic 41
{4.02,2.89}
f2 (ppm)
f1 (ppm)

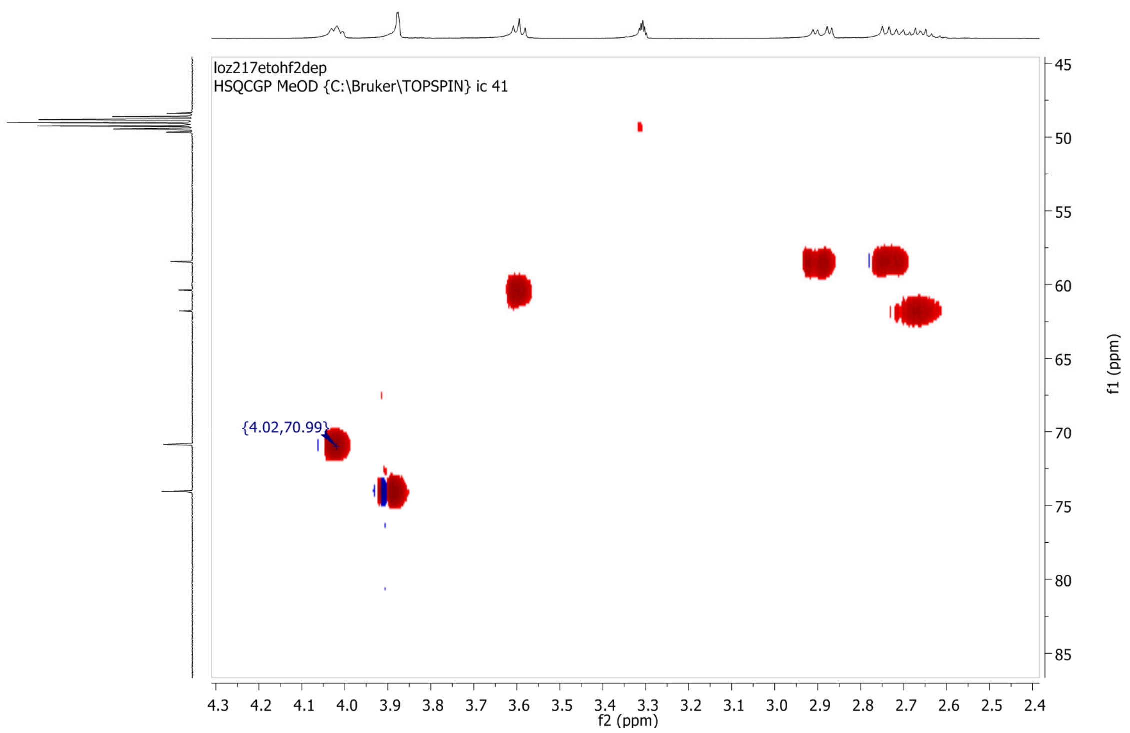

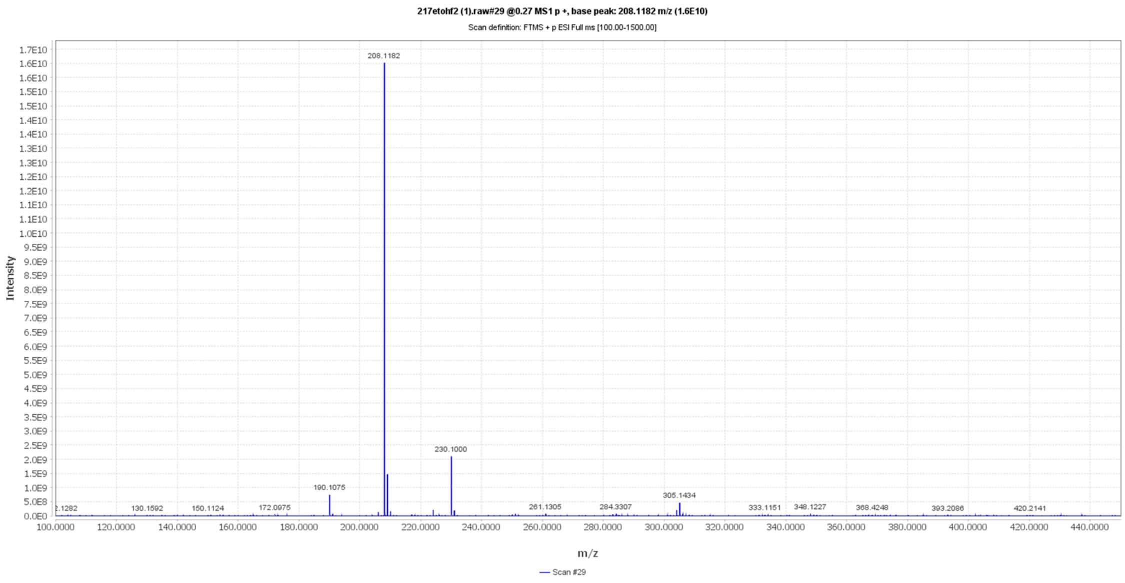

ESI HRMS: $[M+H]^+$ calcd for $C_8H_{18}NO_5$ 208.1180; found 208.1182

N-Phenethyl-1,5-dideoxy-1,5-imino-L-gulitol (29a):

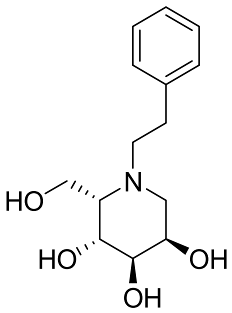

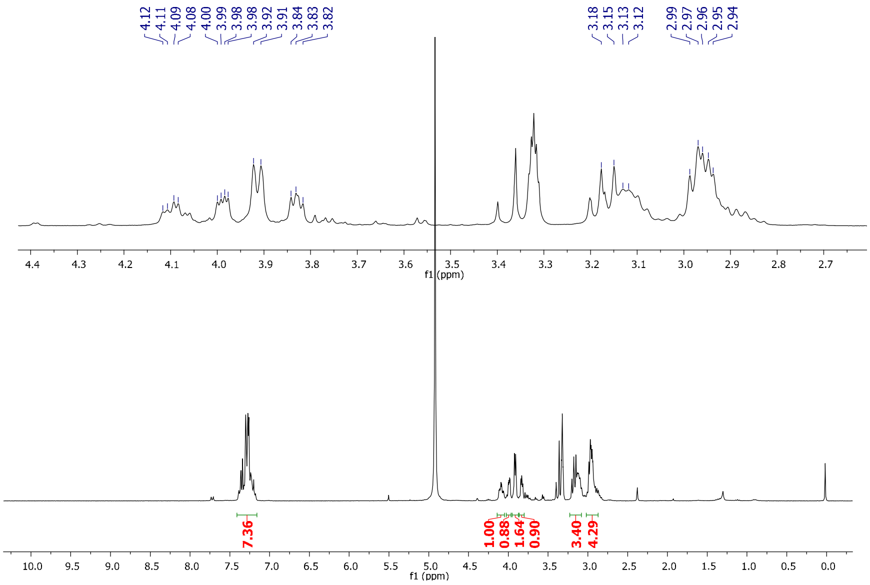

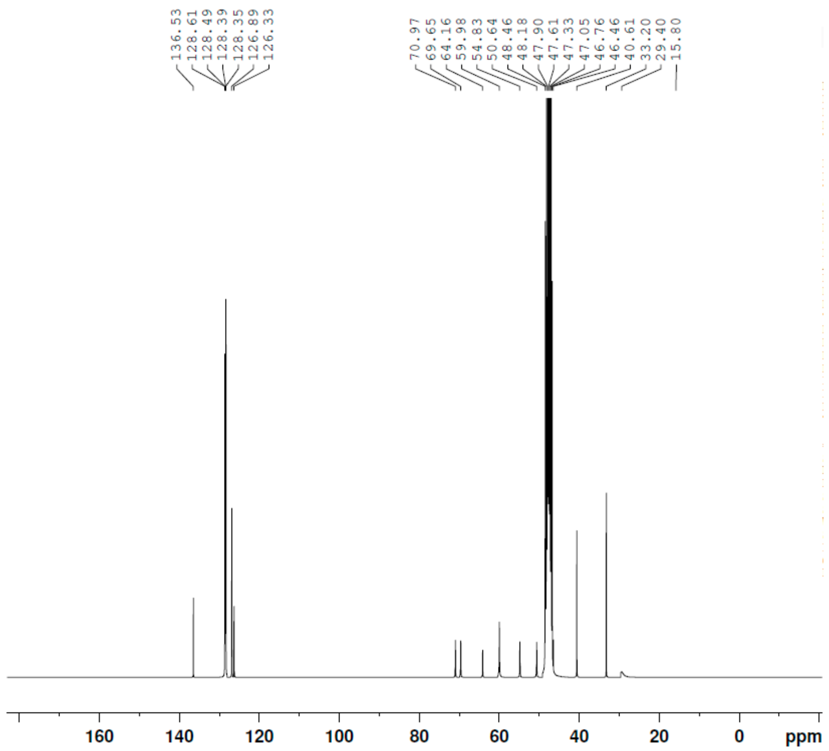

N-Phenetyl-1,6-dideoxy-1,6-imino-D-mannitol (29b):

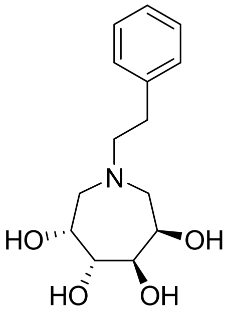

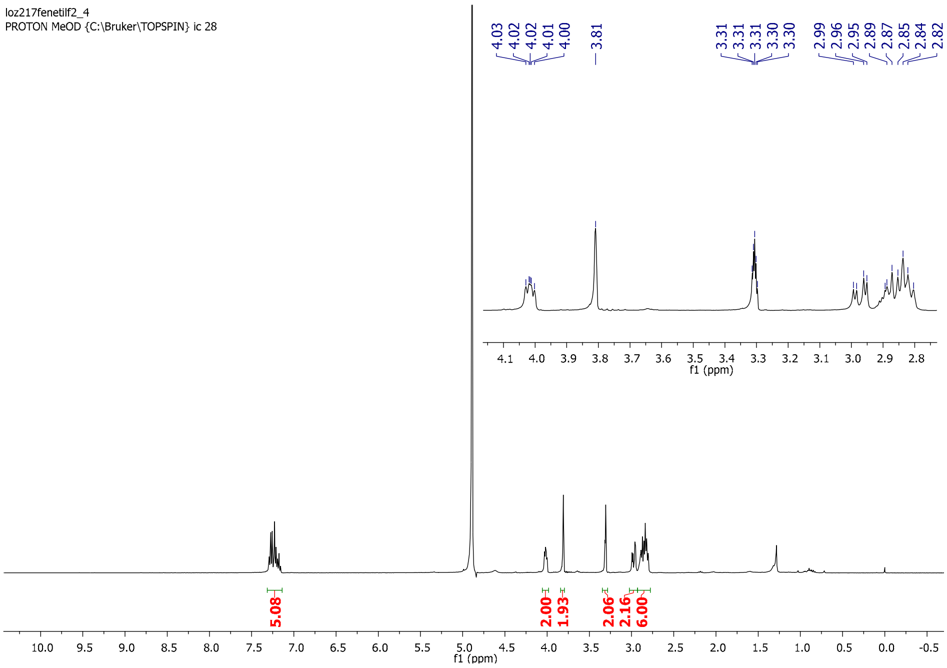

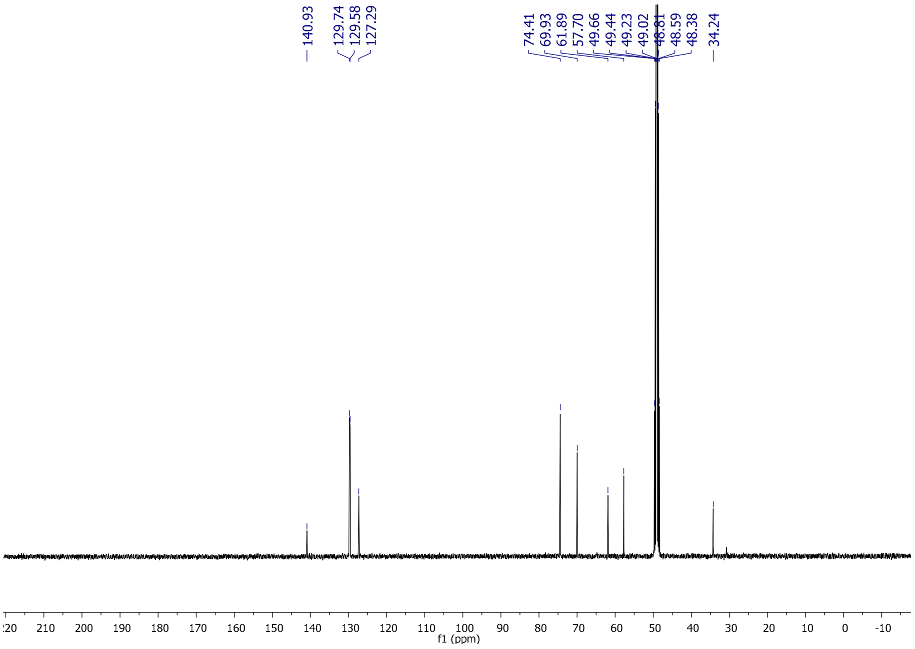

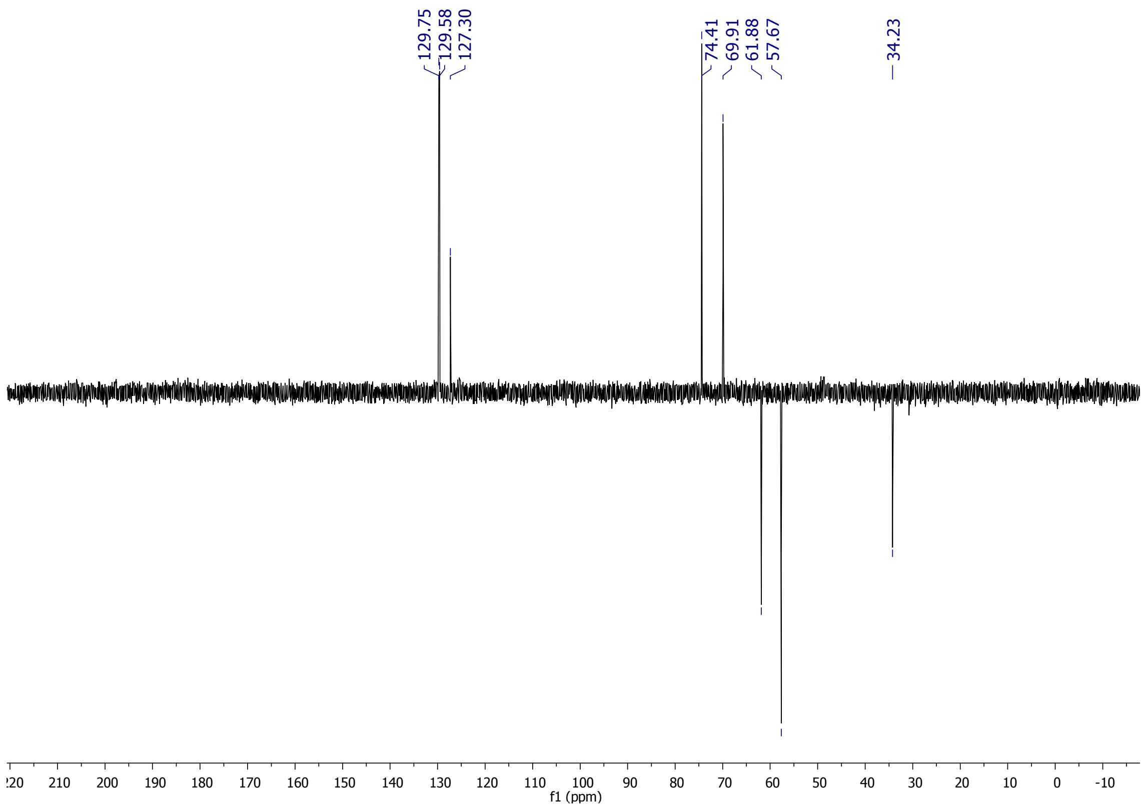
129.75
129.58
127.30
74.41
69.91
61.88
57.67
34.23
f1 (ppm)

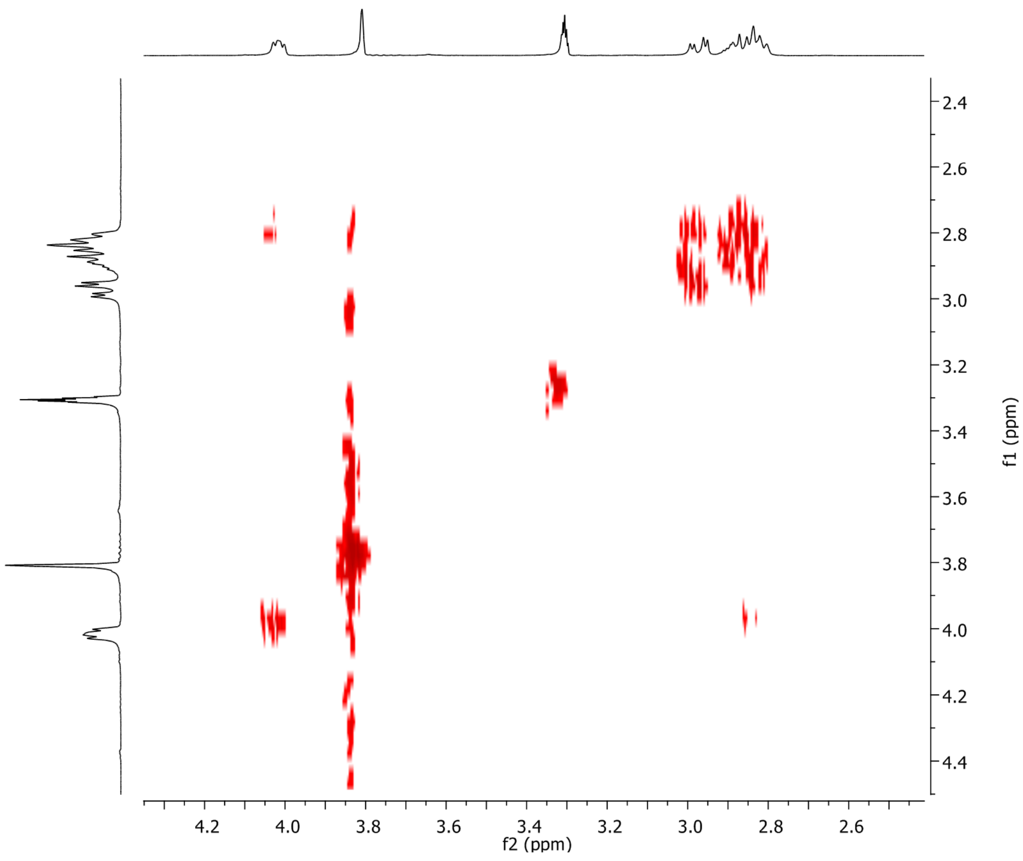
f2 (ppm)
f1 (ppm)

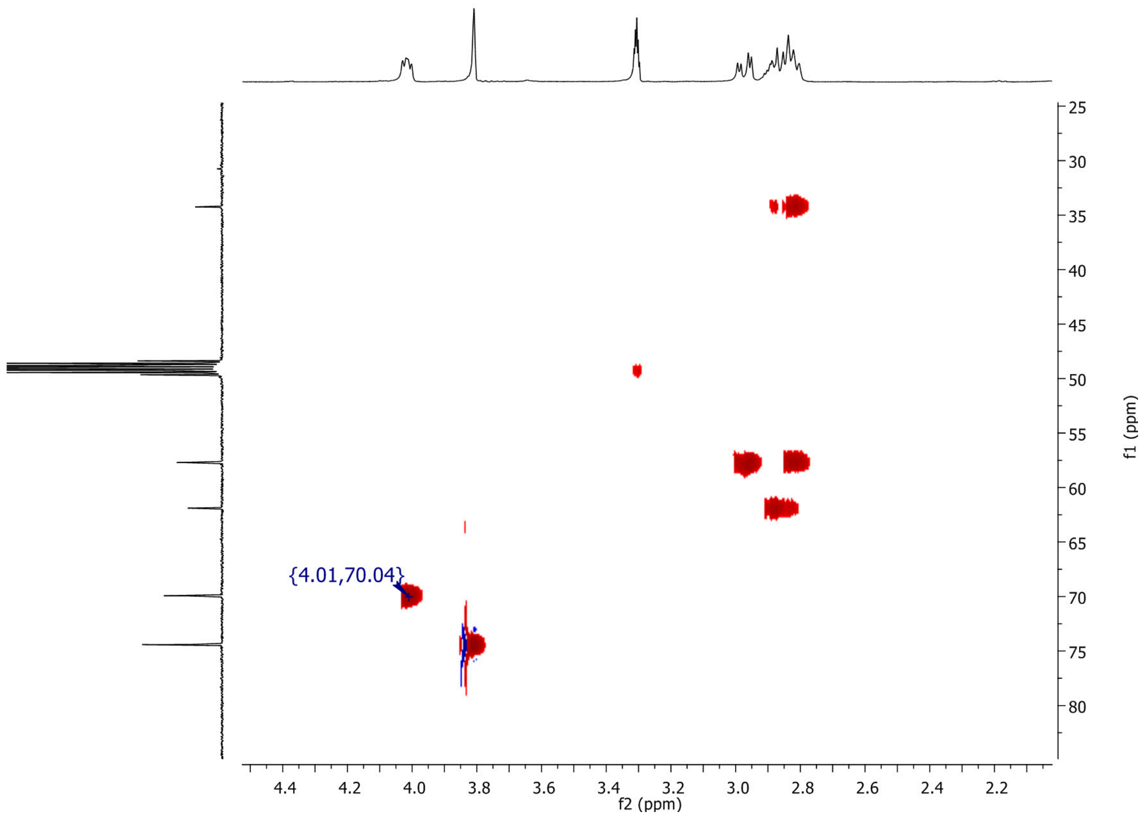
{4.01,70.04}
25
30
35
40
45
50
55
60
65
70
75
80
f1 (ppm)
4.4
4.2
4.0
3.8
3.6
3.4
3.2
3.0
2.8
2.6
2.4
2.2
f2 (ppm)

***N*-Propynyl-2,3,4,6-tetra-*O*-acetyl-1,5-dideoxy-1,5-imino-L-gulitol (30a):**

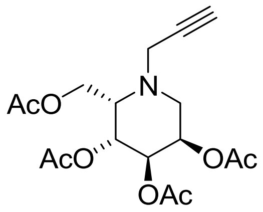

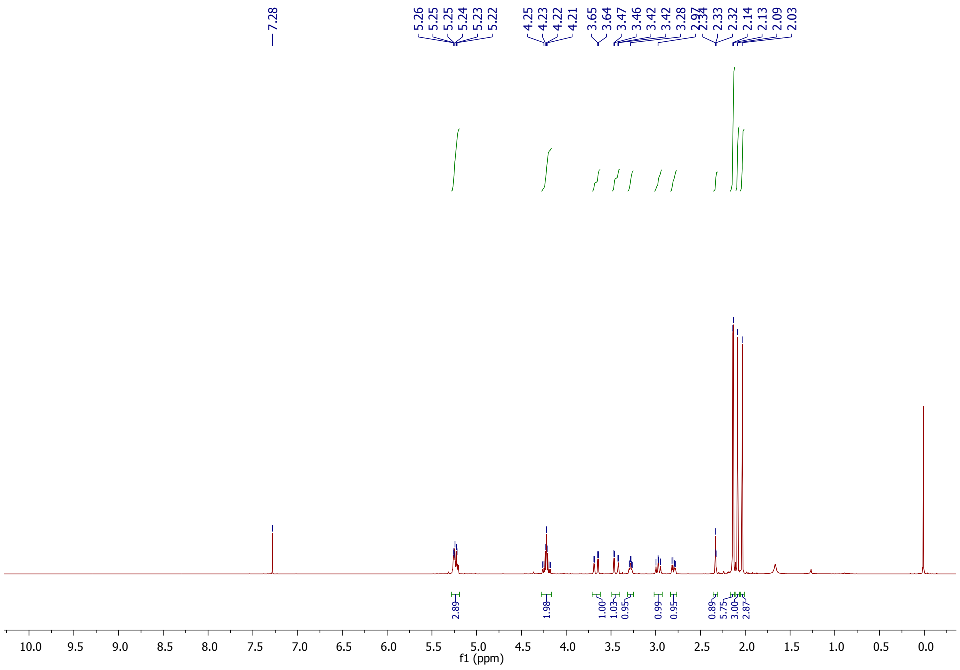

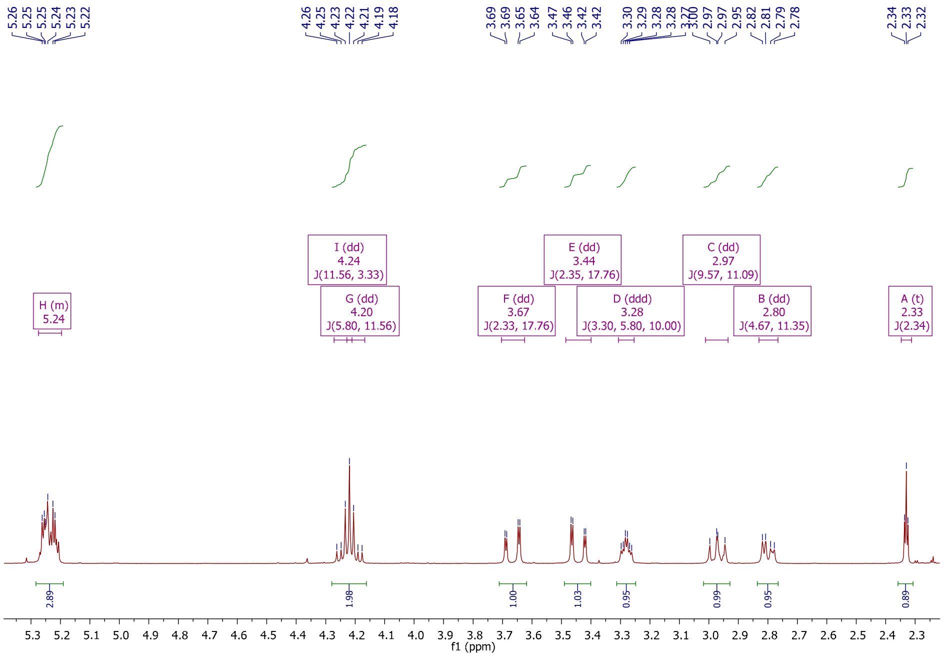

^{1}H NMR (400 MHz, CD_3OD)

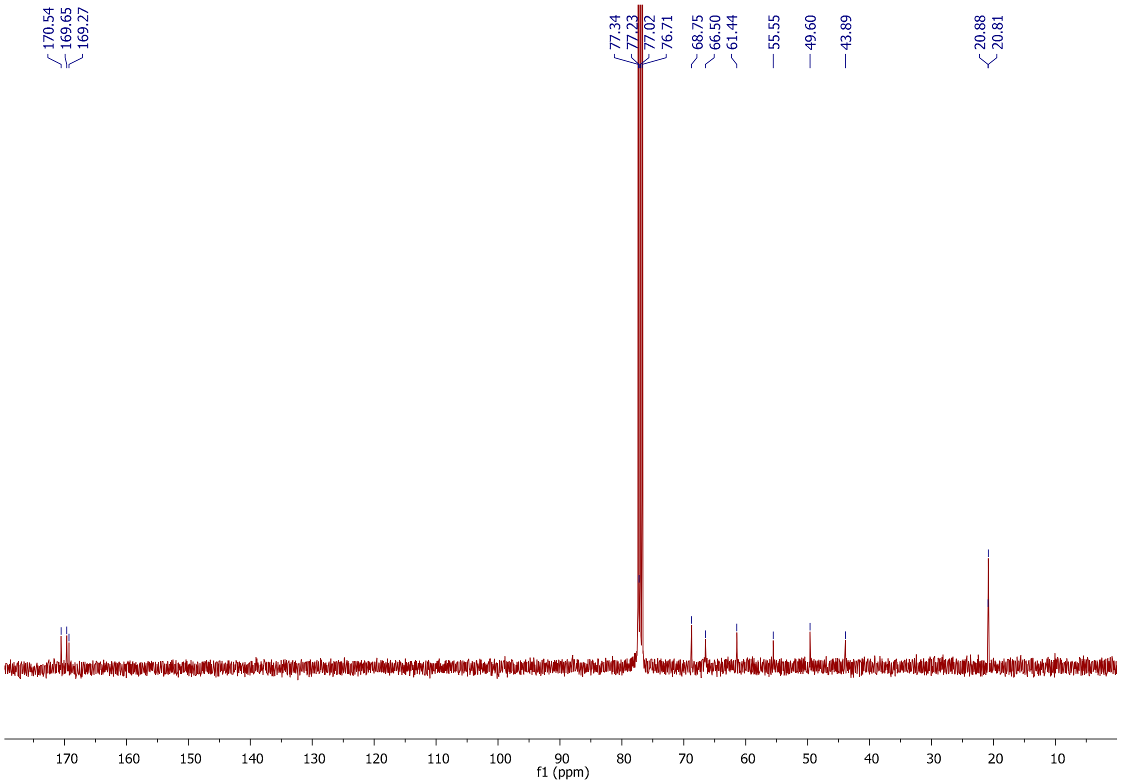

^{13}C NMR (100 MHz, CD_3OD)

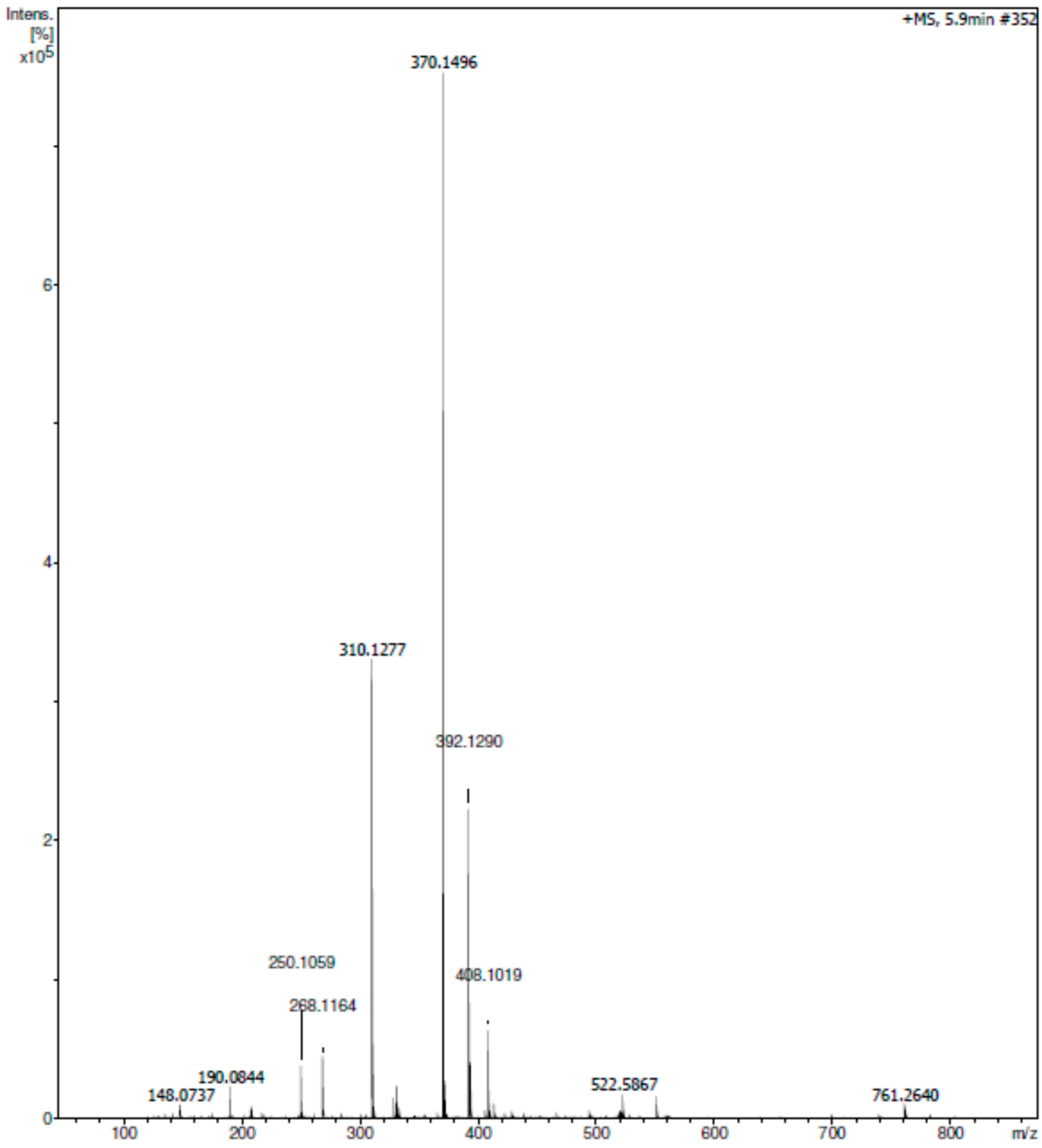

ESI HRMS: $[M+H]^+$ calcd for $C_{17}H_{24}NO_8$ 370.1496; found 370.1496.

_N_-Propynyl-2,3,4,6-tetra-_O_-acetyl-1,6-dideoxy-1,6-imino-D-mannitol (30b):

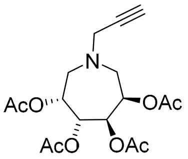

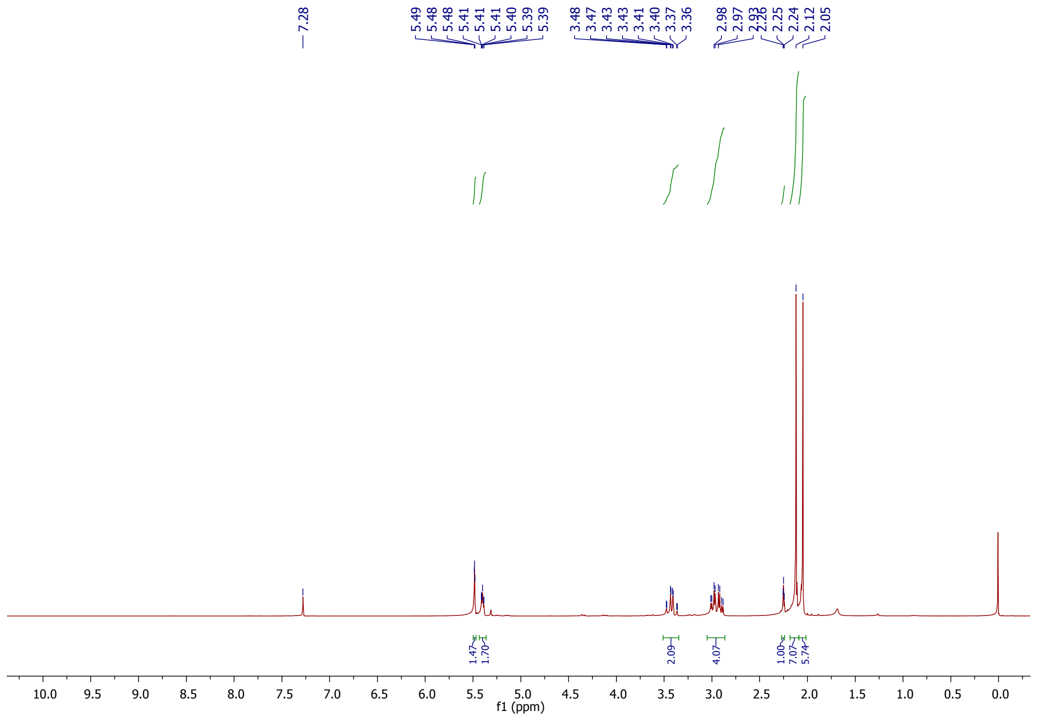

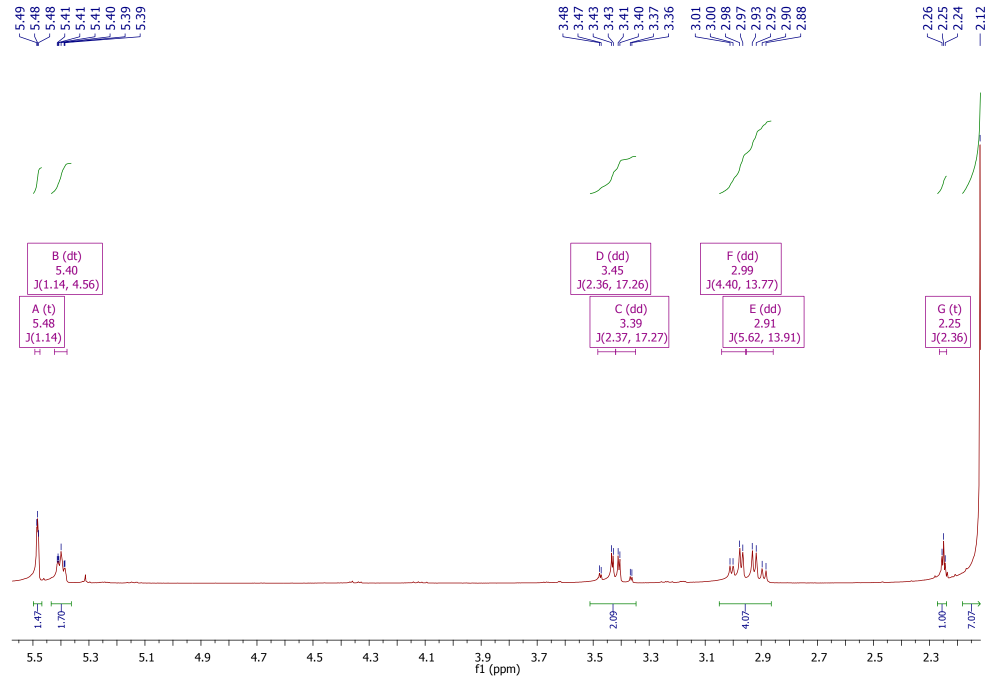

^{1}H NMR (400 MHz, CD_3OD)

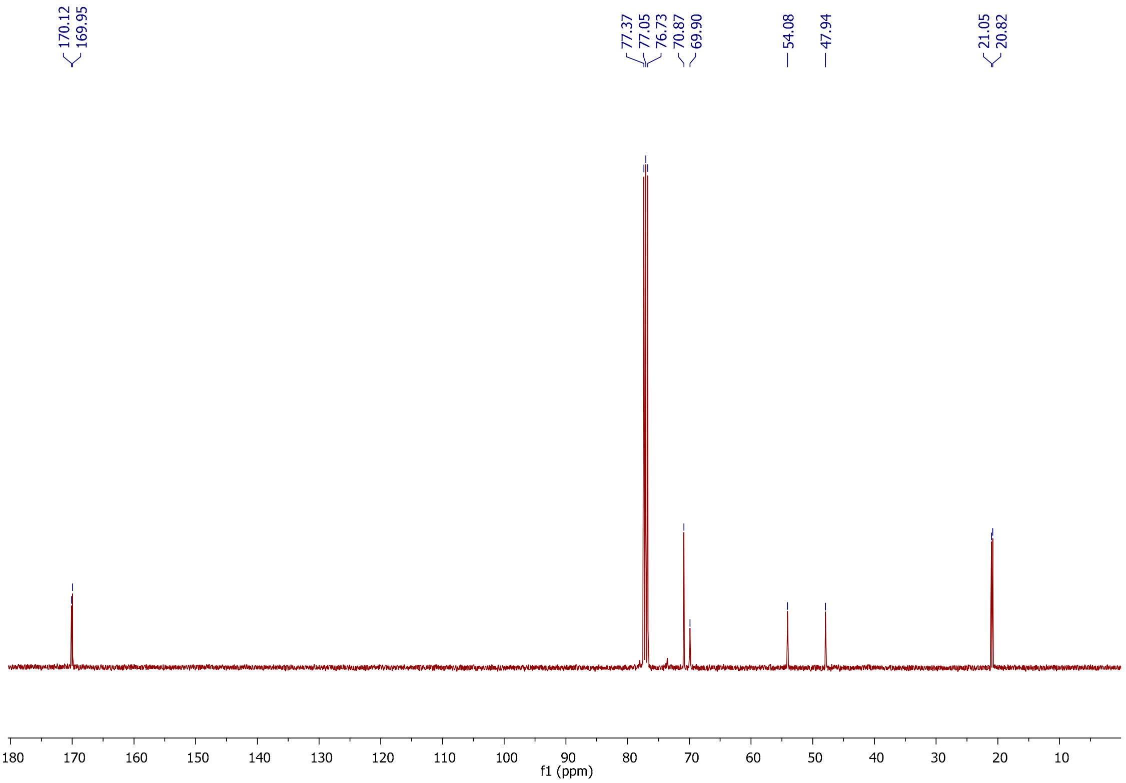

^{13}C NMR (100 MHz, CD_3OD)

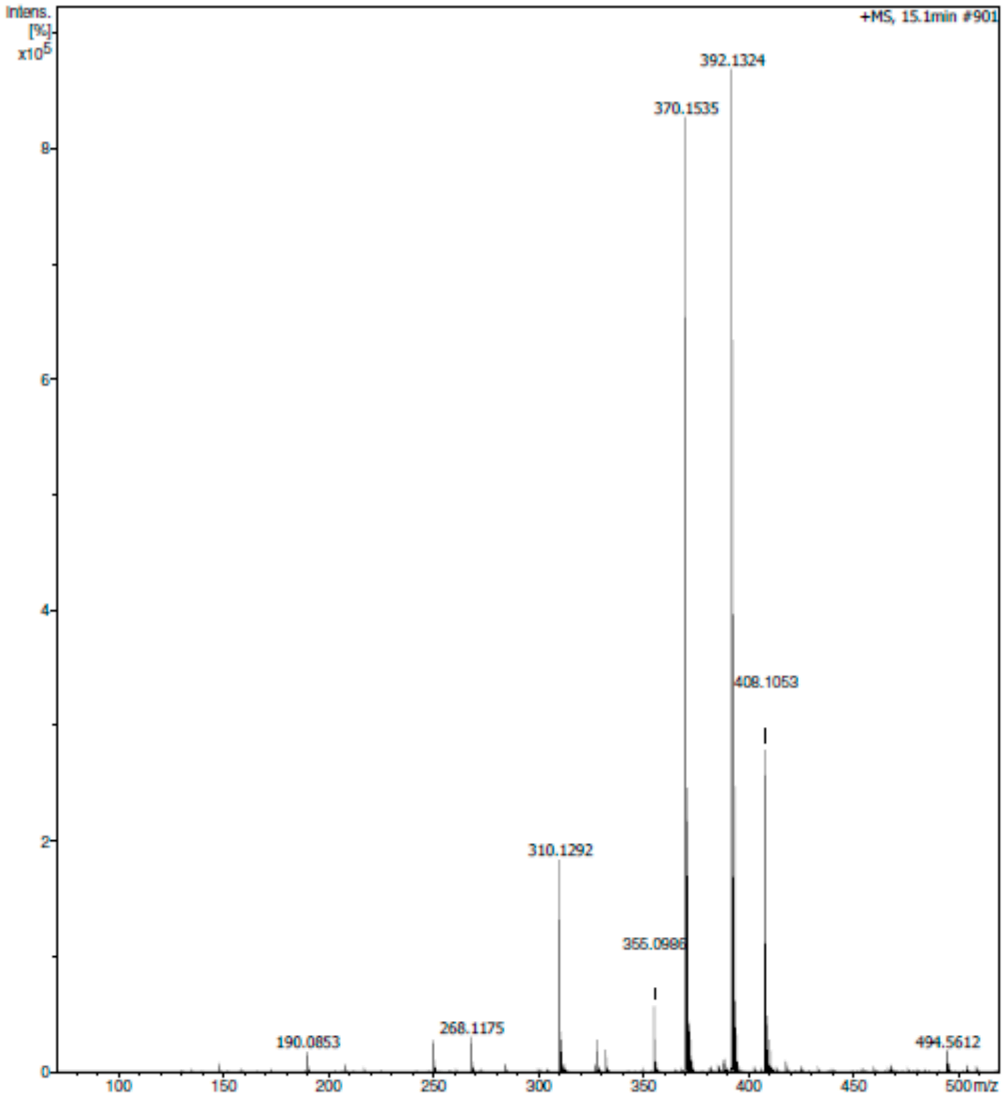

ESI HRMS: $[M+H]^+$ calcd for $C_{17}H_{24}NO_8$ 370.1496; found 370.1535.

_N_-Acetoxyethyl-2,3,4,6-tetra-_O_-acetyl-1,5-dideoxy-1,5-imino-L-gulitol (31a):

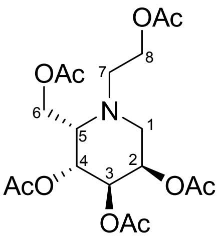

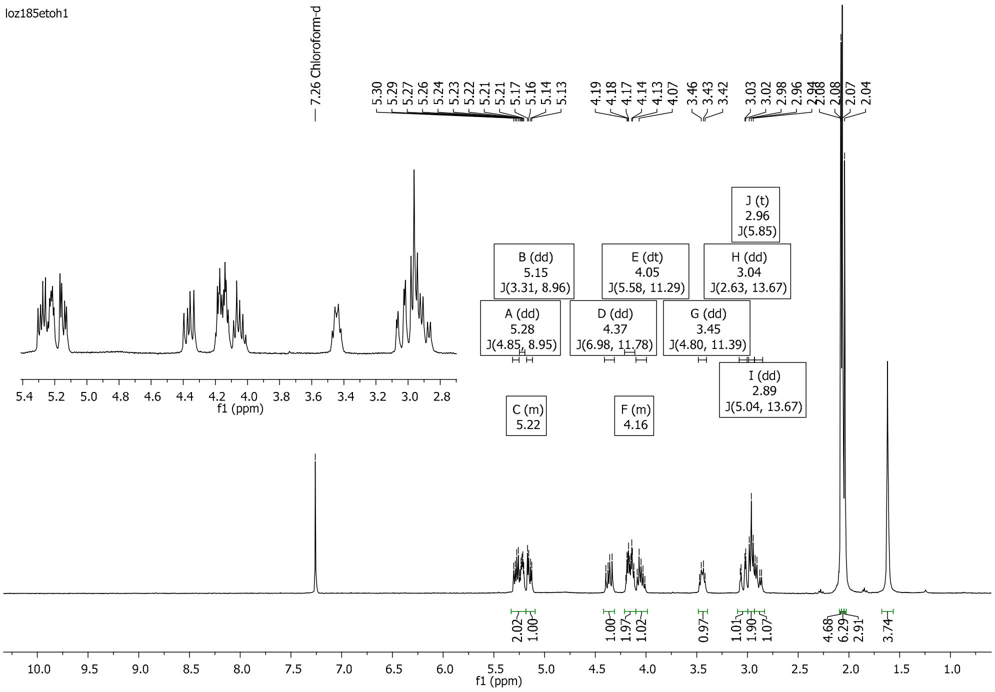

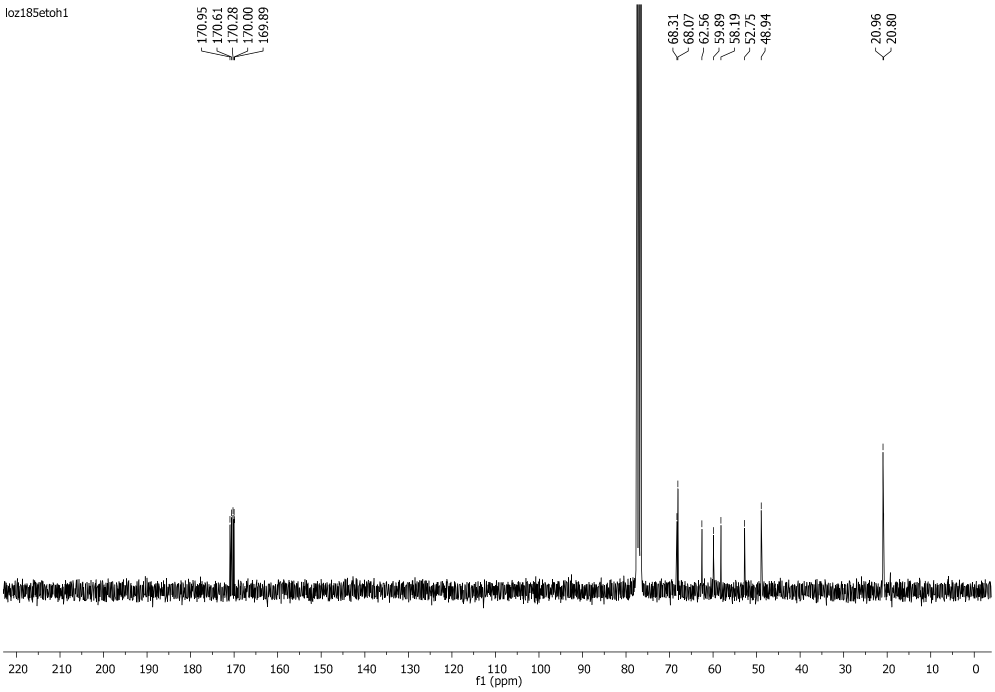

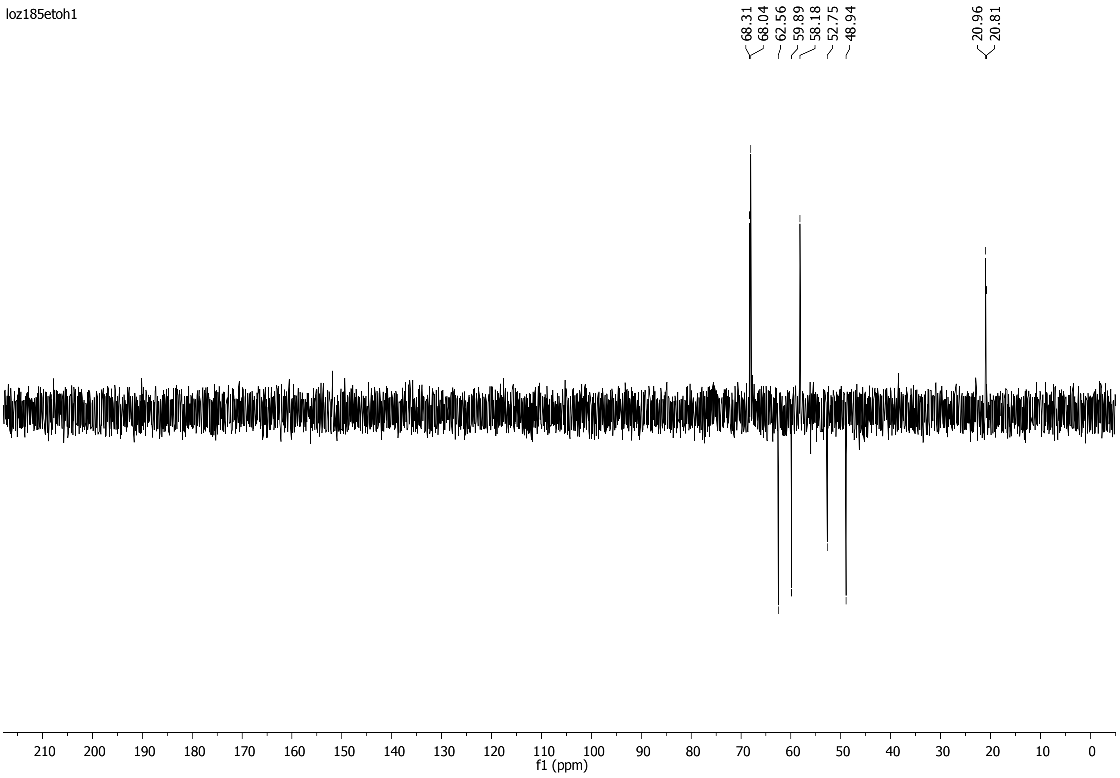
loz185etoh1
68.31
68.04
62.56
59.89
58.18
52.75
48.94
20.96
20.81
210 200 190 180 170 160 150 140 130 120 110 100 90 80 70 60 50 40 30 20 10 0
f1 (ppm)

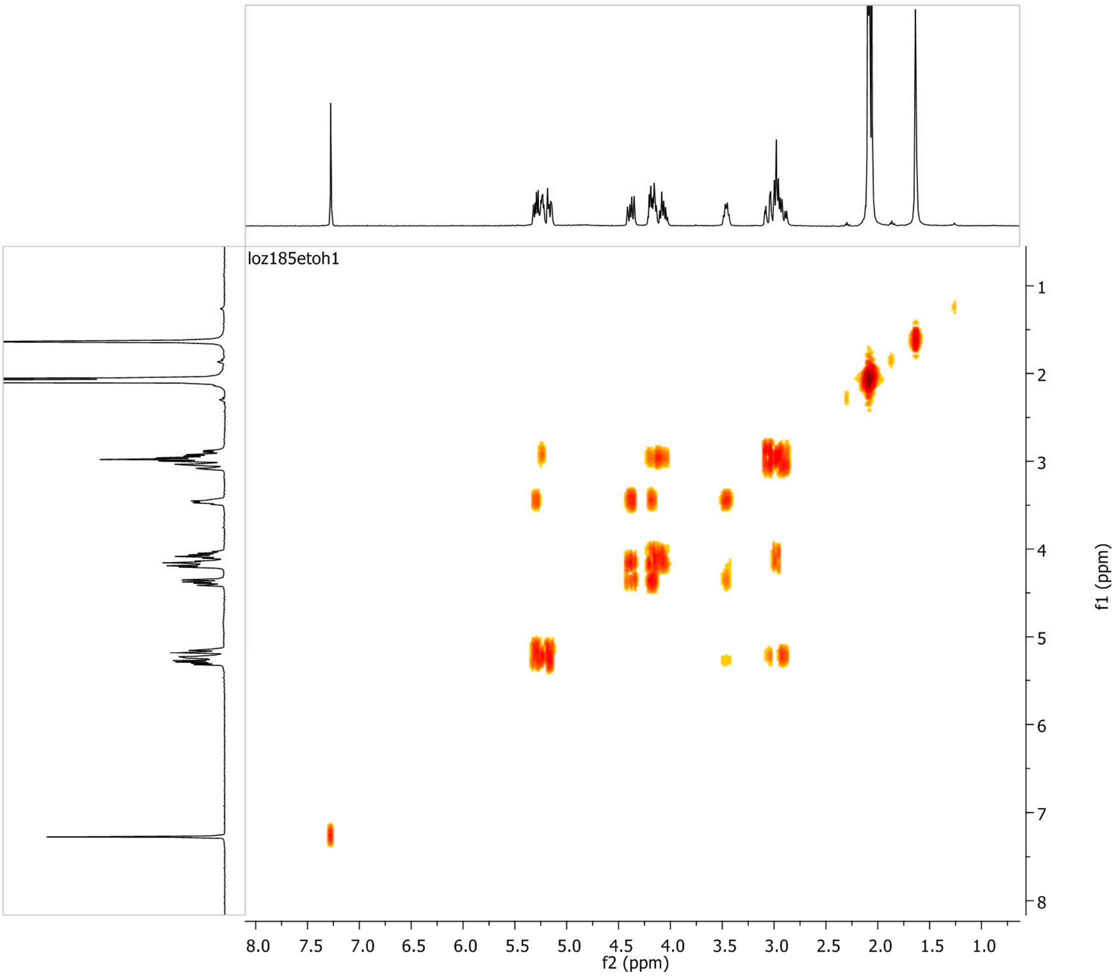
loz185etoh1
8.0 7.5 7.0 6.5 6.0 5.5 5.0 4.5 4.0 3.5 3.0 2.5 2.0 1.5 1.0
f2 (ppm)
1 2 3 4 5 6 7 8
f1 (ppm)

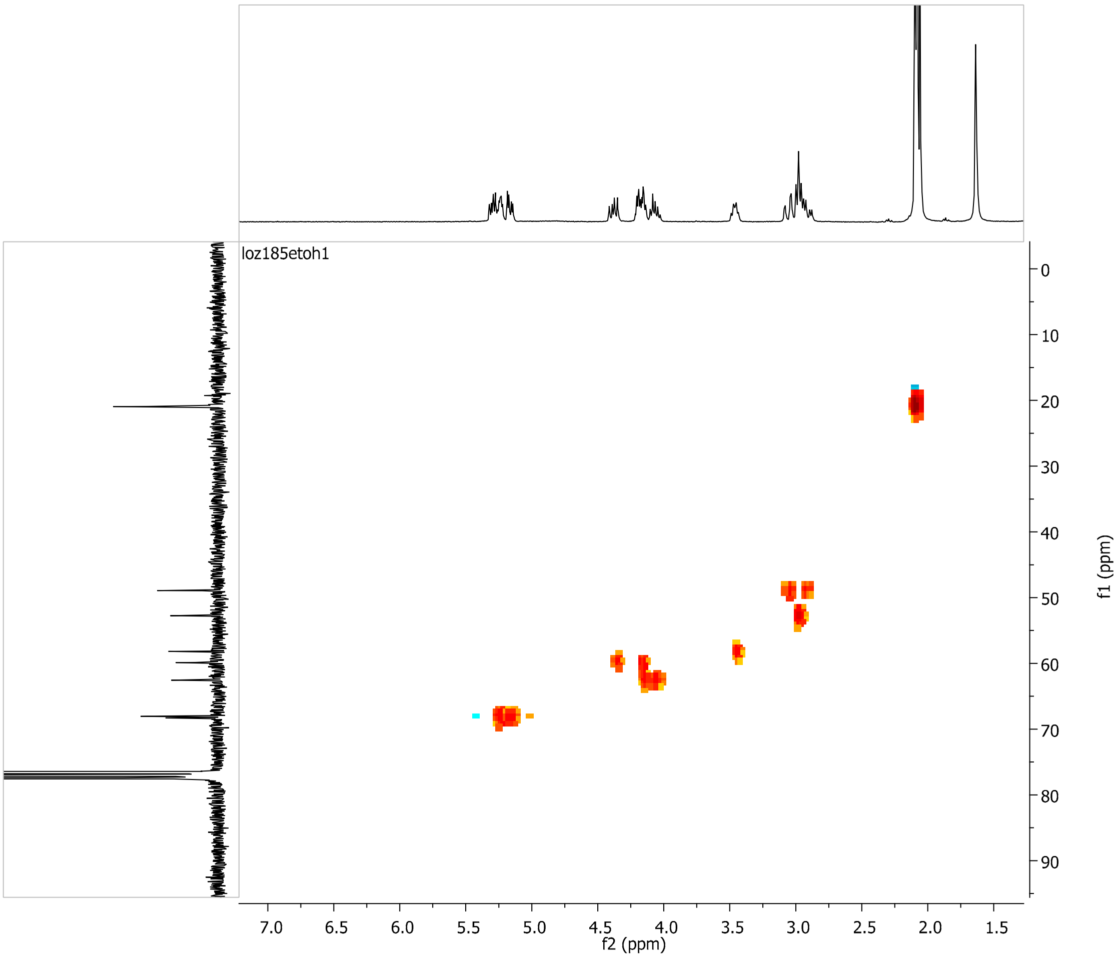

loz185etoh1
7.0
6.5
6.0
5.5
5.0
4.5
4.0
3.5
3.0
2.5
2.0
1.5
f2 (ppm)
0
10
20
30
40
50
60
70
80
90
f1 (ppm)

N-Acetoxyethyl-2,3,4,6-tetra-O-acetyl-1,6-dideoxy-1,6-imino-D-mannitol (31b):

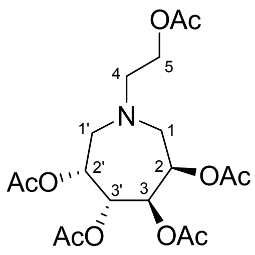

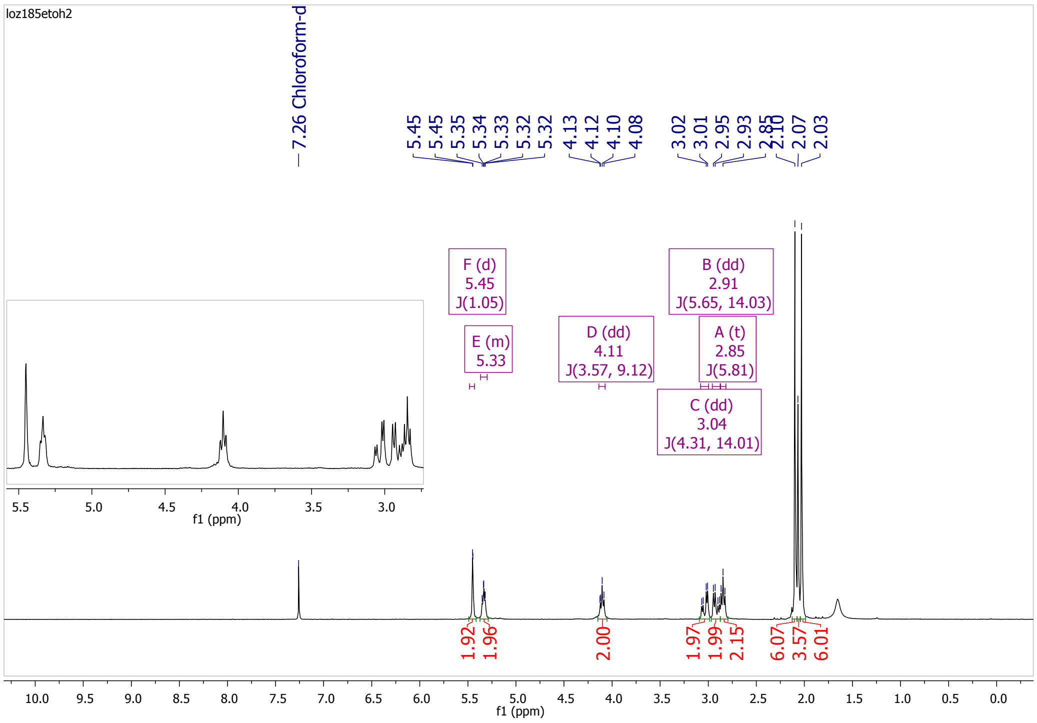

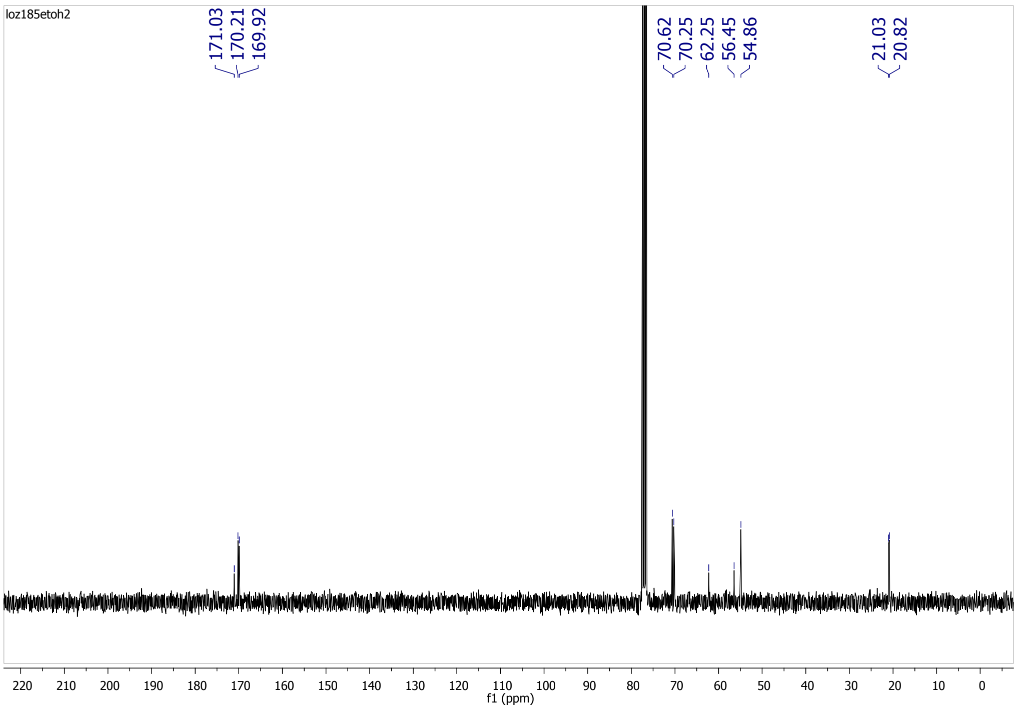

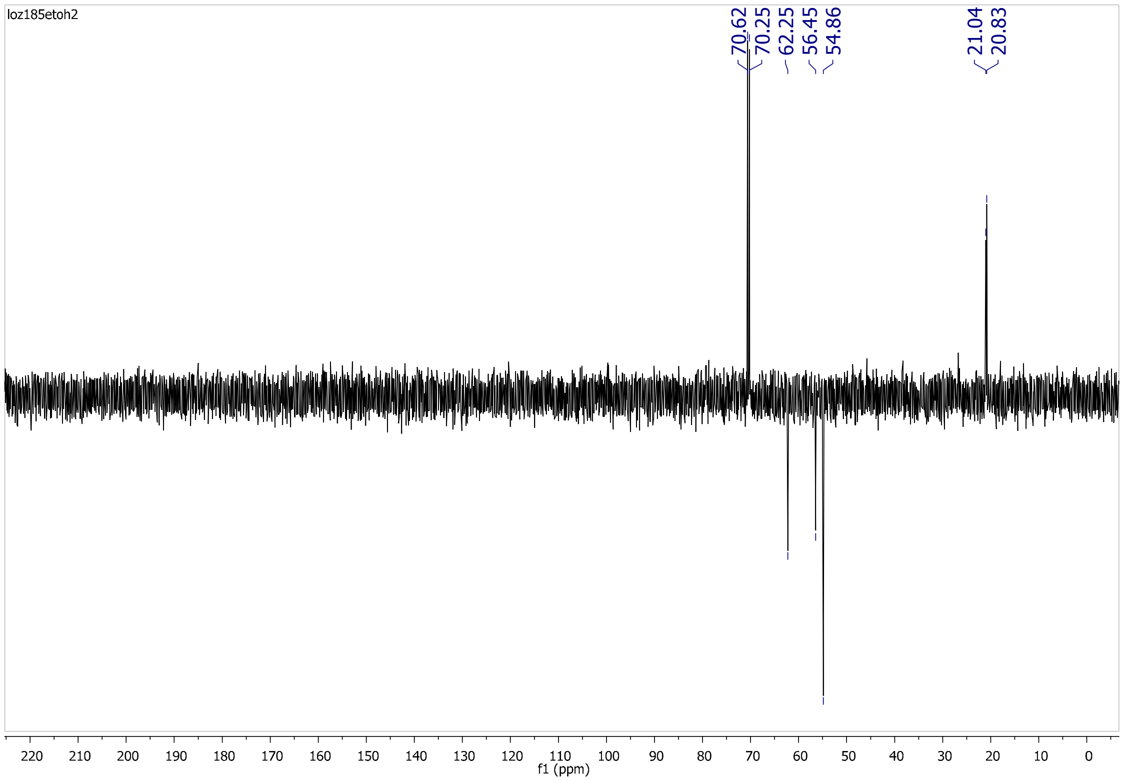
loz185etoh2
70.62
70.25
62.25
56.45
54.86
21.04
20.83
220 210 200 190 180 170 160 150 140 130 120 110 100 90 80 70 60 50 40 30 20 10 0
f1 (ppm)

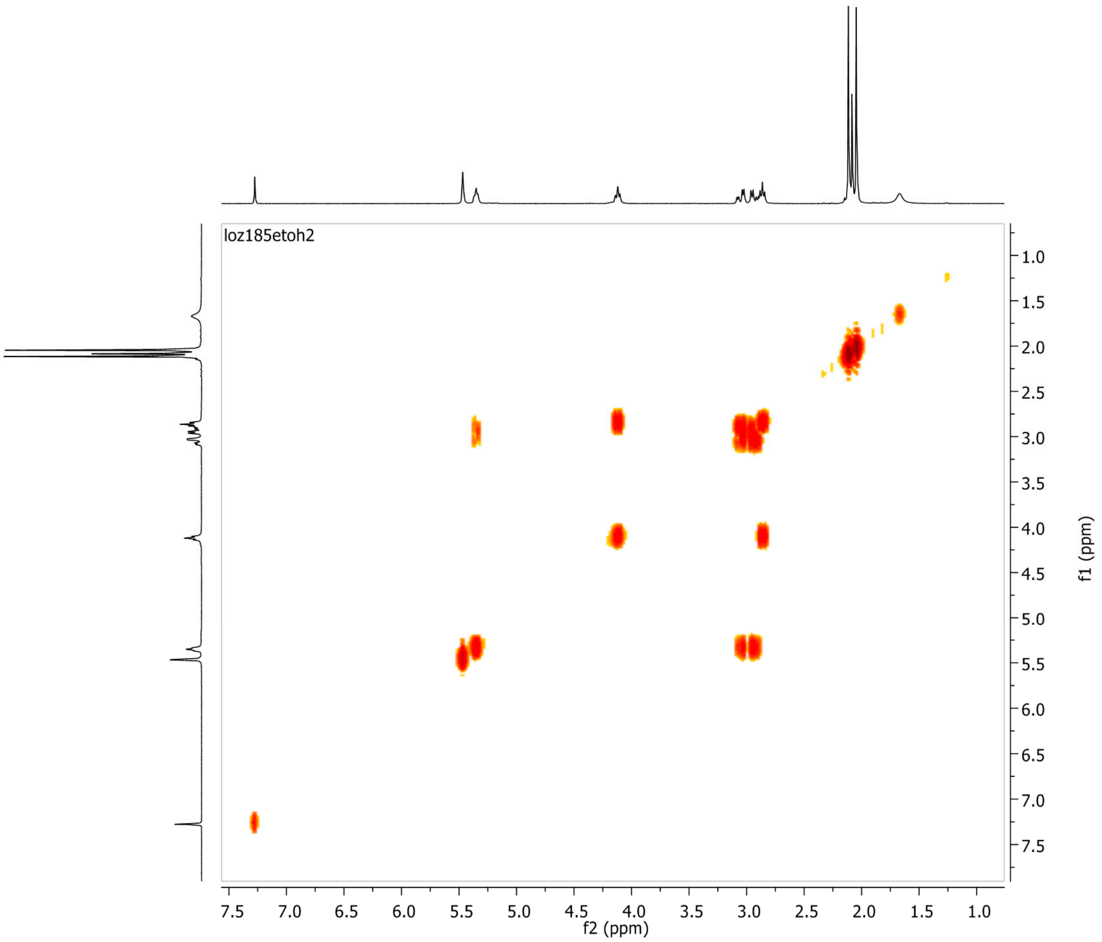
loz185etoh2
7.5 7.0 6.5 6.0 5.5 5.0 4.5 4.0 3.5 3.0 2.5 2.0 1.5 1.0
f2 (ppm)
1.0 1.5 2.0 2.5 3.0 3.5 4.0 4.5 5.0 5.5 6.0 6.5 7.0 7.5
f1 (ppm)

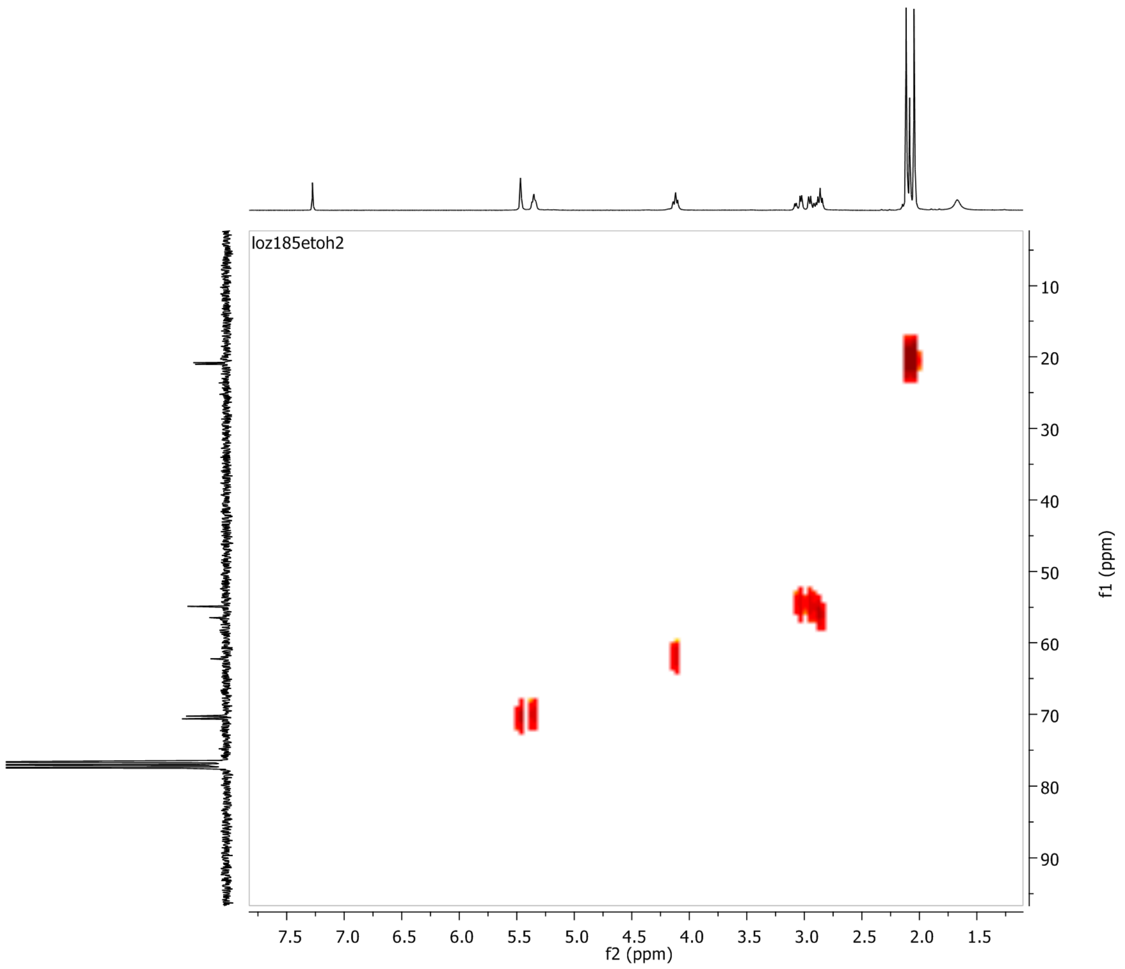
loz185etoh2
f2 (ppm)
f1 (ppm)

_N_-Propynyl-2,3,4,6-tetra-_O_-acetyl-1,5-dideoxy-1,5-imino-D-glucitol (36a):

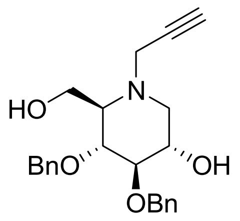

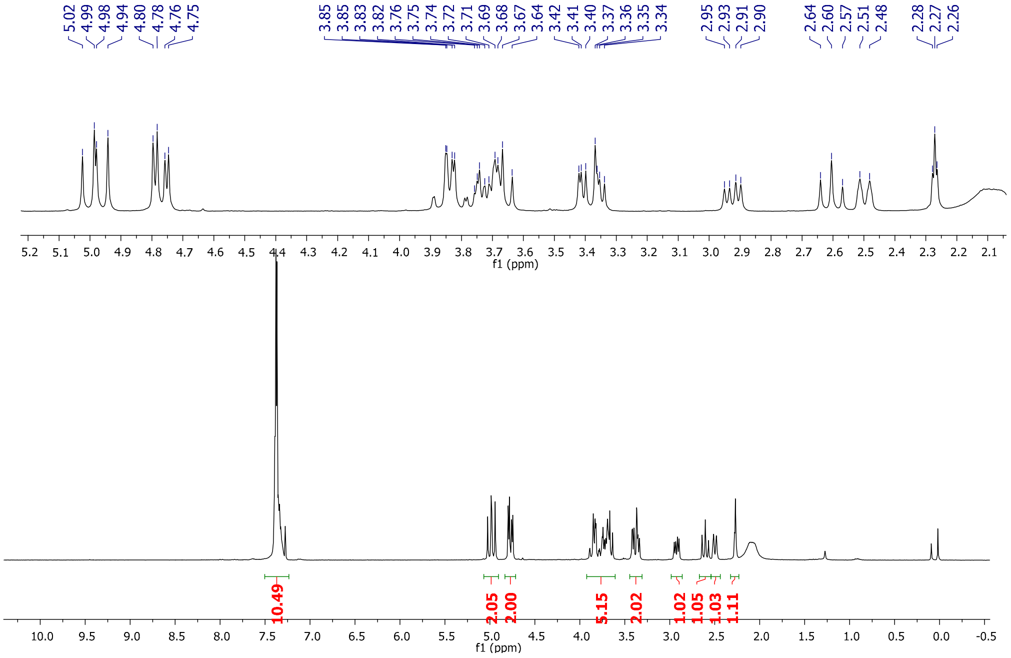

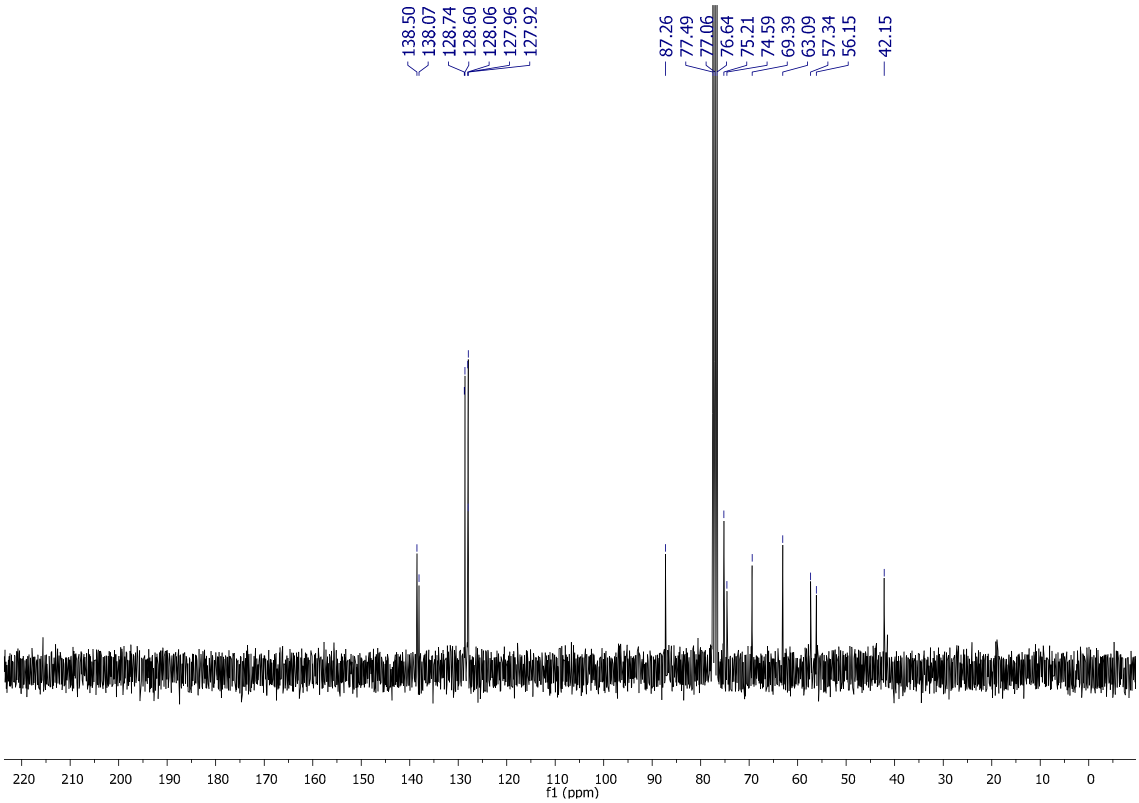

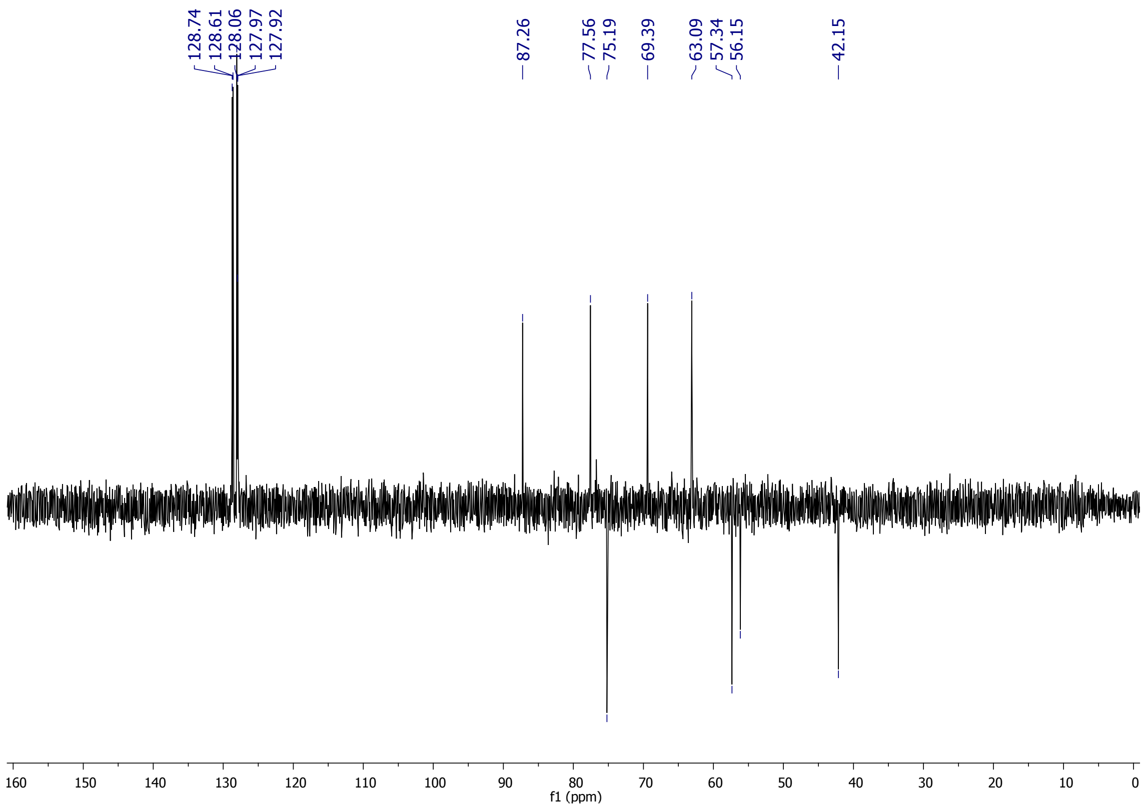
128.74
128.61
128.06
127.97
127.92
87.26
77.56
75.19
69.39
63.09
57.34
56.15
42.15
f1 (ppm)

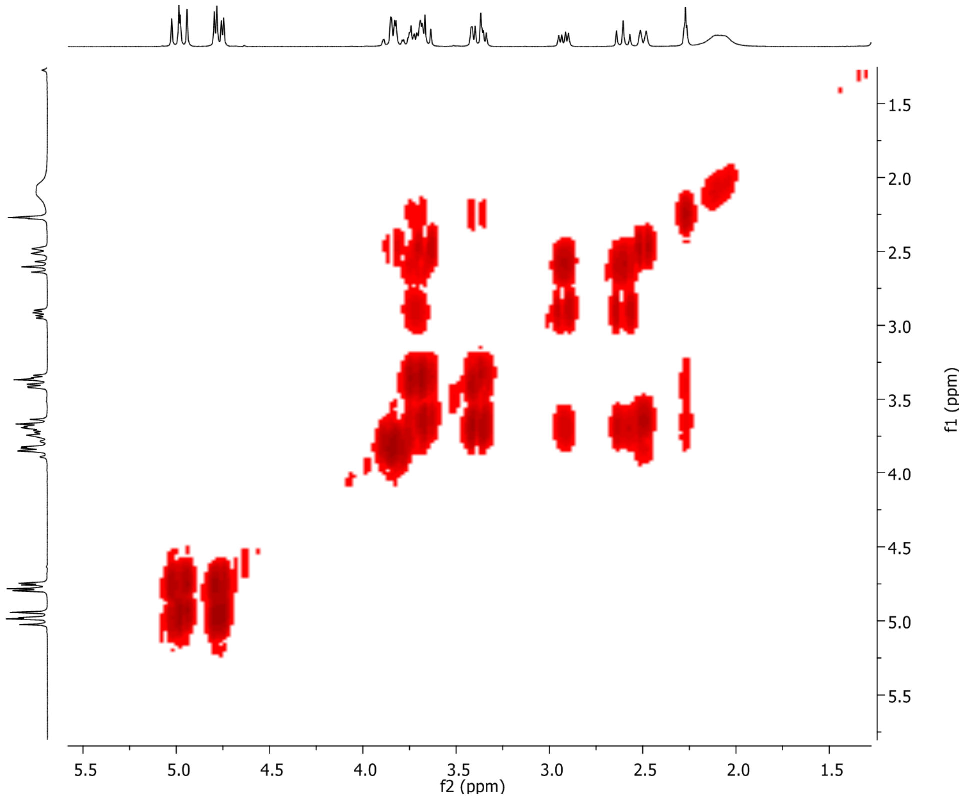
f2 (ppm)
f1 (ppm)

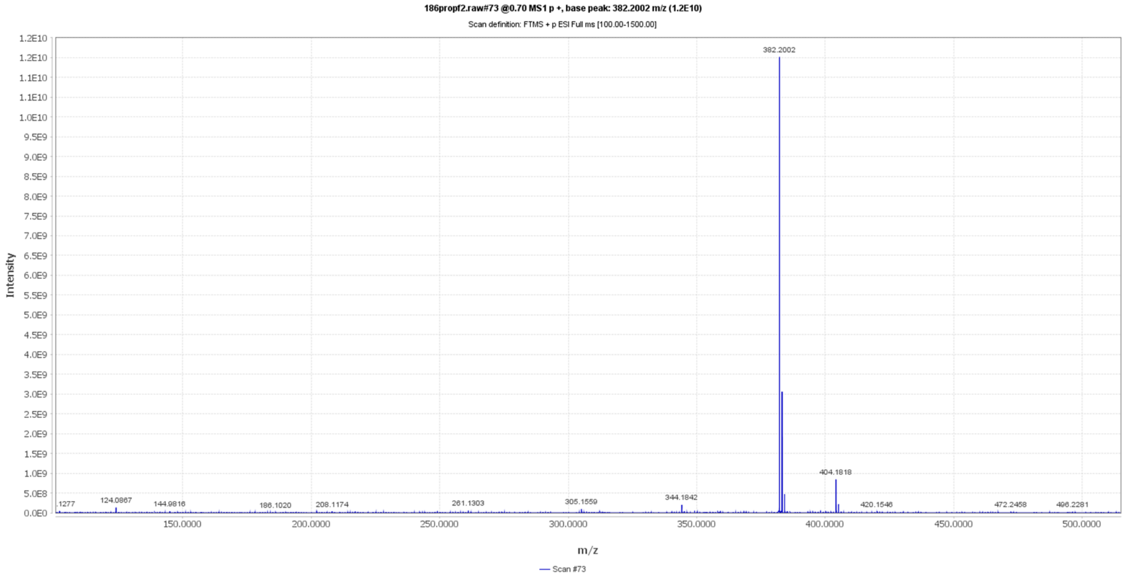

ESI HRMS: $[M+H]^+$ calcd for $C_{23}H_{28}NO_4$ 382.2013; found 382.2002

***N*-Propynyl-2,3,4,6-tetra-*O*-acetyl-1,6-dideoxy-1,6-imino-L-iditol (36b):**

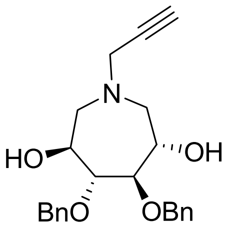

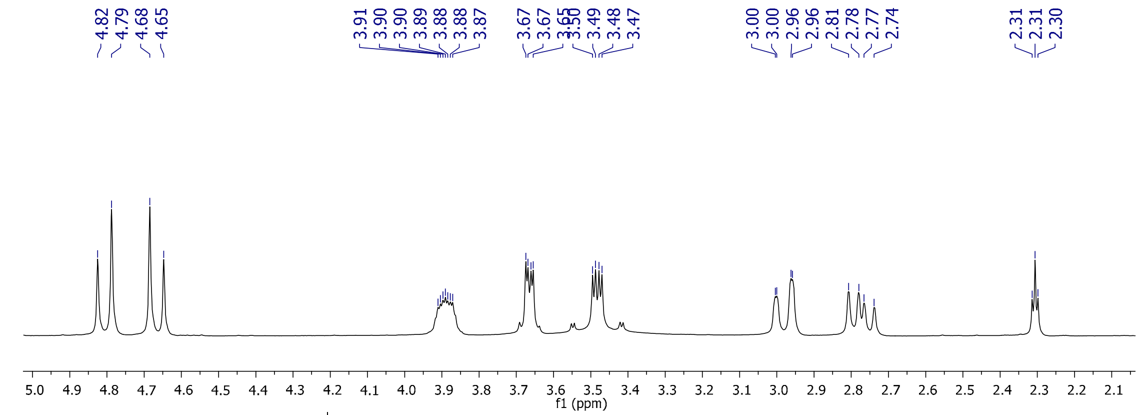

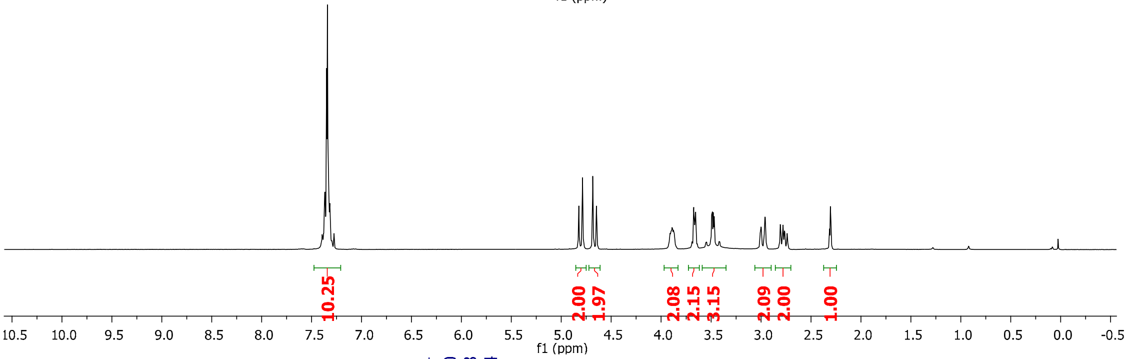

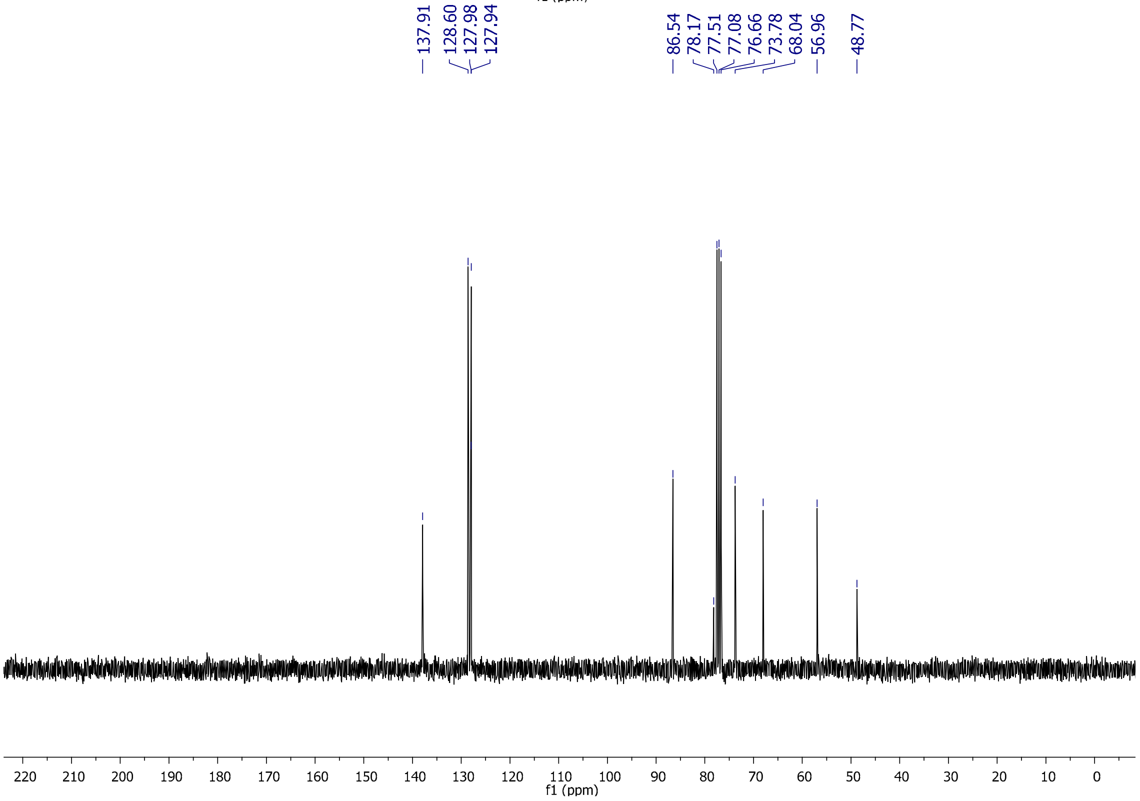

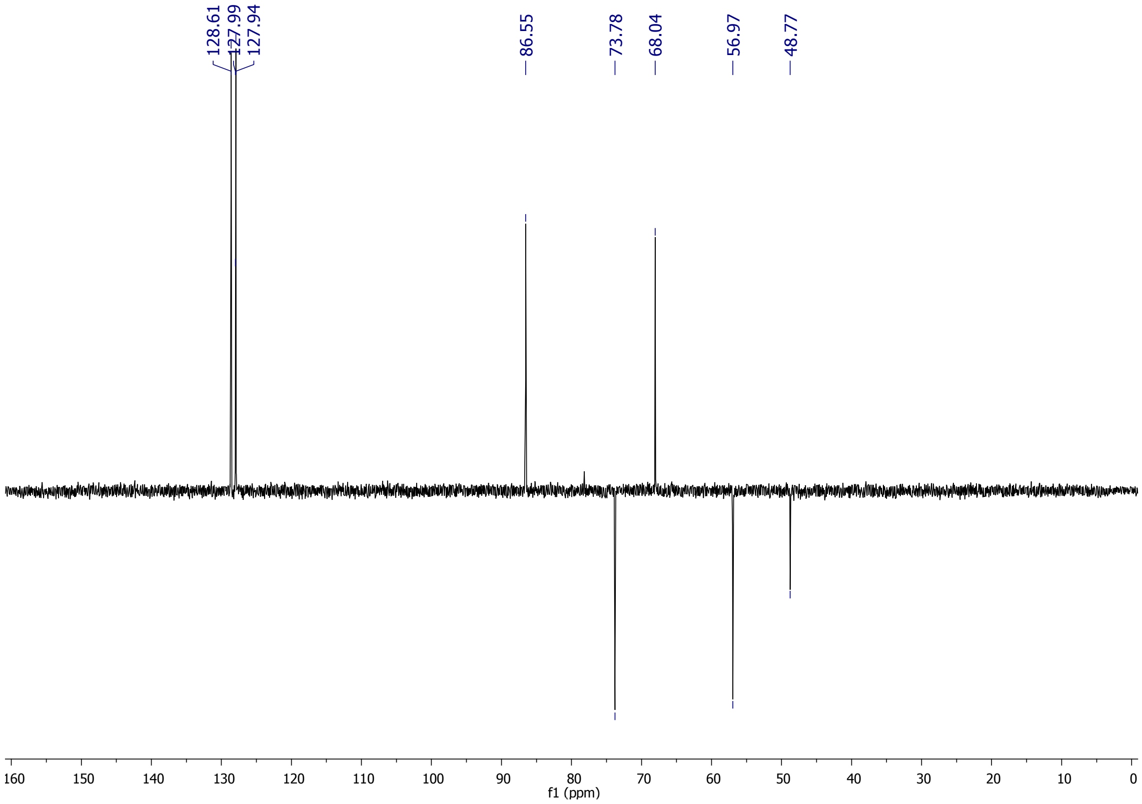
128.61
127.99
127.94
86.55
73.78
68.04
56.97
48.77
160
150
140
130
120
110
100
90
80
70
60
50
40
30
20
10
0
f1 (ppm)

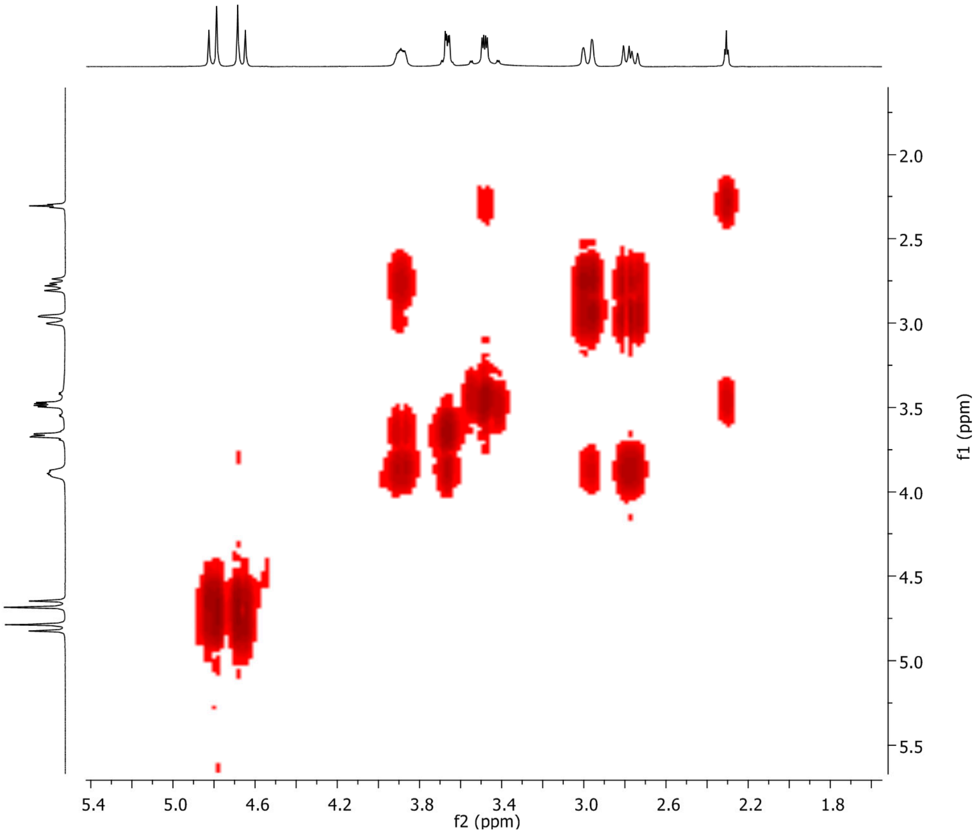
2.0
2.5
3.0
3.5
4.0
4.5
5.0
5.5
f1 (ppm)
5.4
5.0
4.6
4.2
3.8
3.4
3.0
2.6
2.2
1.8
f2 (ppm)

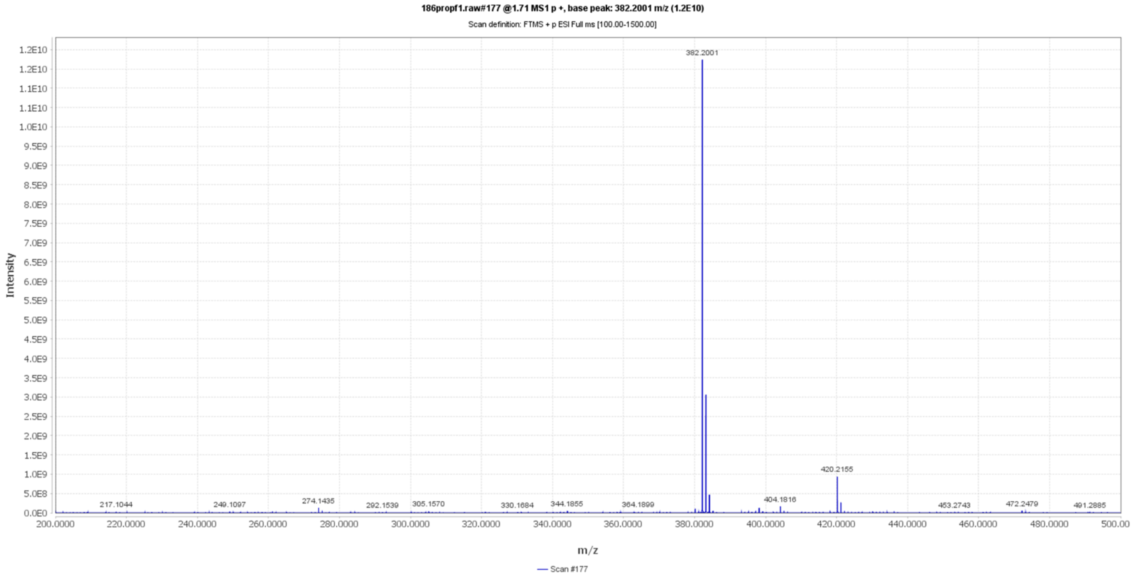

ESI HRMS: $[M+H]^+$ calcd for $C_{23}H_{28}NO_4$ 382.2013; found 382.2001

_N_-Butyl-2,3,4,6-tetra-_O_-acetyl-1,5-dideoxy-1,5-imino-D-glucitol (37a):

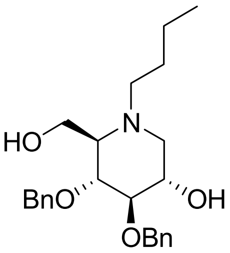

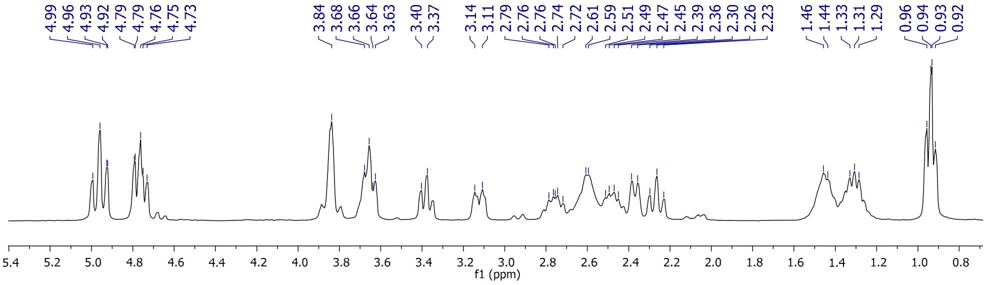

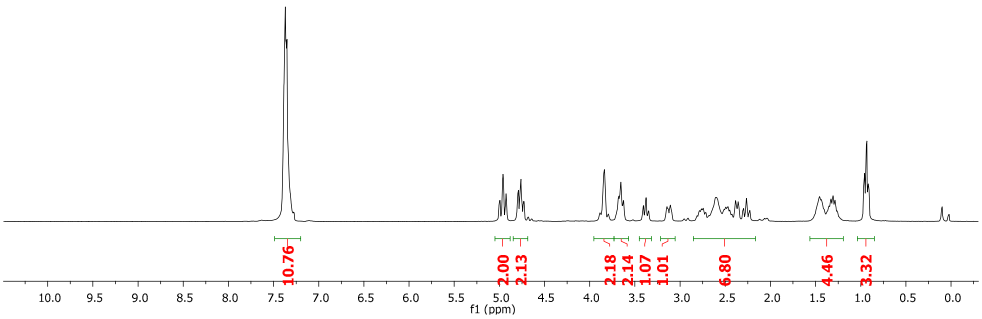

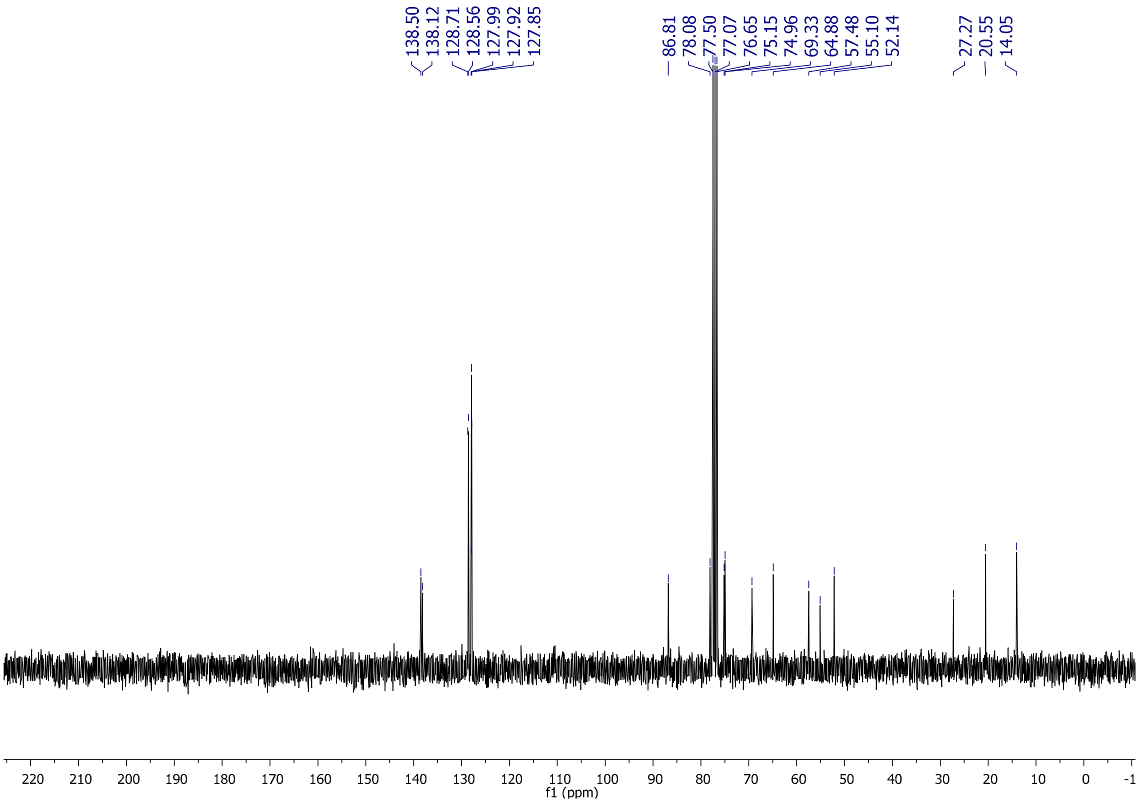

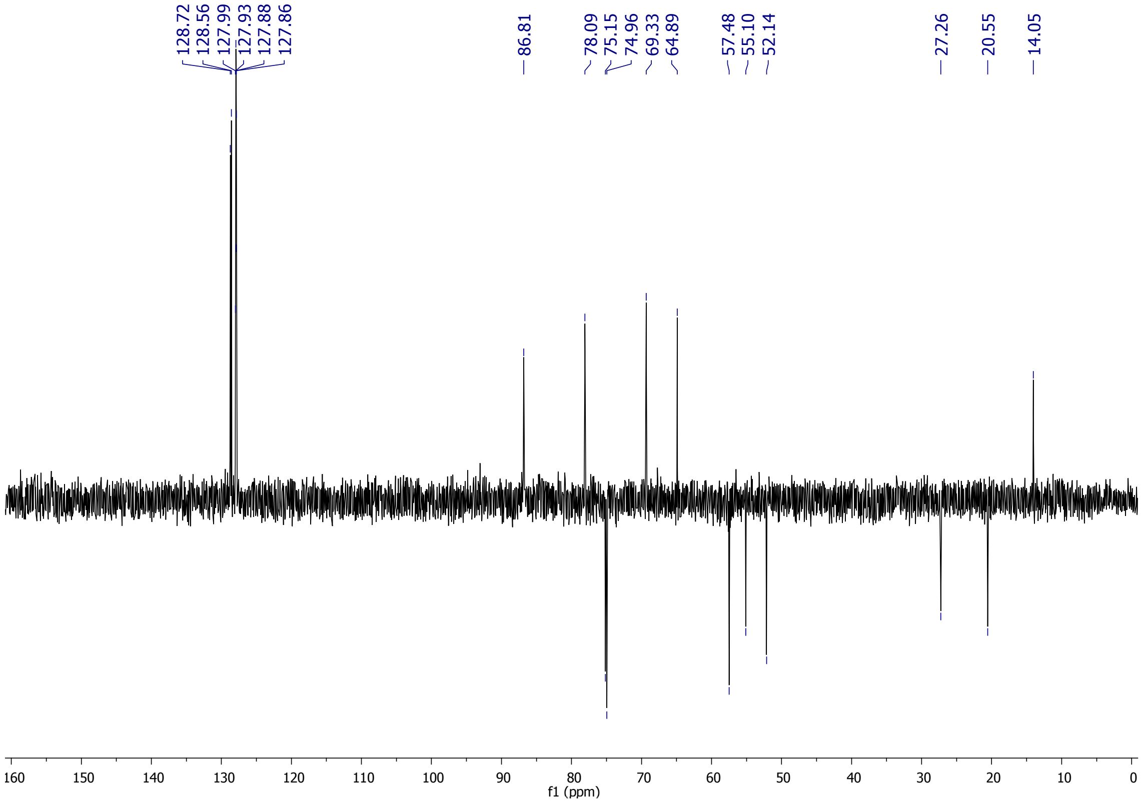
128.72
128.56
127.99
127.93
127.88
127.86
86.81
78.09
75.15
74.96
69.33
64.89
57.48
55.10
52.14
27.26
20.55
14.05
160
150
140
130
120
110
100
90
80
70
60
50
40
30
20
10
0
f1 (ppm)

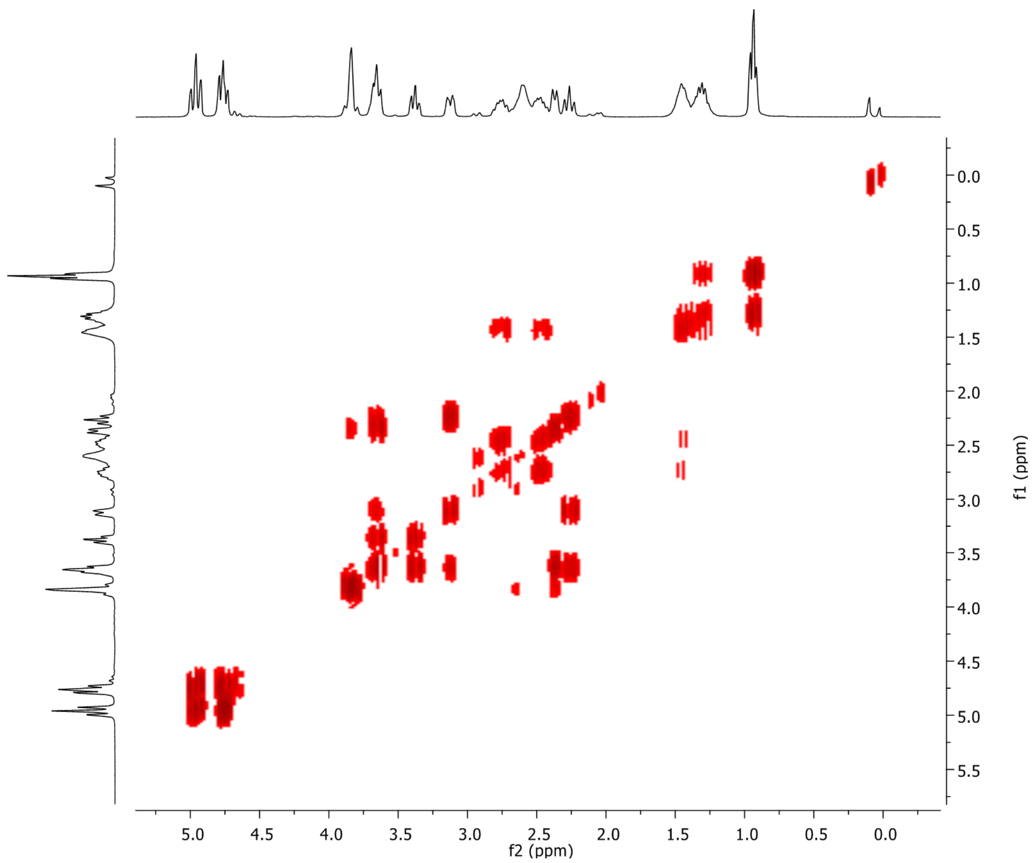
0.0
0.5
1.0
1.5
2.0
2.5
3.0
3.5
4.0
4.5
5.0
5.5
f1 (ppm)
5.0
4.5
4.0
3.5
3.0
2.5
2.0
1.5
1.0
0.5
0.0
f2 (ppm)

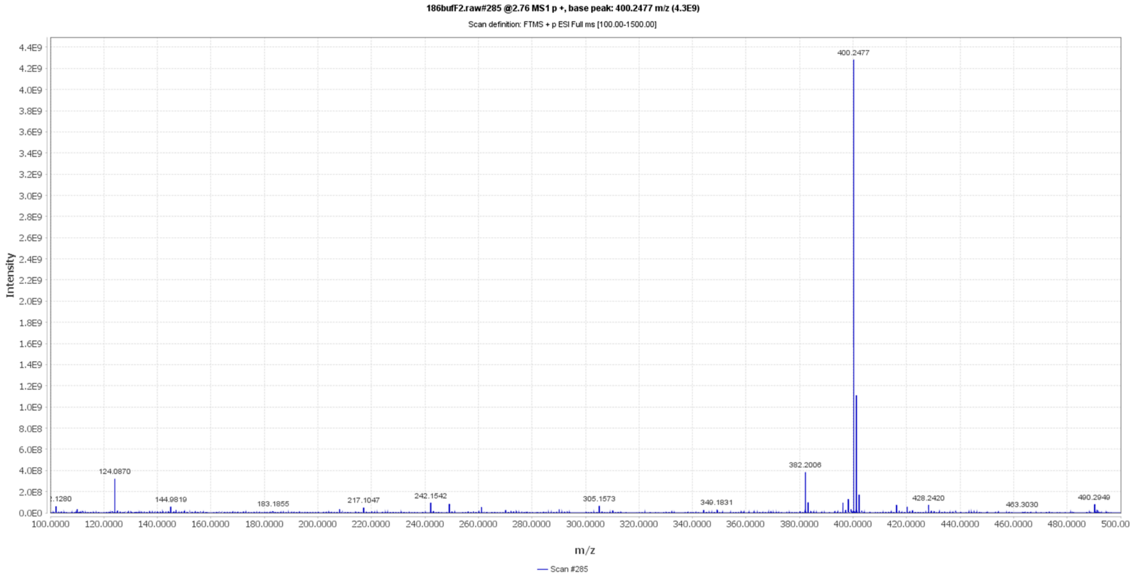

ESI HRMS: $[M+H]^+$ calcd for $C_{24}H_{34}NO_4$ 400.2483; found 400.2477

_N_-Butyl-2,3,4,6-tetra-_O_-acetyl-1,6-dideoxy-1,6-imino-L-iditol (37b):

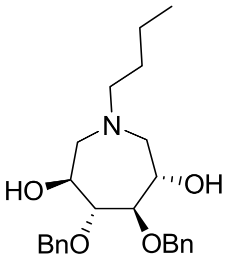

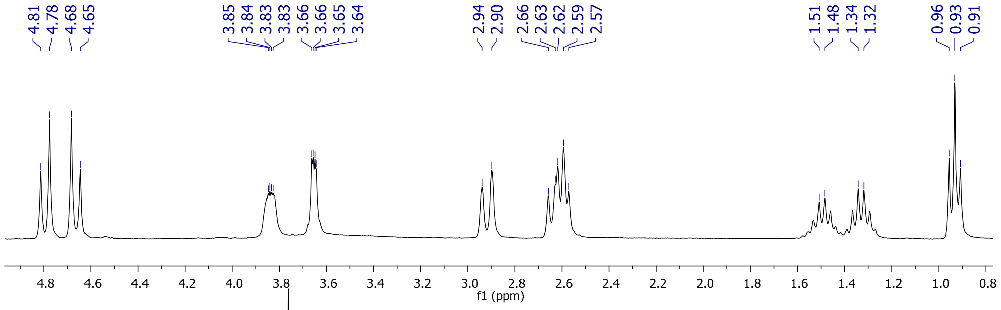

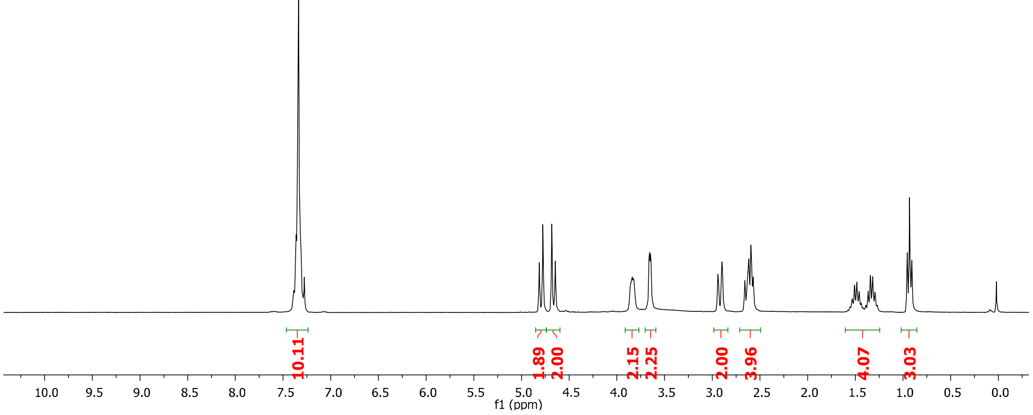

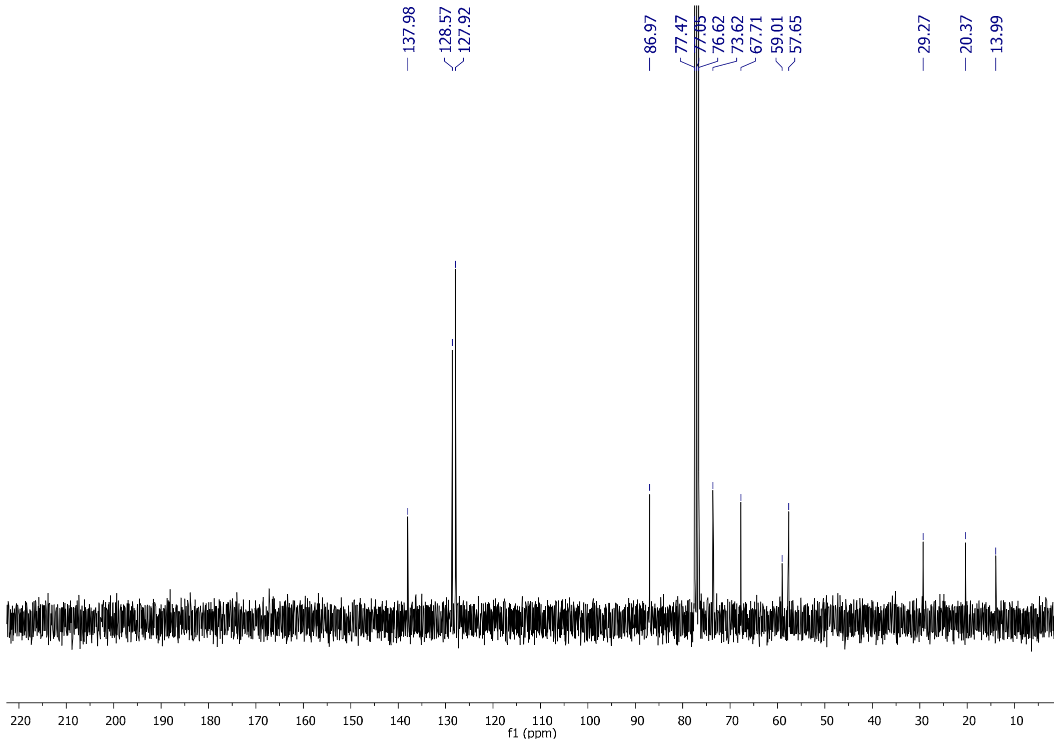

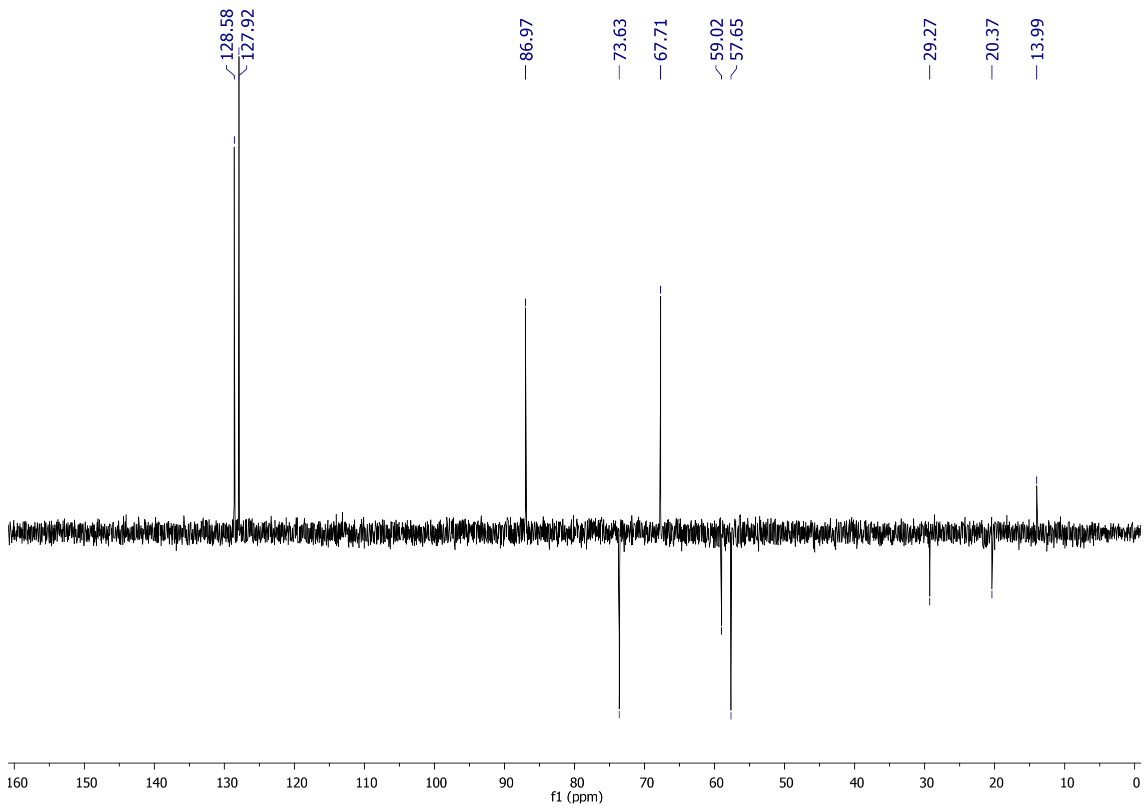
128.58
127.92
86.97
73.63
67.71
59.02
57.65
29.27
20.37
13.99
f1 (ppm)

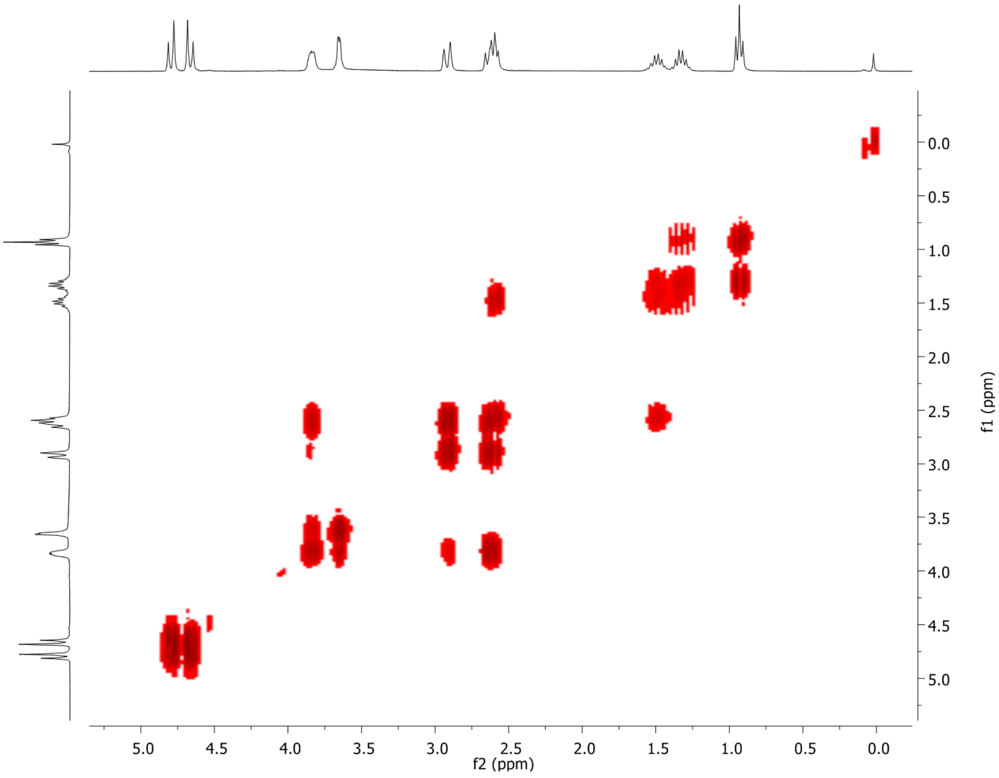
f2 (ppm)
f1 (ppm)

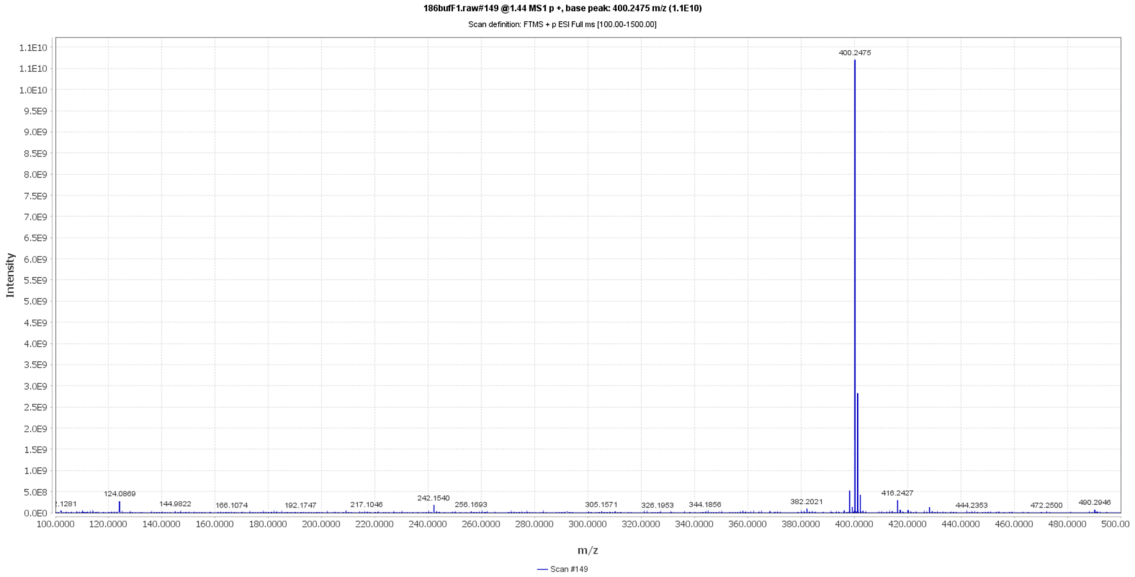

ESI HRMS: $[M+H]^+$ calcd for $C_{24}H_{34}NO_4$ 400.2483; found 400.2475

_N_-Hydroxyethyl-2,3,4,6-tetra-_O_-acetyl-1,5-dideoxy-1,5-imino-D-glucitol (38a):

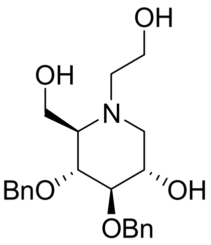

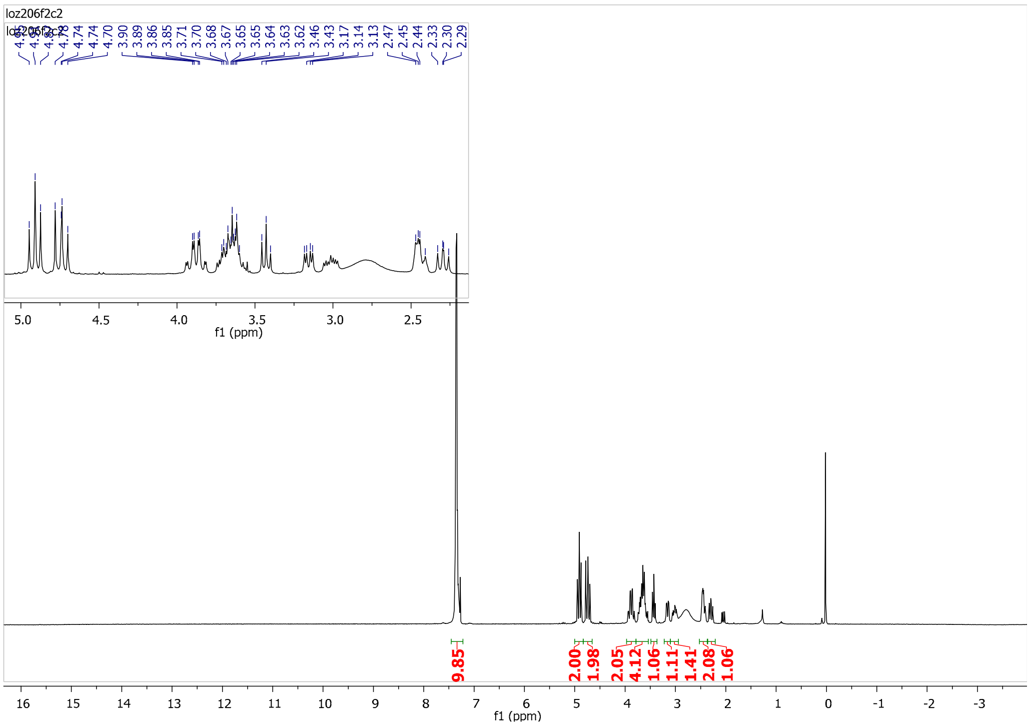

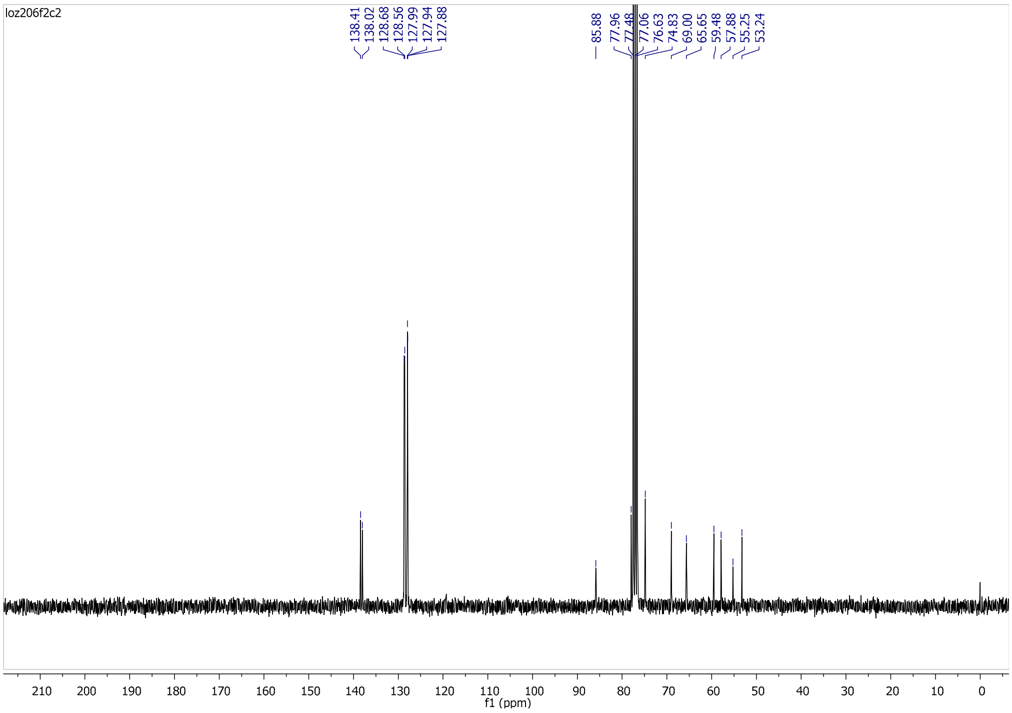

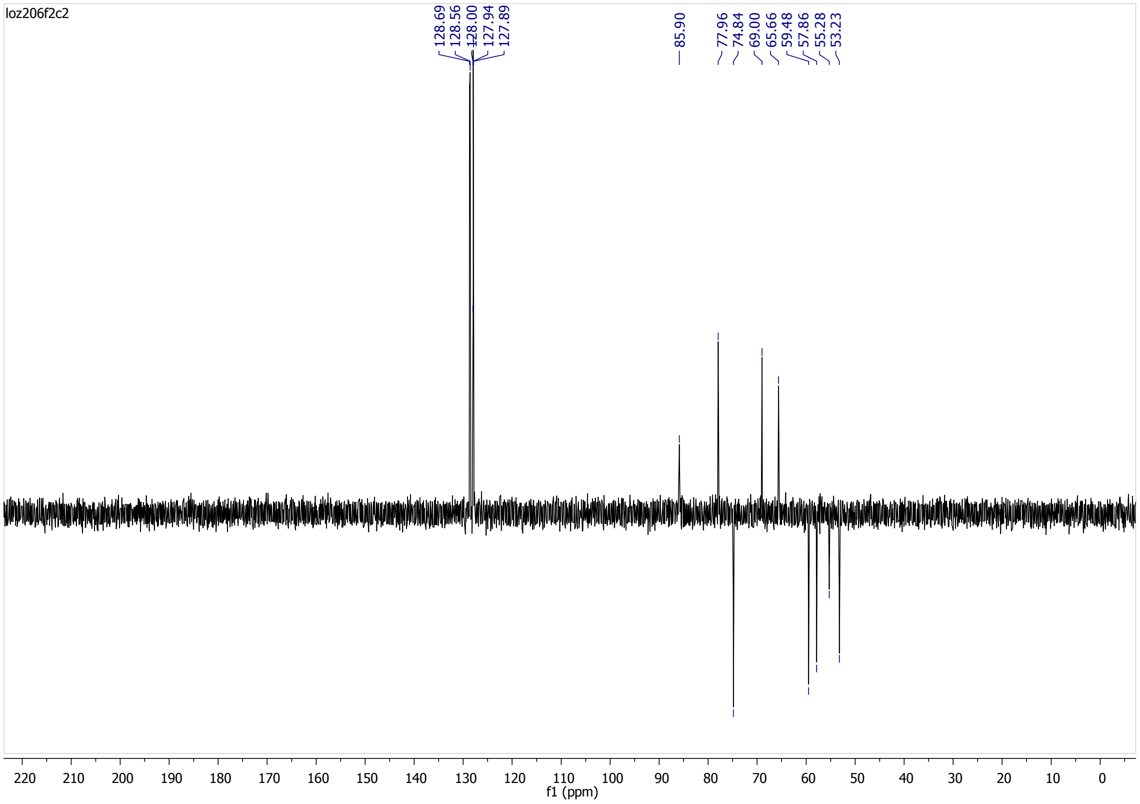
loz206f2c2
128.69
128.56
128.00
127.94
127.89
85.90
77.96
74.84
69.00
65.66
59.48
57.86
55.28
53.23
220 210 200 190 180 170 160 150 140 130 120 110 100 90 80 70 60 50 40 30 20 10 0
f1 (ppm)

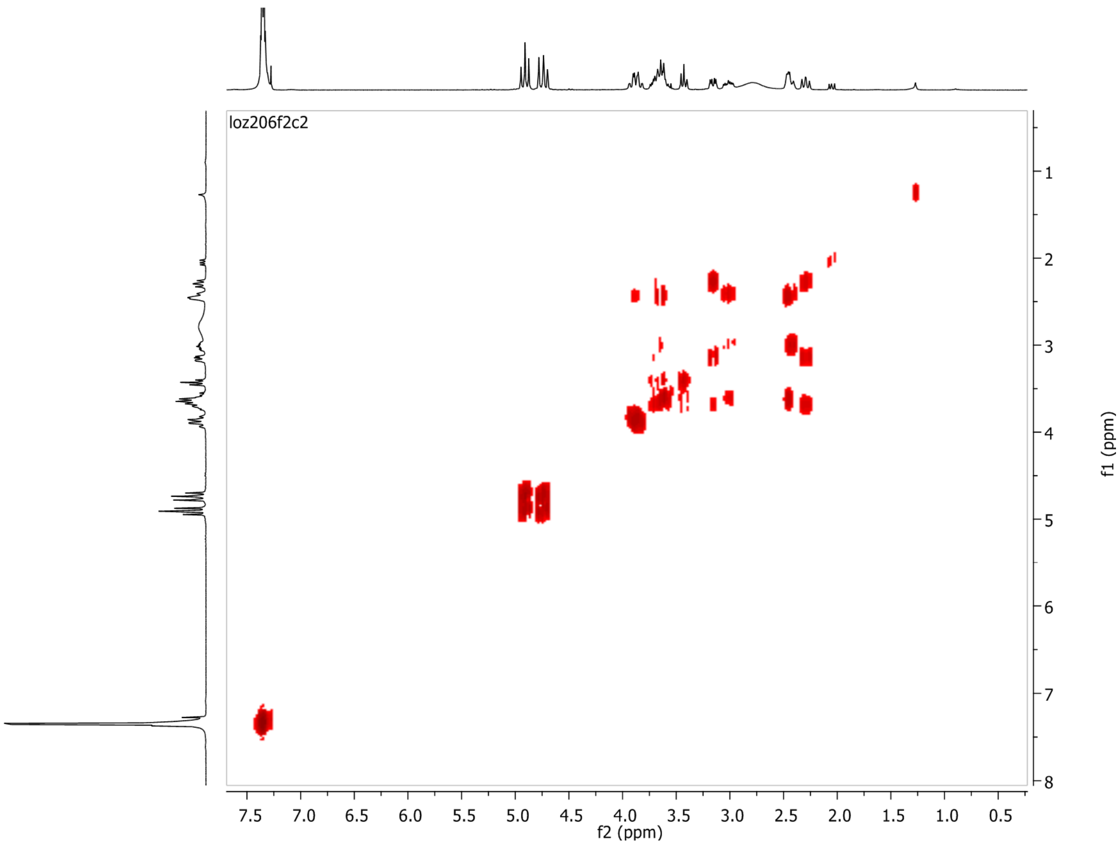
loz206f2c2
7.5 7.0 6.5 6.0 5.5 5.0 4.5 4.0 3.5 3.0 2.5 2.0 1.5 1.0 0.5
f2 (ppm)
1 2 3 4 5 6 7 8
f1 (ppm)

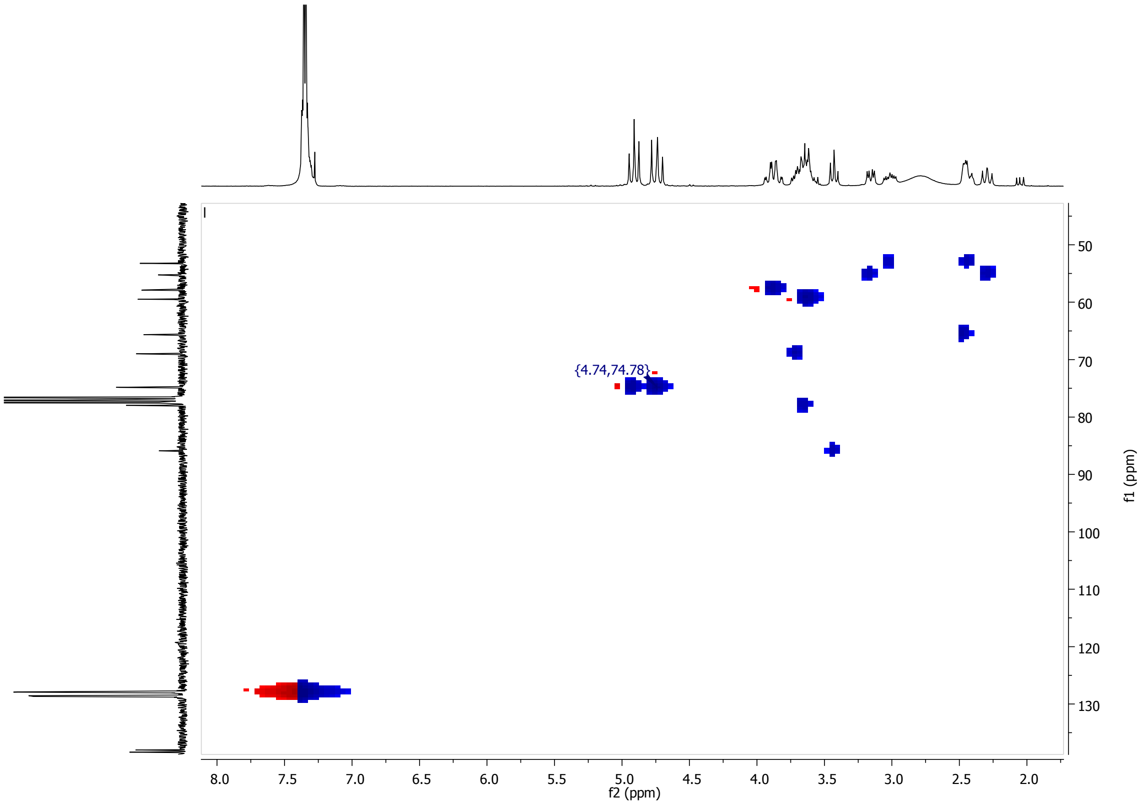

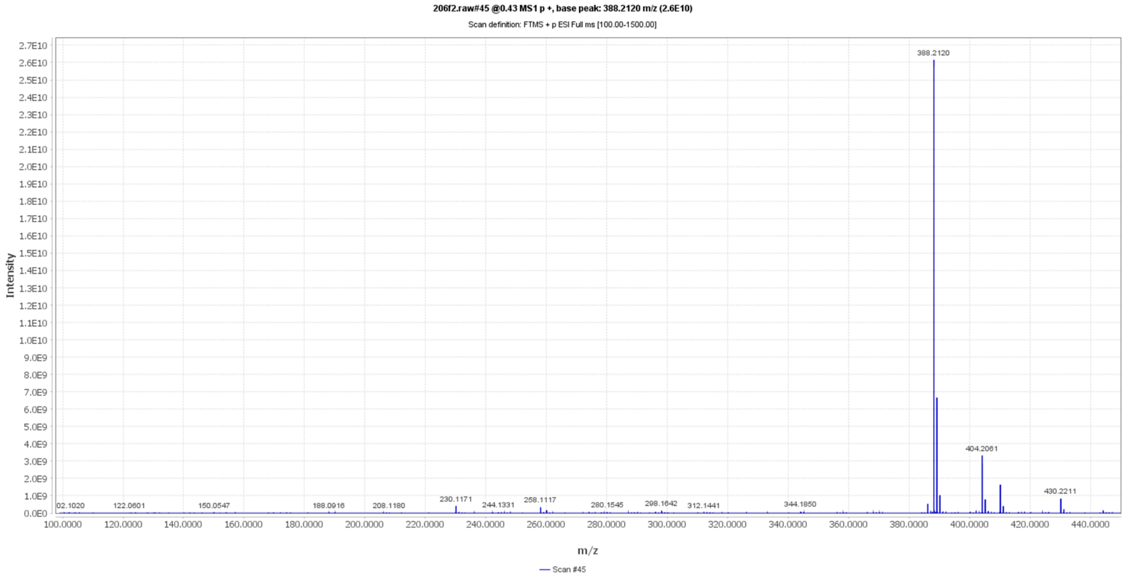

ESI HRMS: $[M+H]^+$ calcd for $C_{22}H_{30}NO_5$ 388.2119; found 388.2120

_N_-Hydroxyethyl-2,3,4,6-tetra-_O_-acetyl-1,6-dideoxy-1,6-imino-L-iditol (38b):

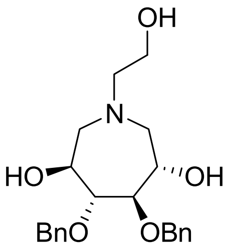

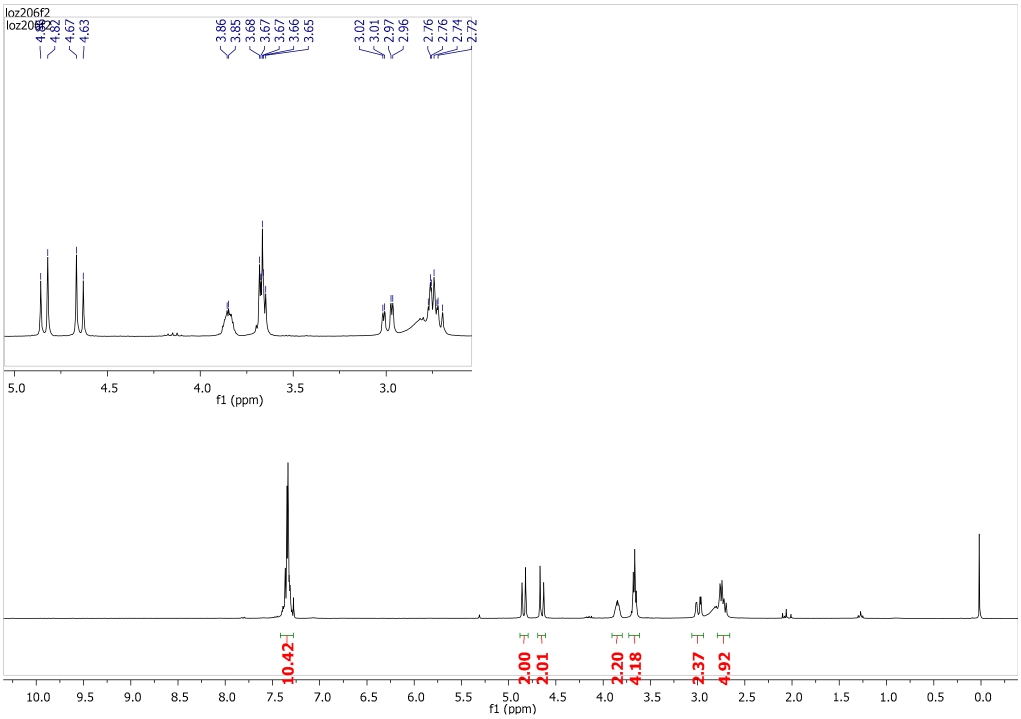

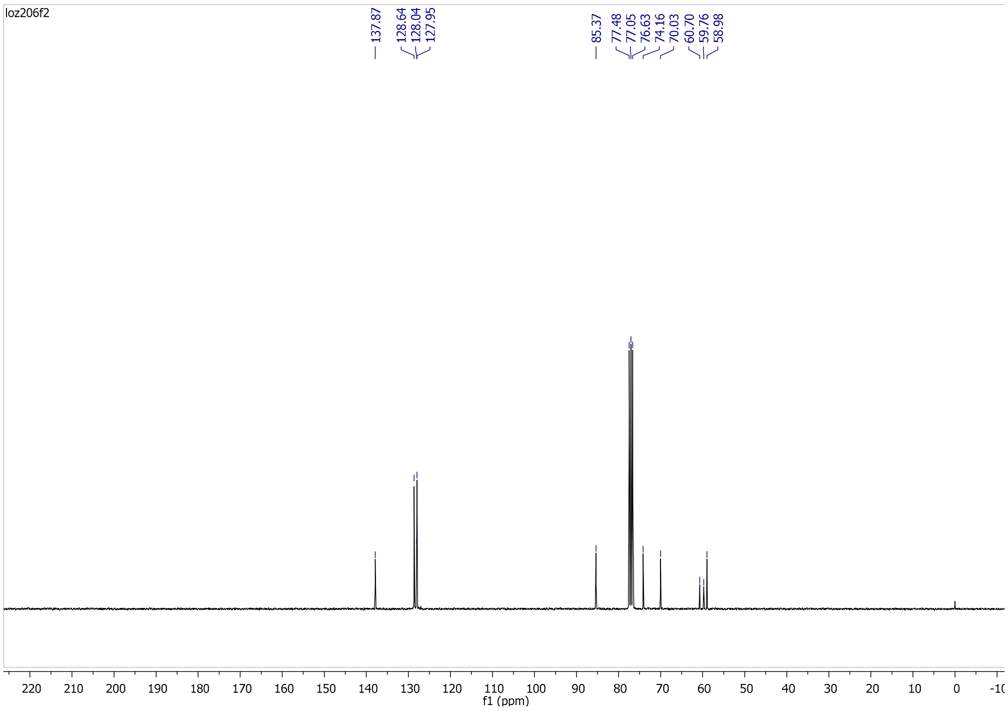

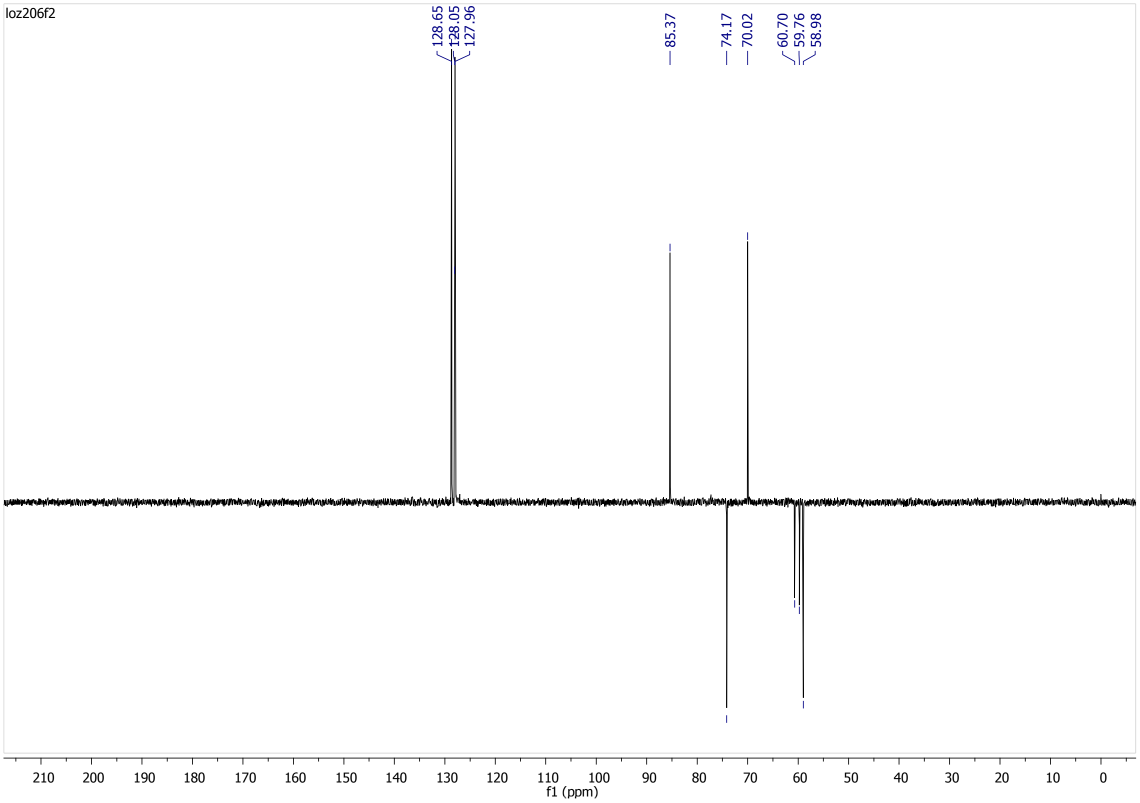
loz206f2
128.65
128.05
127.96
85.37
74.17
70.02
60.70
59.76
58.98
210 200 190 180 170 160 150 140 130 120 110 100 90 80 70 60 50 40 30 20 10 0
f1 (ppm)

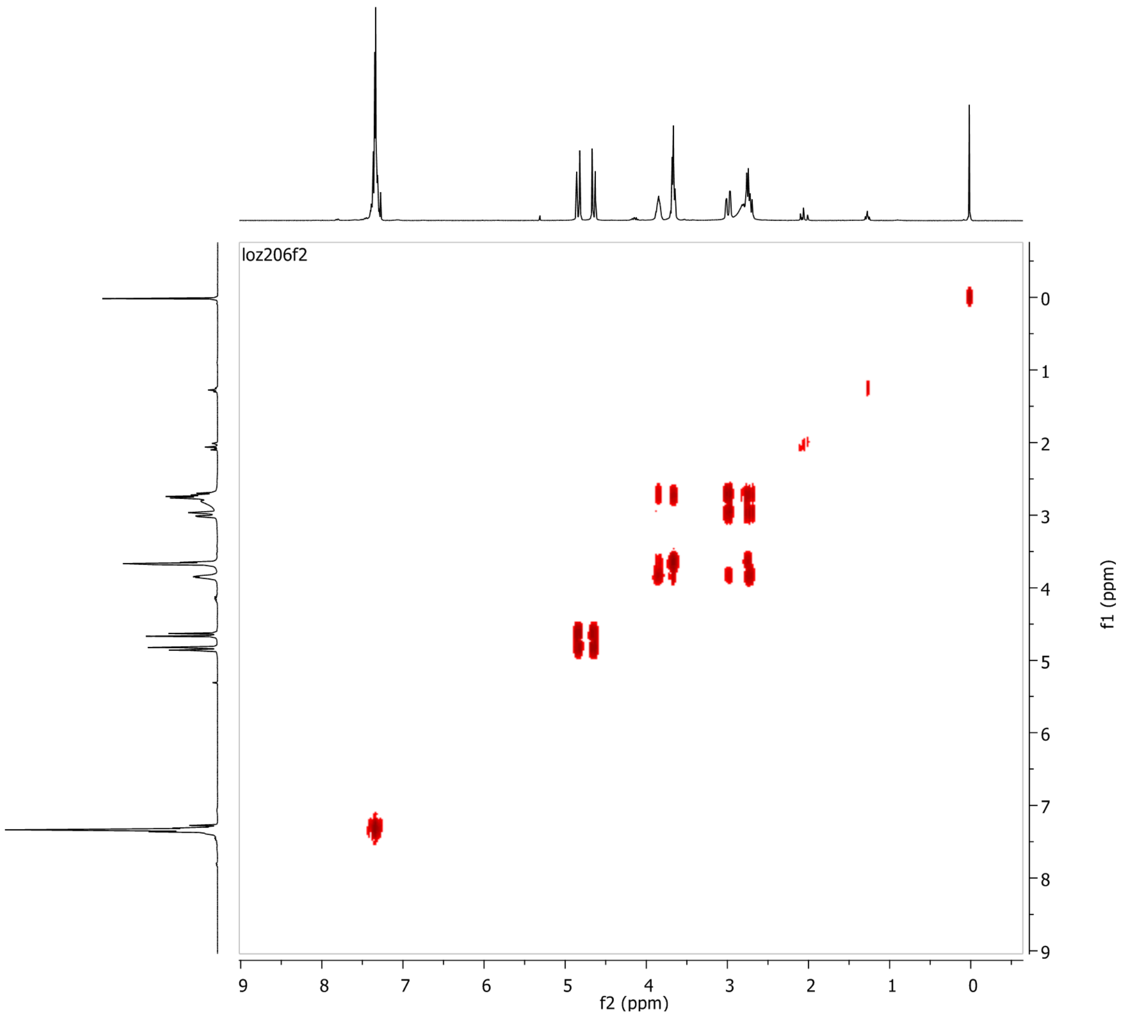
loz206f2
0 1 2 3 4 5 6 7 8 9
f1 (ppm)
9 8 7 6 5 4 3 2 1 0
f2 (ppm)

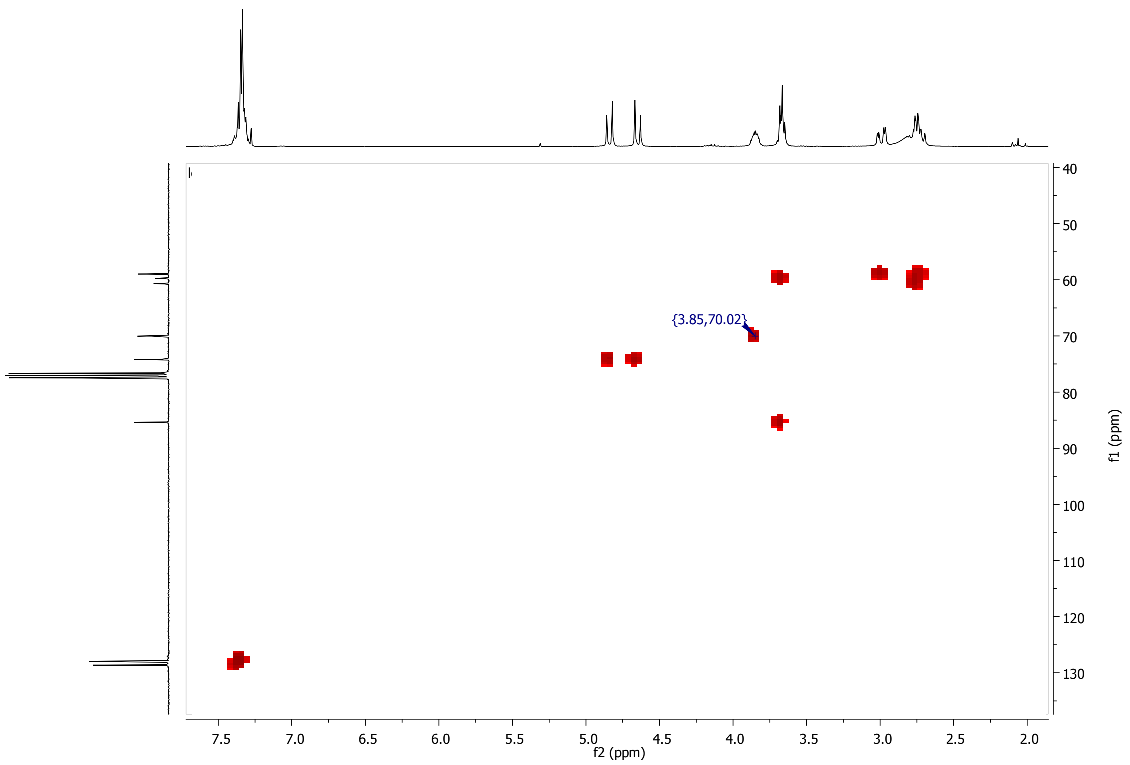

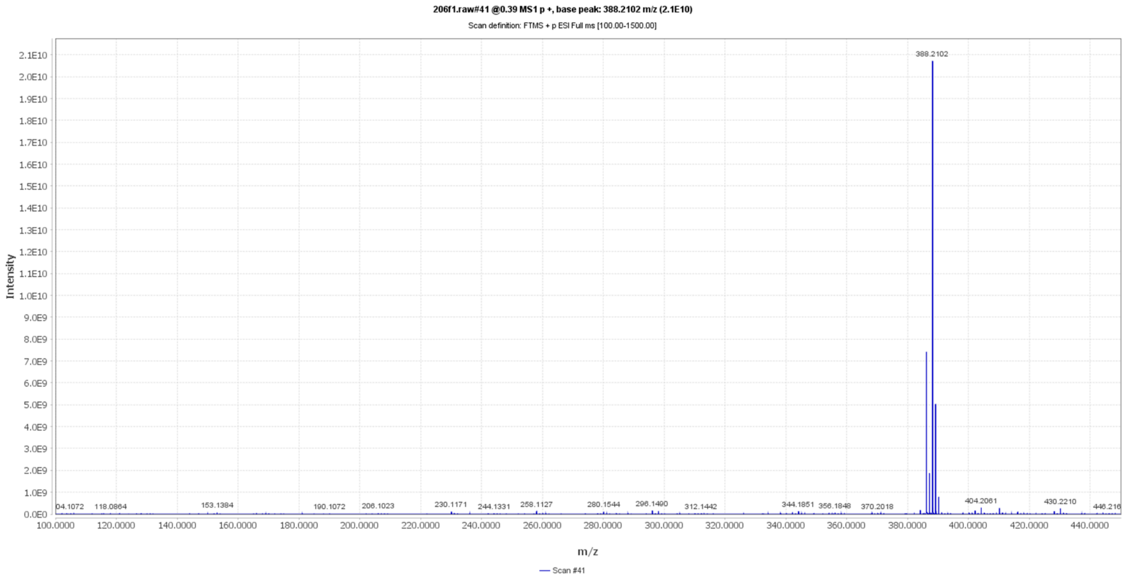

ESI HRMS: $[M+H]^+$ calcd for $C_{22}H_{30}NO_5$ 388.2119; found 388.2102

N-Propynyl-1,5-dideoxy-1,5-imino-D-glucitol (39a):

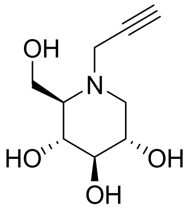

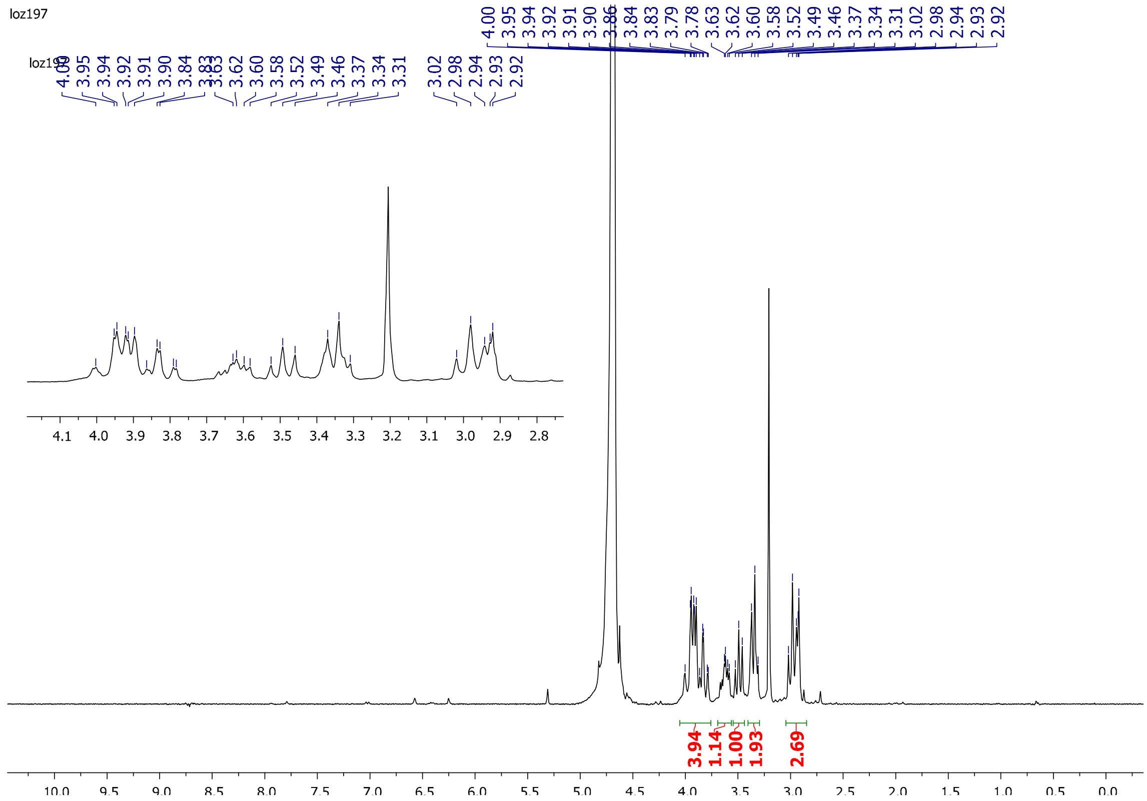

***N*-Propynyl-1,6-dideoxy-1,6-imino-L-iditol (39b):**

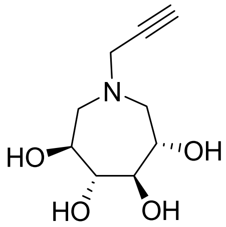

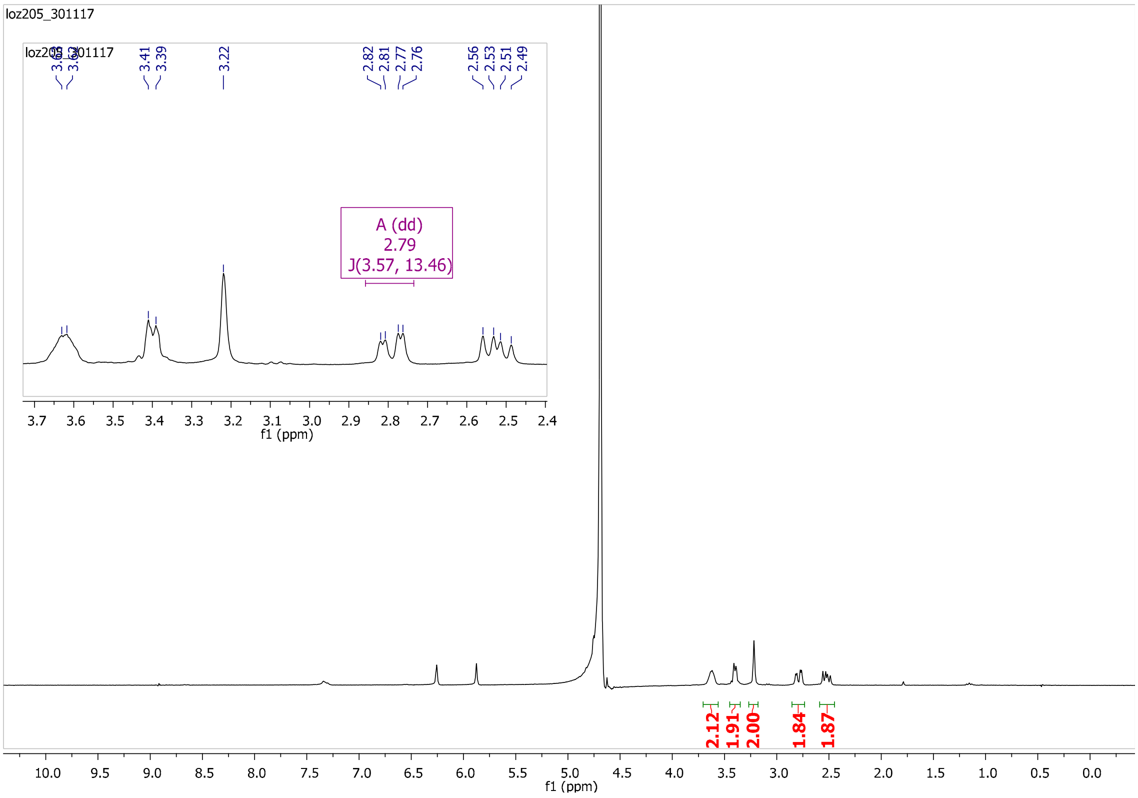

_N_-Butyl-1,5-dideoxy-1,5-imino-D-glucitol (40a):

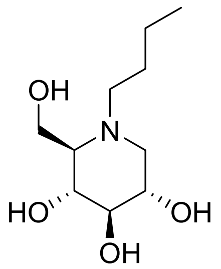

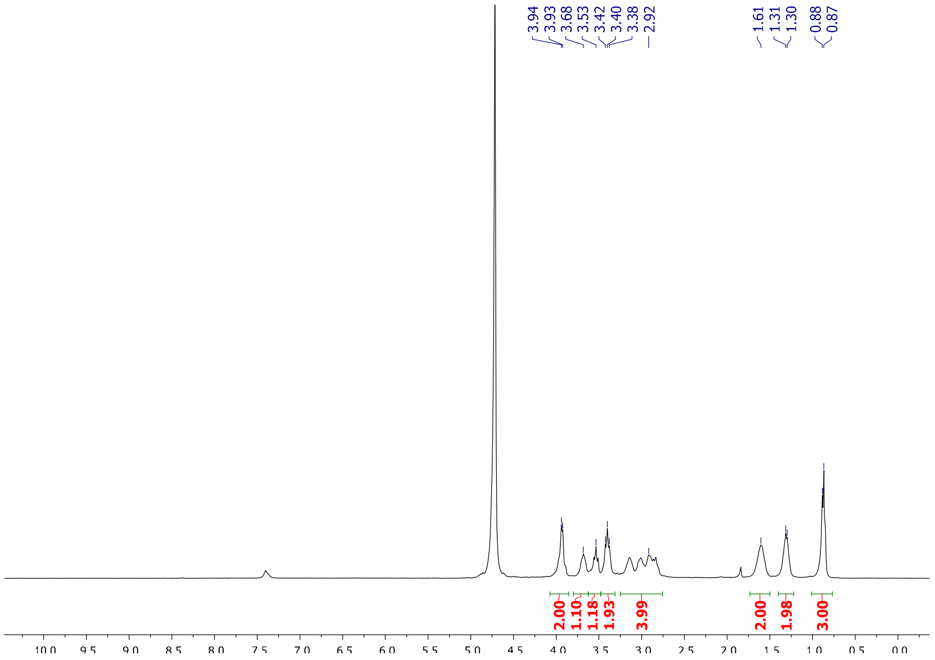

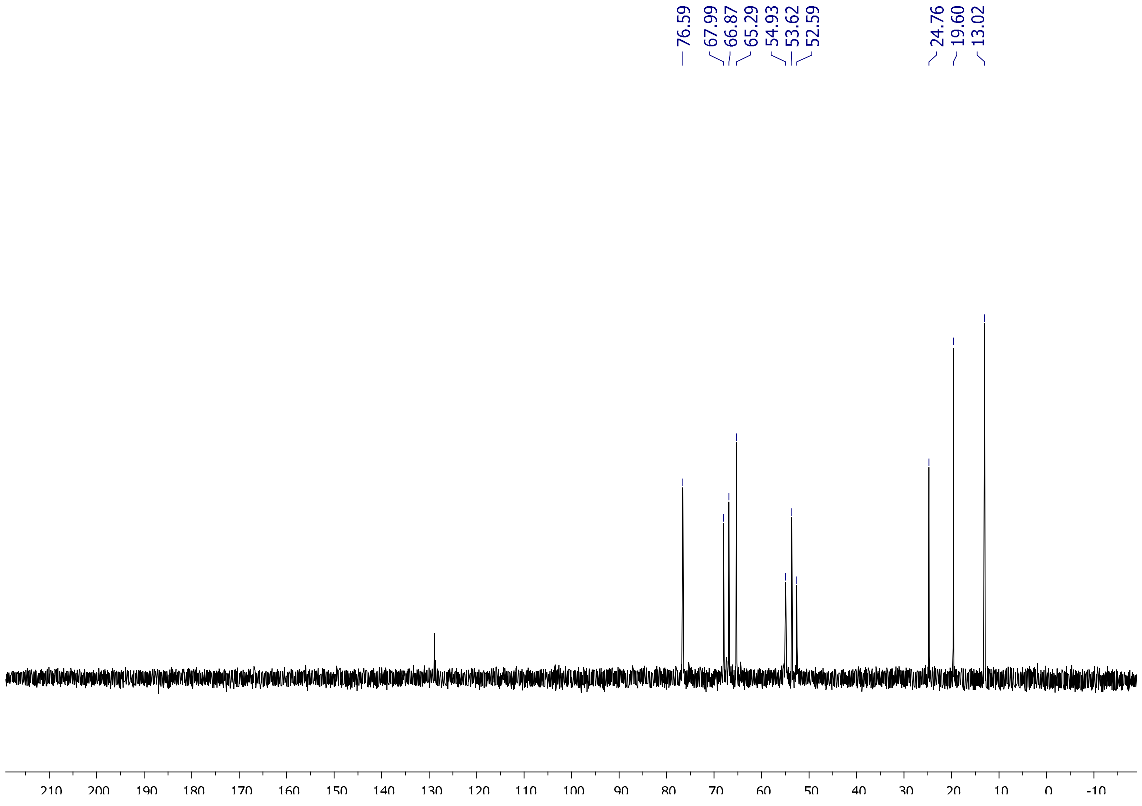

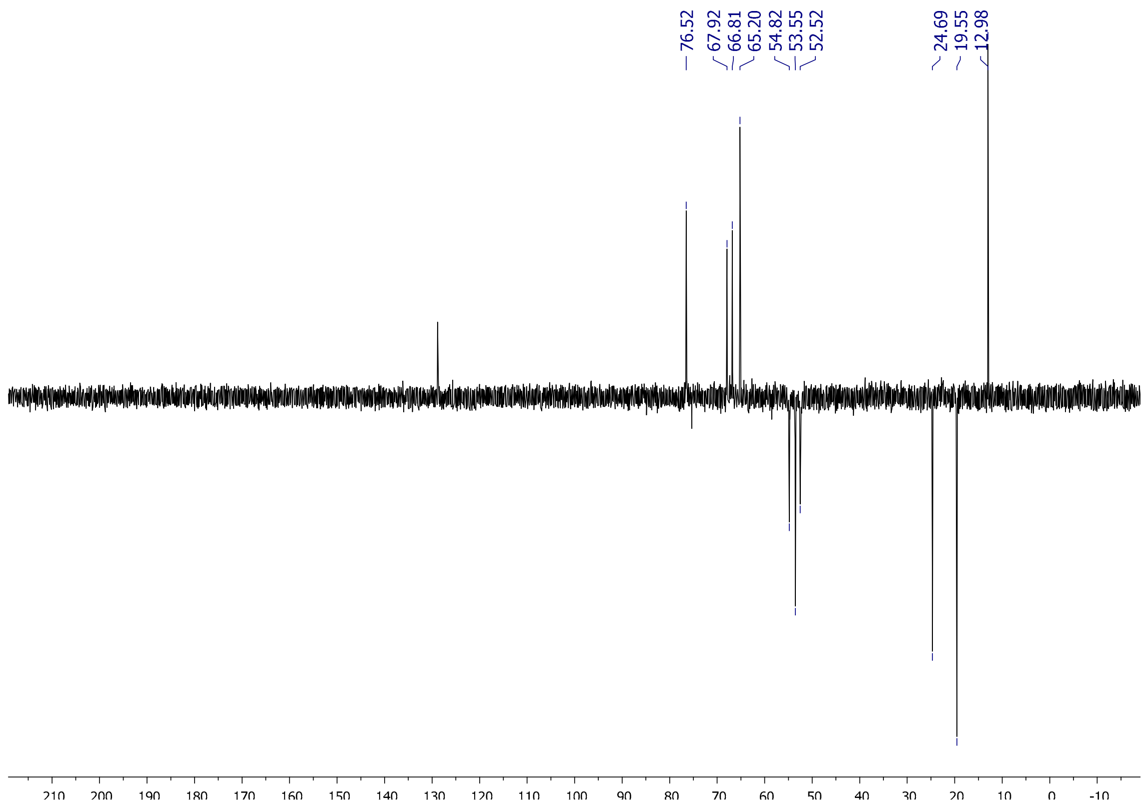
76.52
67.92
66.81
65.20
54.82
53.55
52.52
24.69
19.55
12.98
210 200 190 180 170 160 150 140 130 120 110 100 90 80 70 60 50 40 30 20 10 0 -10

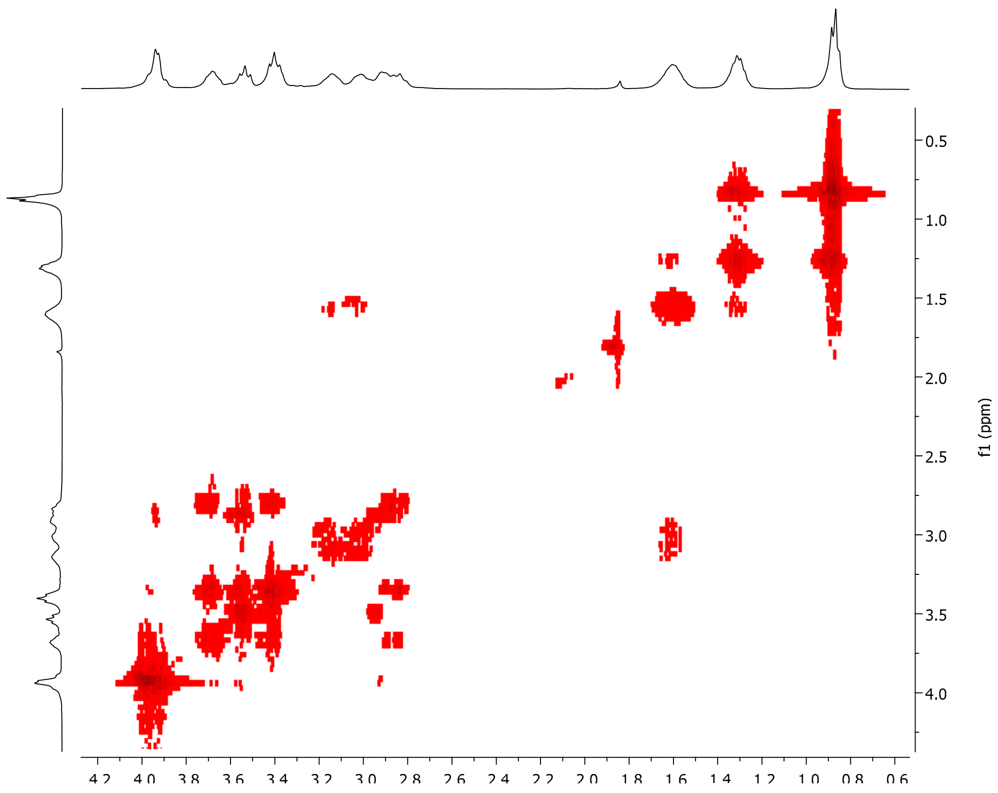
f1 (ppm)
0.5 1.0 1.5 2.0 2.5 3.0 3.5 4.0
4.2 4.0 3.8 3.6 3.4 3.2 3.0 2.8 2.6 2.4 2.2 2.0 1.8 1.6 1.4 1.2 1.0 0.8 0.6

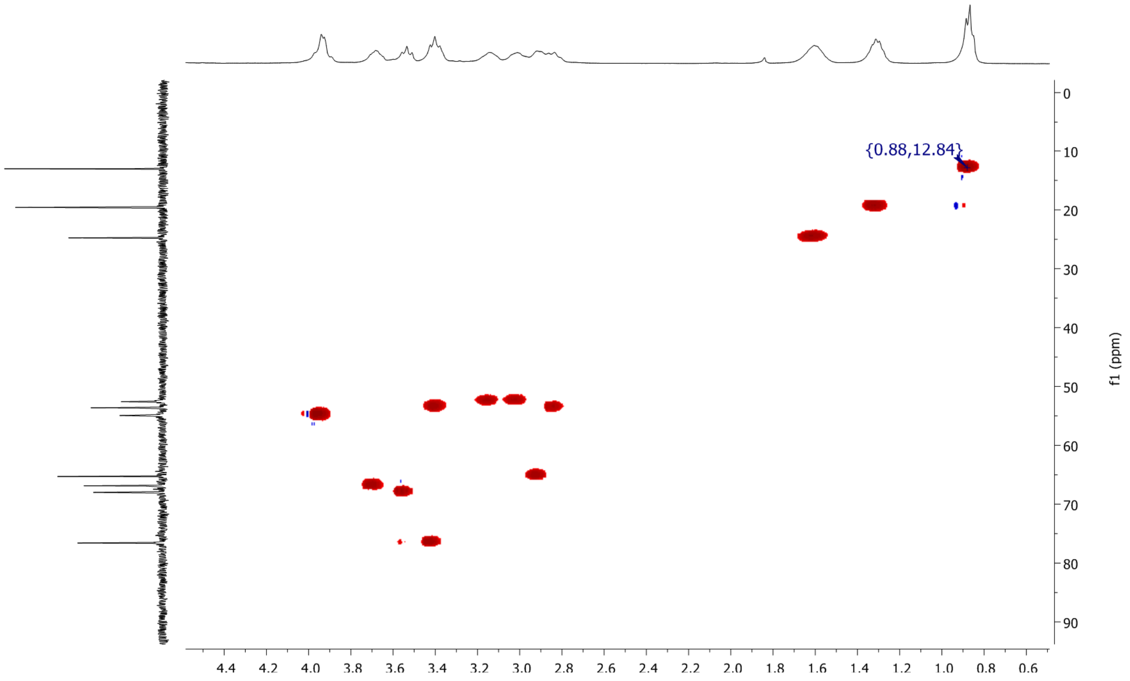
{0.88,12.84}
f1 (ppm)

***N*-Butyl-1,6-dideoxy-1,6-imino-L-iditol (40b):**

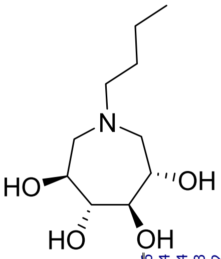

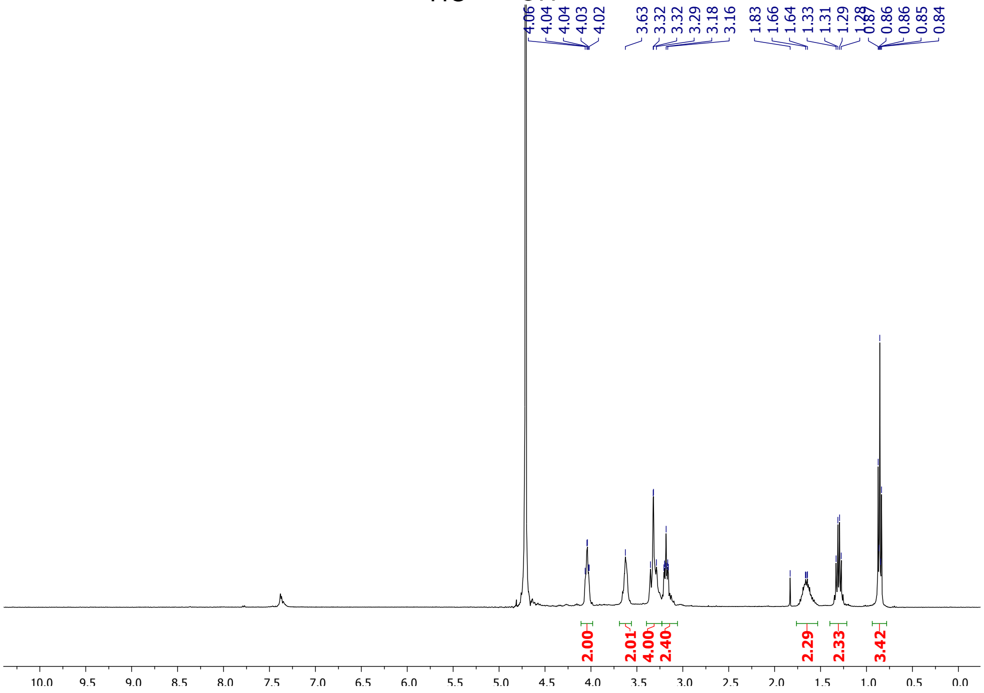

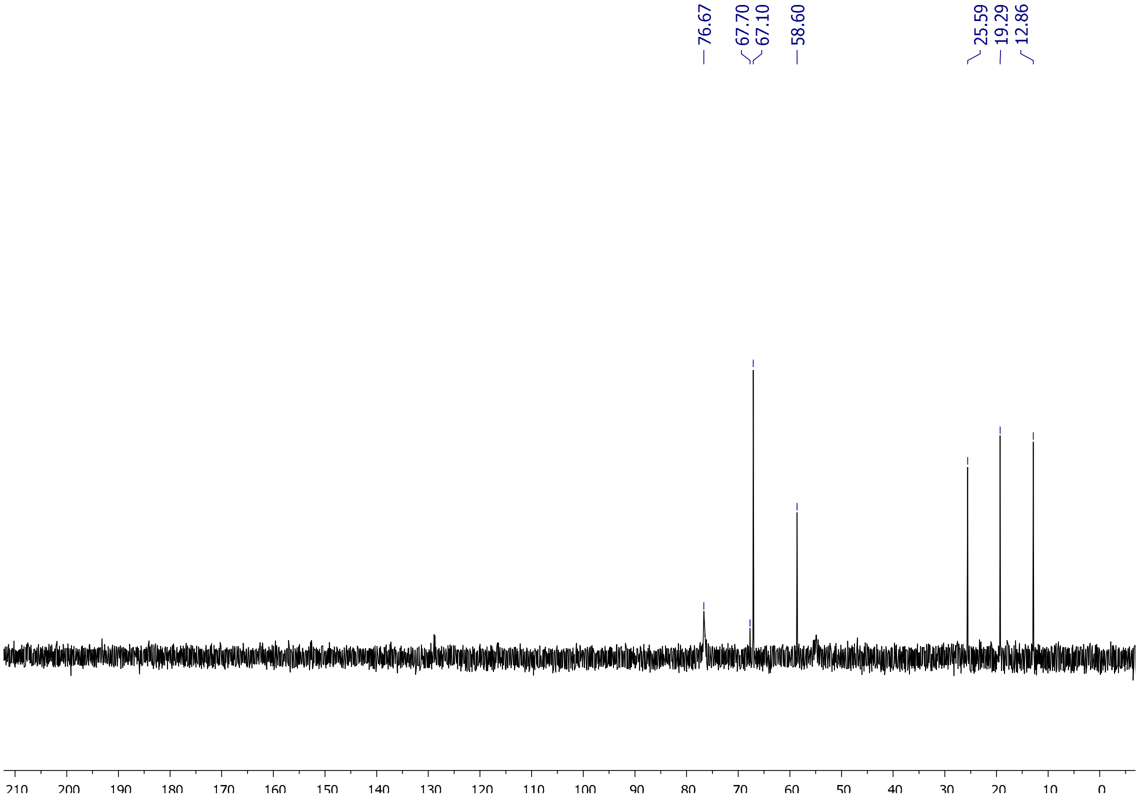

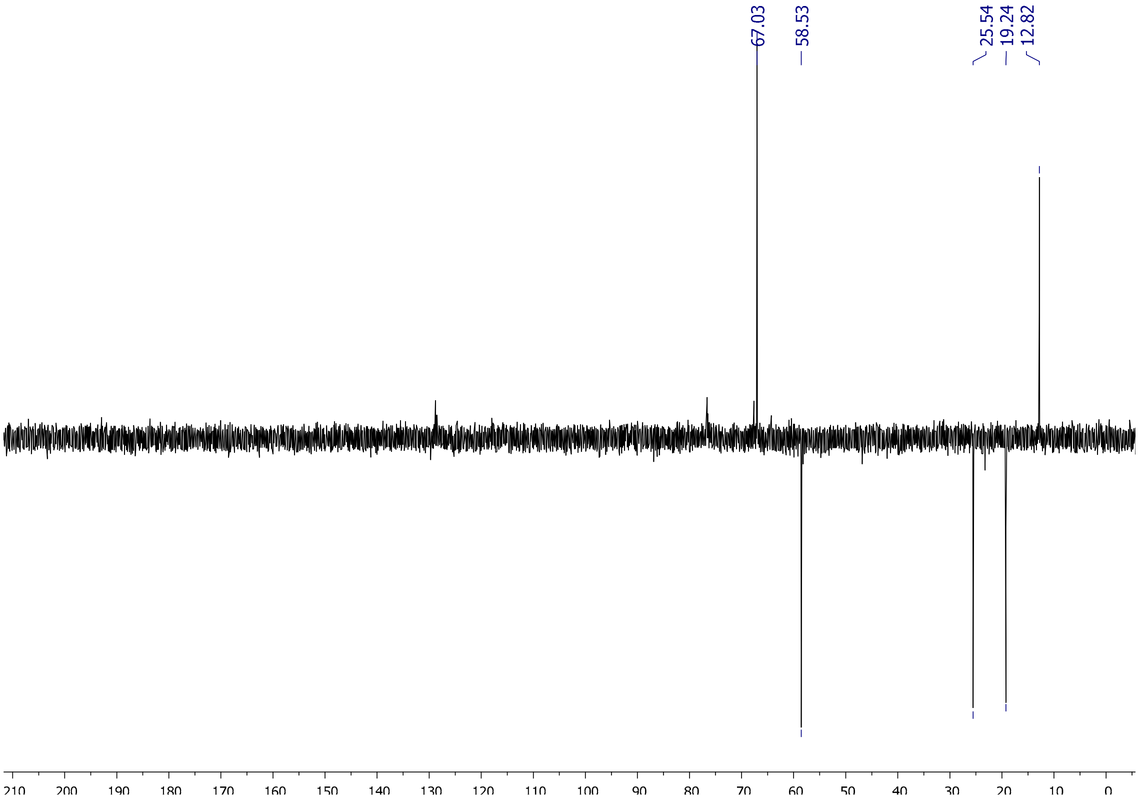
67.03
58.53
25.54
19.24
12.82
210 200 190 180 170 160 150 140 130 120 110 100 90 80 70 60 50 40 30 20 10 0

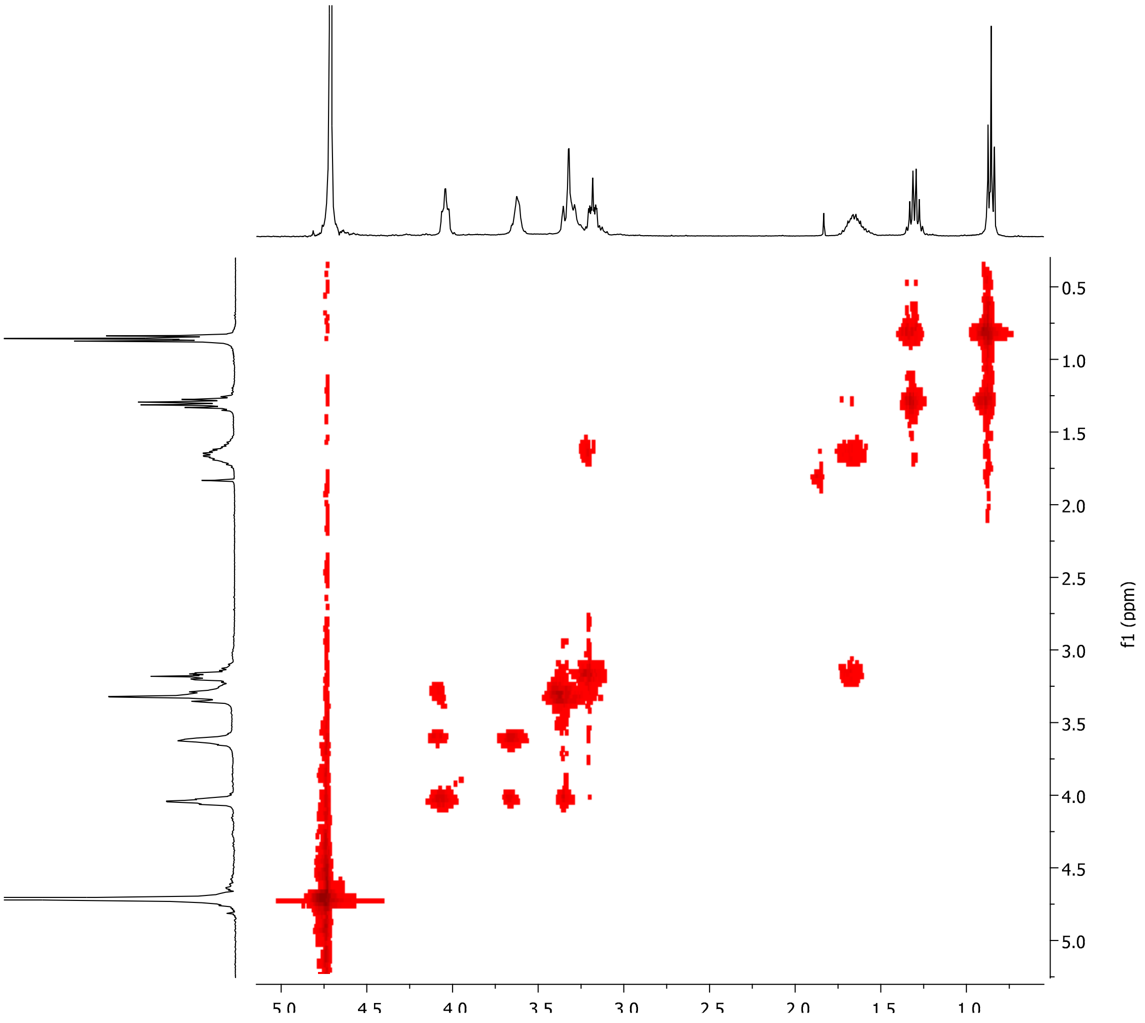
0.5
1.0
1.5
2.0
2.5
3.0
3.5
4.0
4.5
5.0
f1 (ppm)

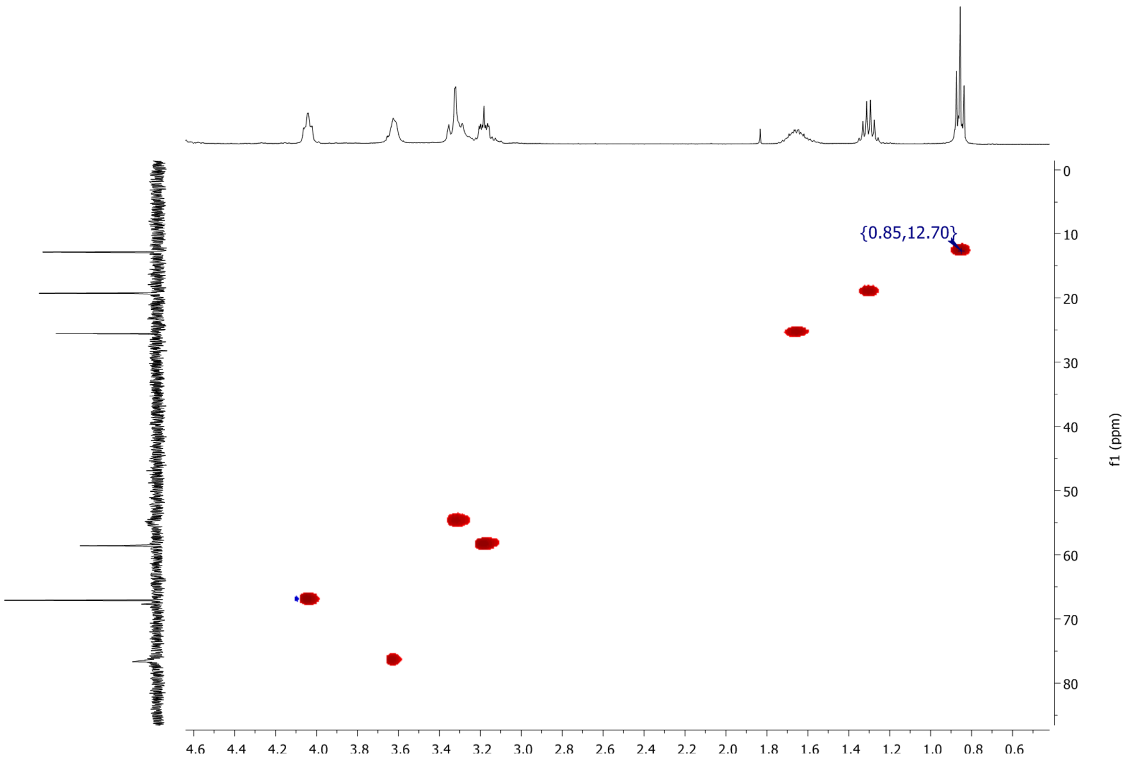

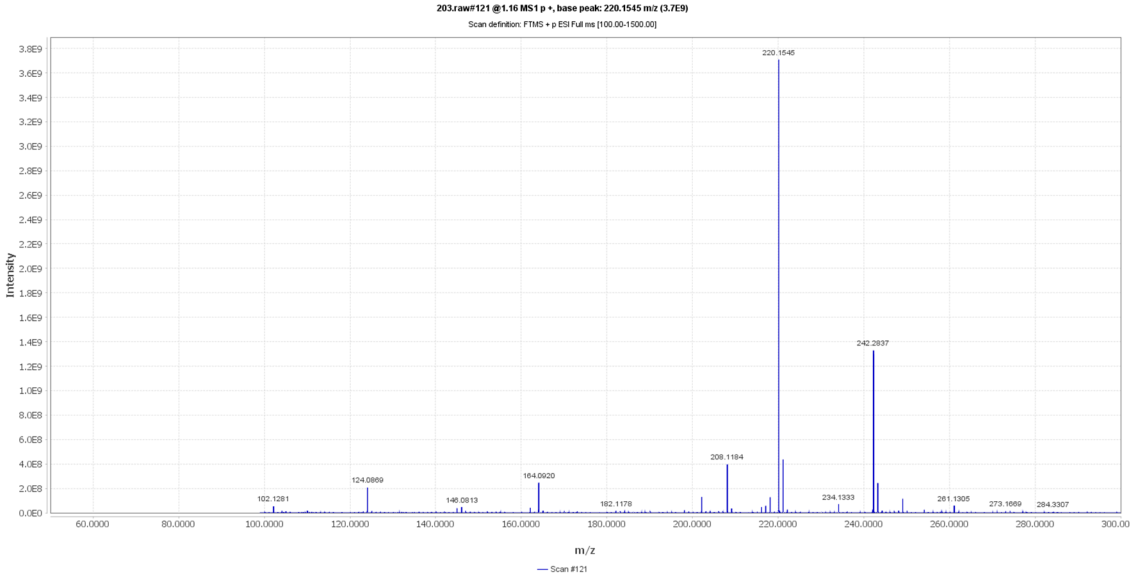

ESI HRMS: $[M+H]^+$ calcd for $C_{10}H_{22}NO_4$ 220.1544; found 220.1545

_N_-Hydroxyethyl-1,5-dideoxy-1,5-imino-D-glucitol (41a):

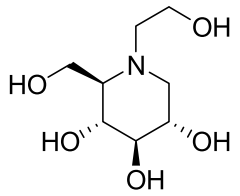

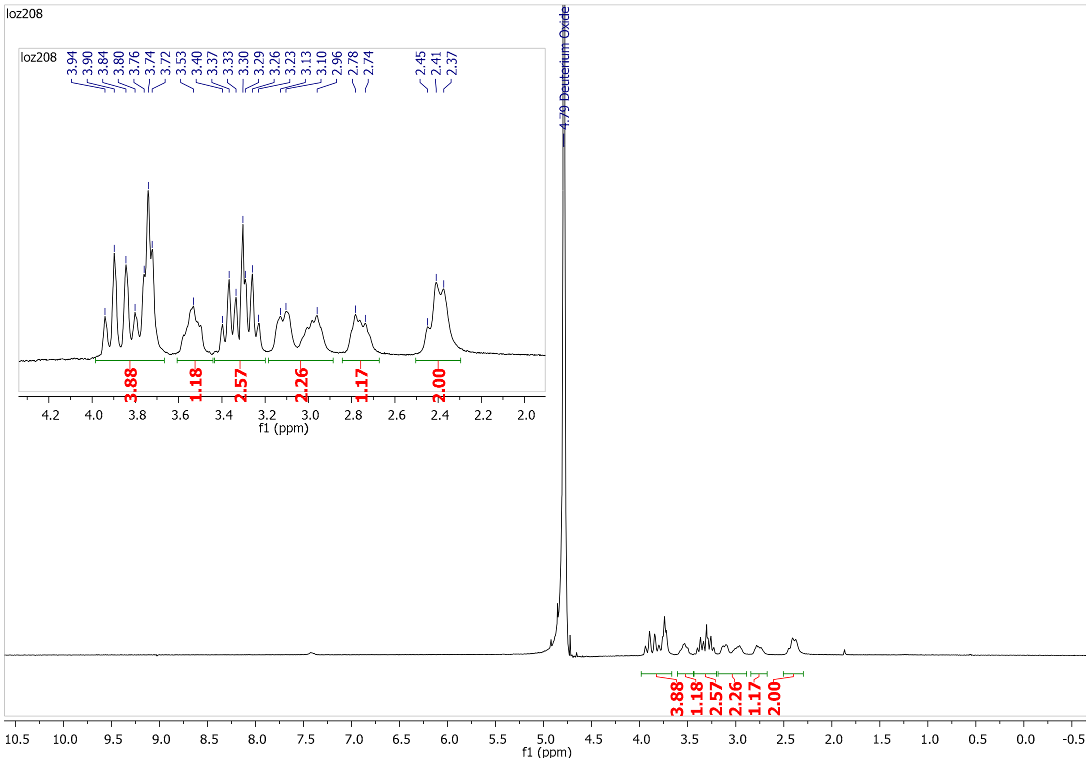

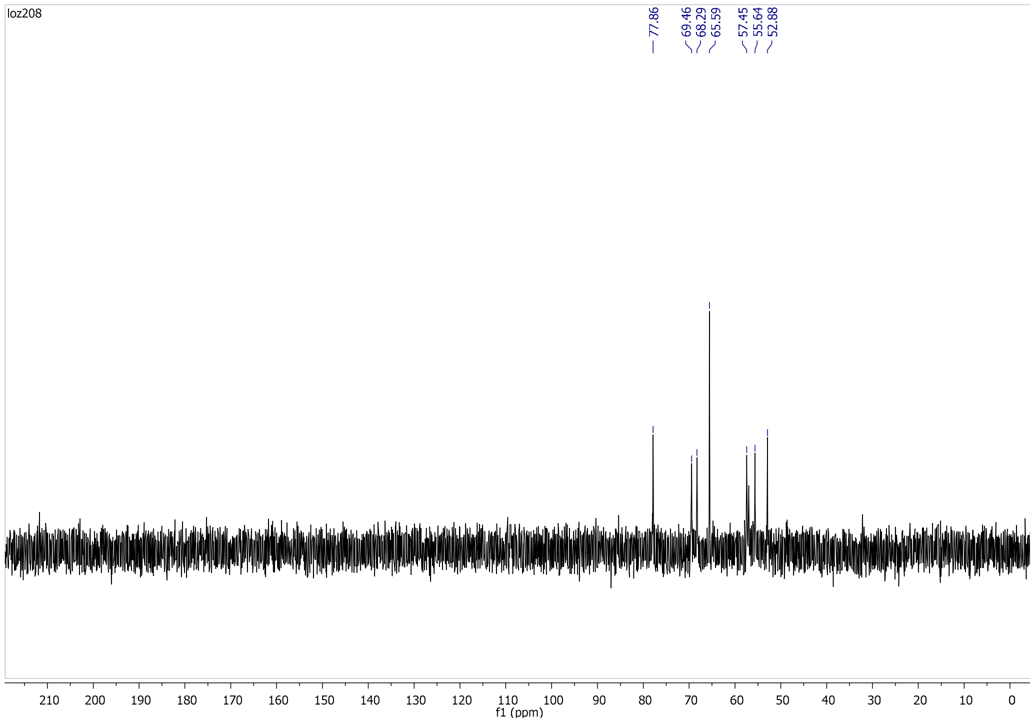

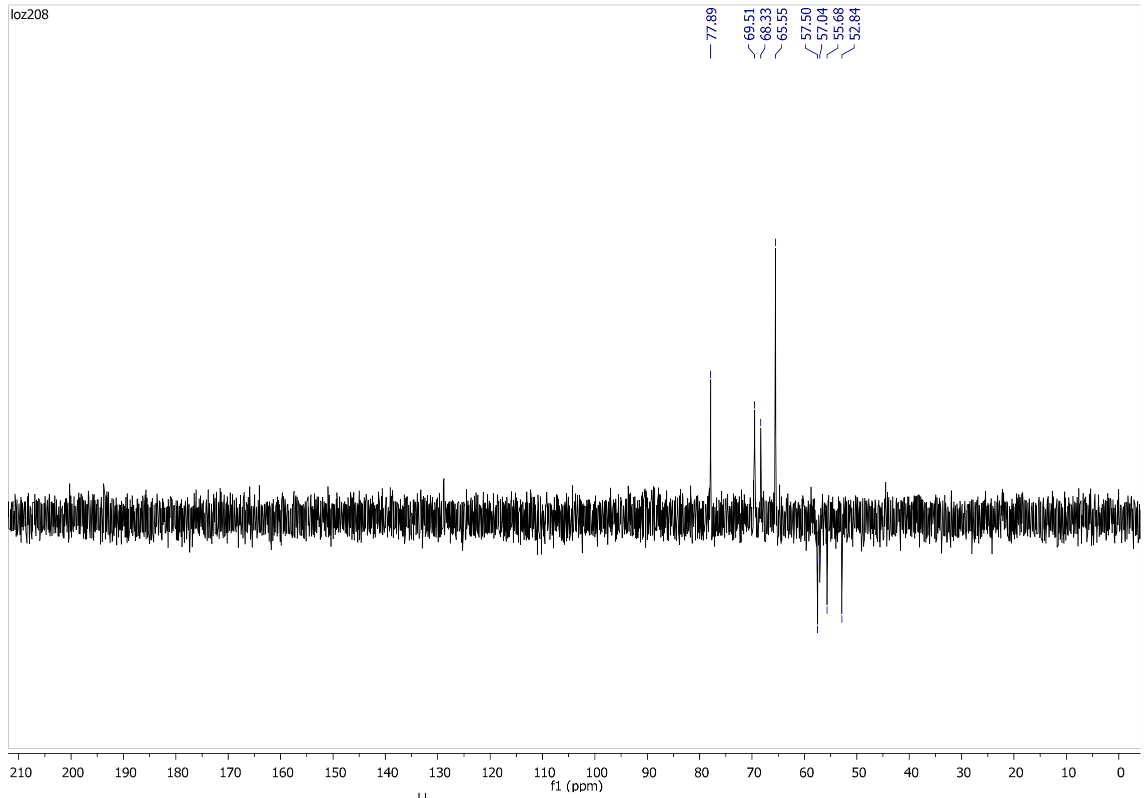
loz208
77.89
69.51
68.33
65.55
57.50
57.04
55.68
52.84
210 200 190 180 170 160 150 140 130 120 110 100 90 80 70 60 50 40 30 20 10 0
f1 (ppm)

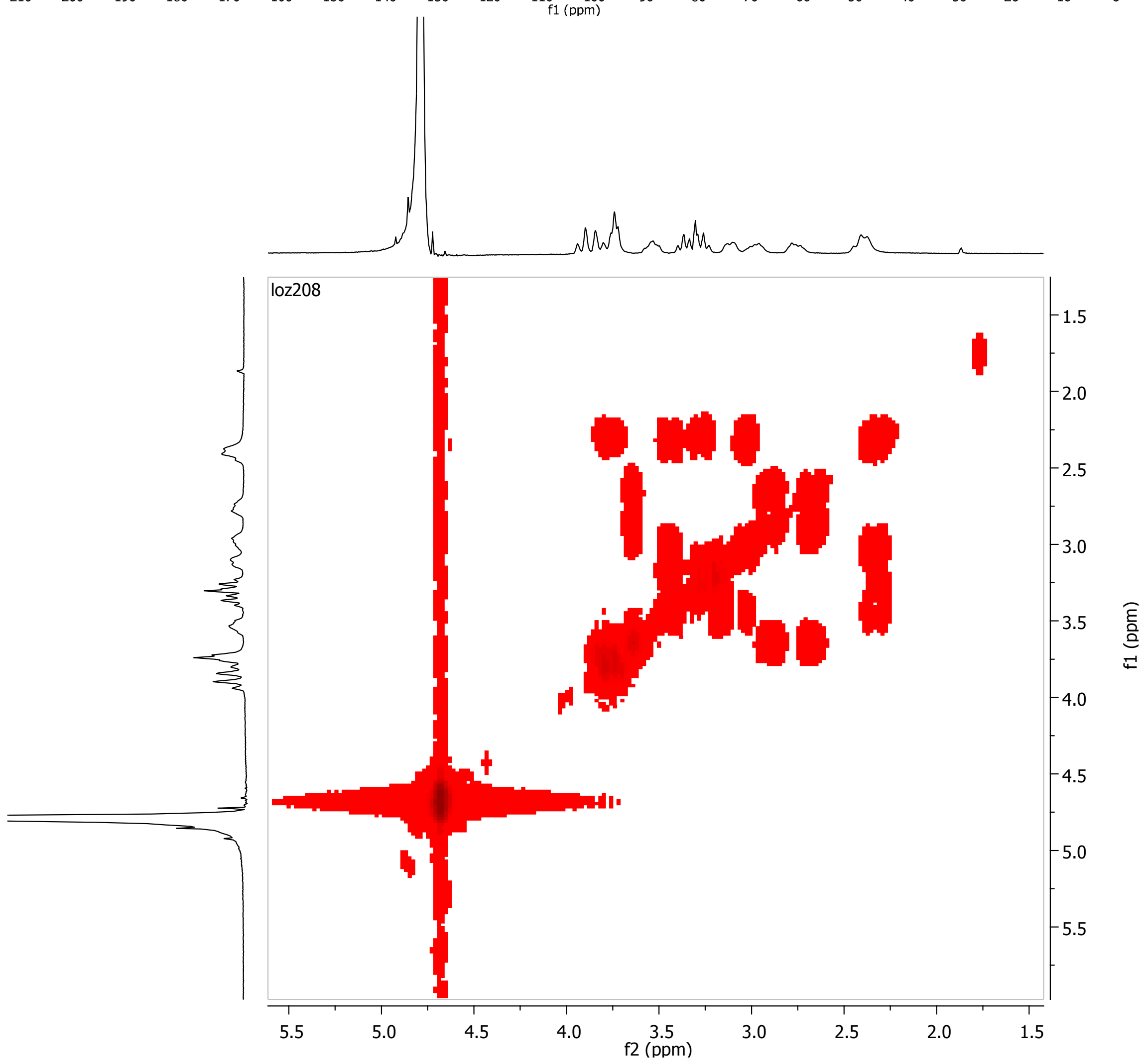
loz208
5.5 5.0 4.5 4.0 3.5 3.0 2.5 2.0 1.5
f2 (ppm)
f1 (ppm)

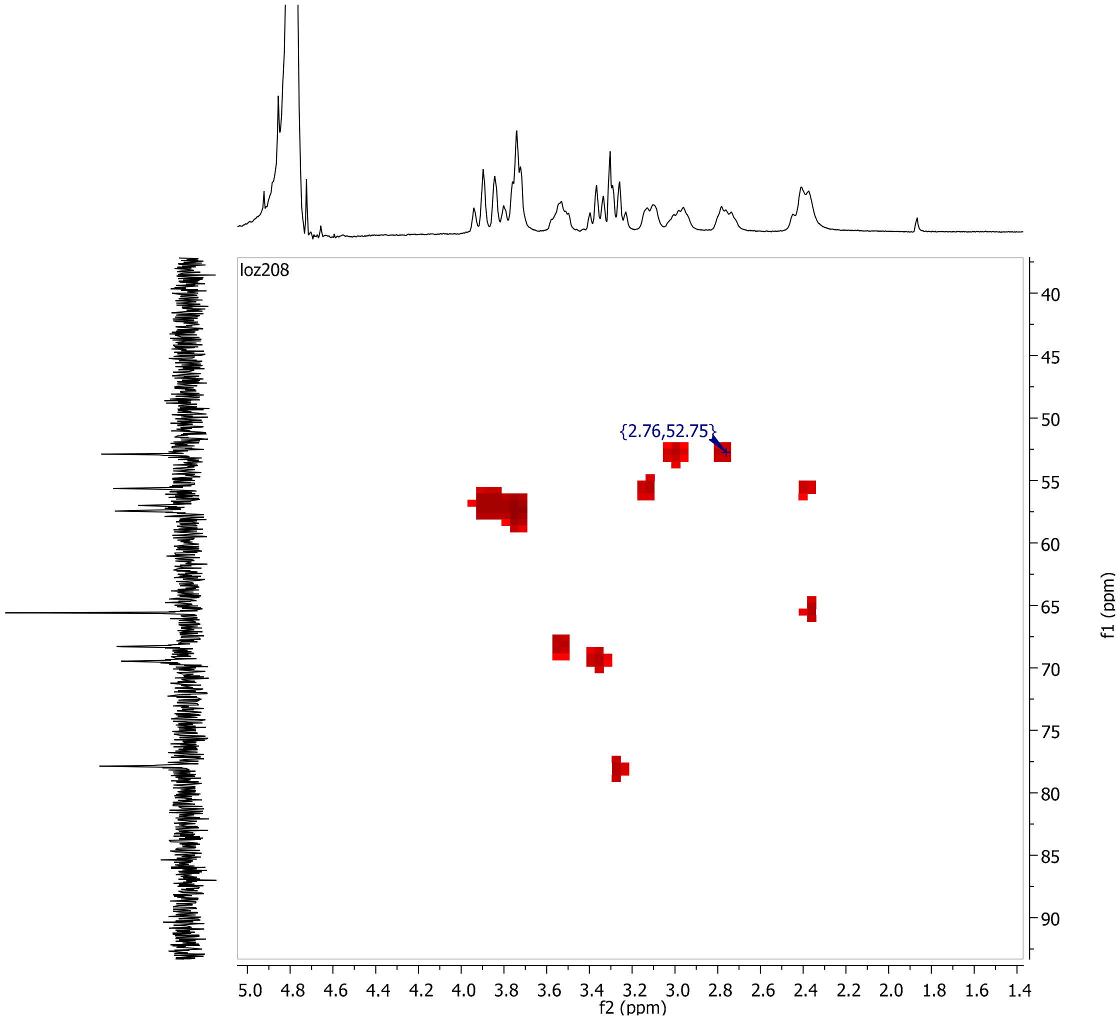
loz208
{2.76,52.75}
f1 (ppm)
f2 (ppm)
5.0 4.8 4.6 4.4 4.2 4.0 3.8 3.6 3.4 3.2 3.0 2.8 2.6 2.4 2.2 2.0 1.8 1.6 1.4
40 45 50 55 60 65 70 75 80 85 90

N-Hydroxyethyl-1,6-dideoxy-1,6-imino-L-iditol (41b):

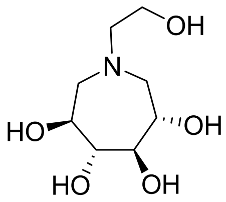

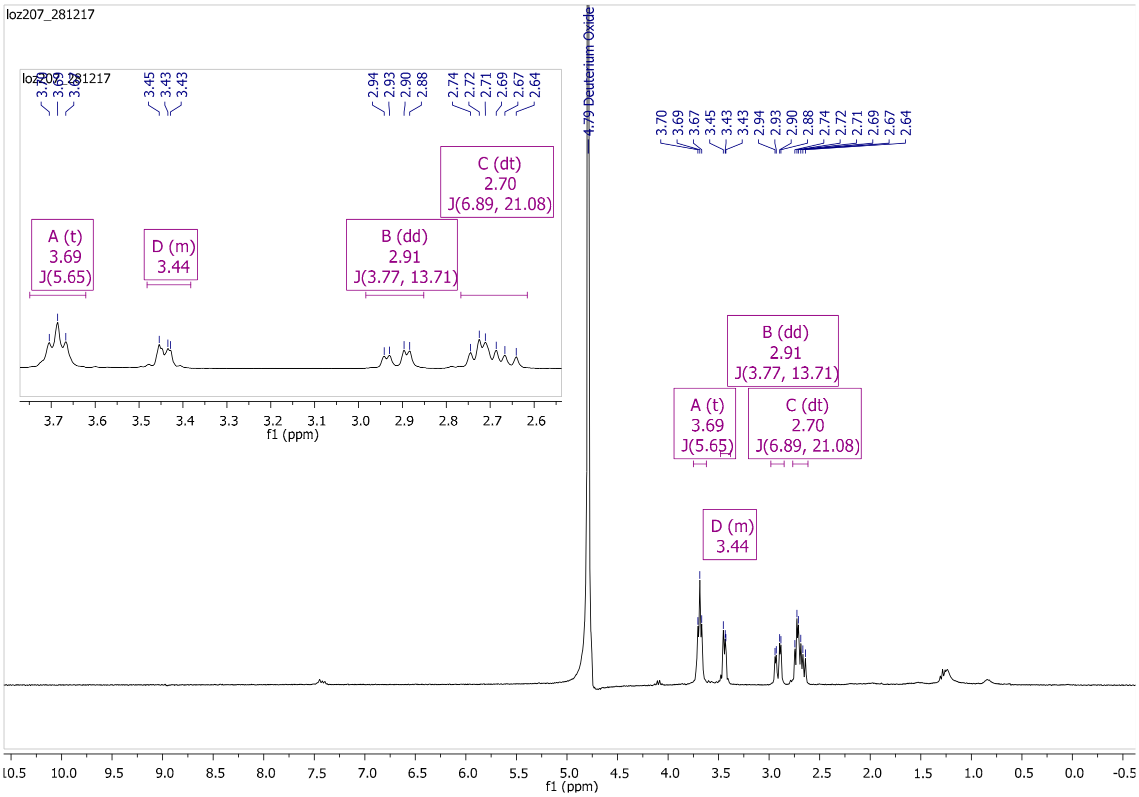

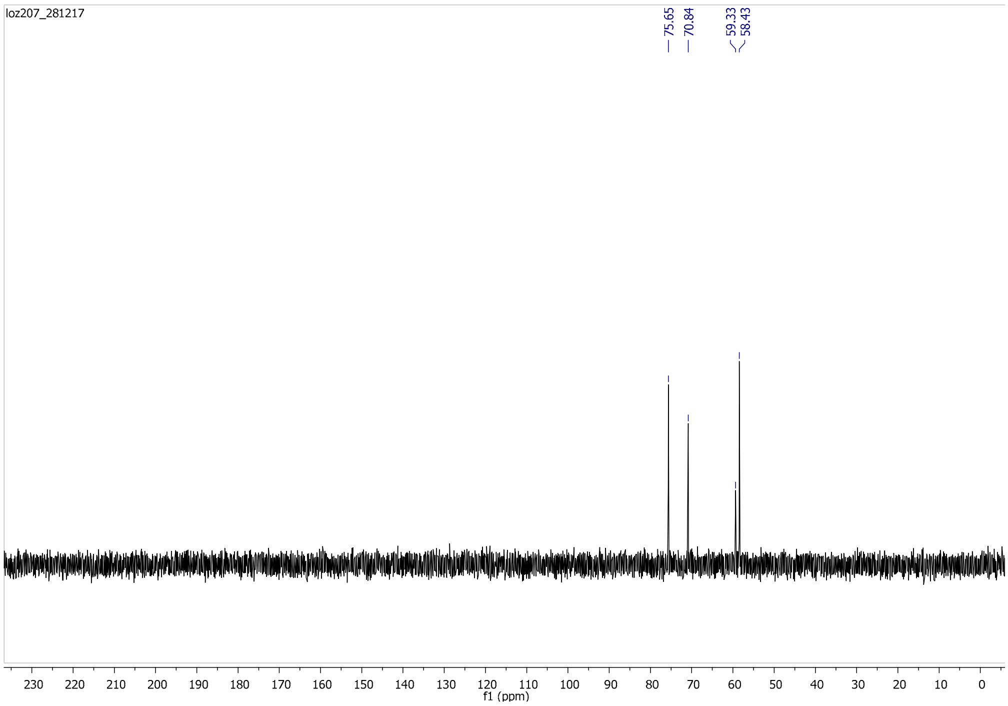

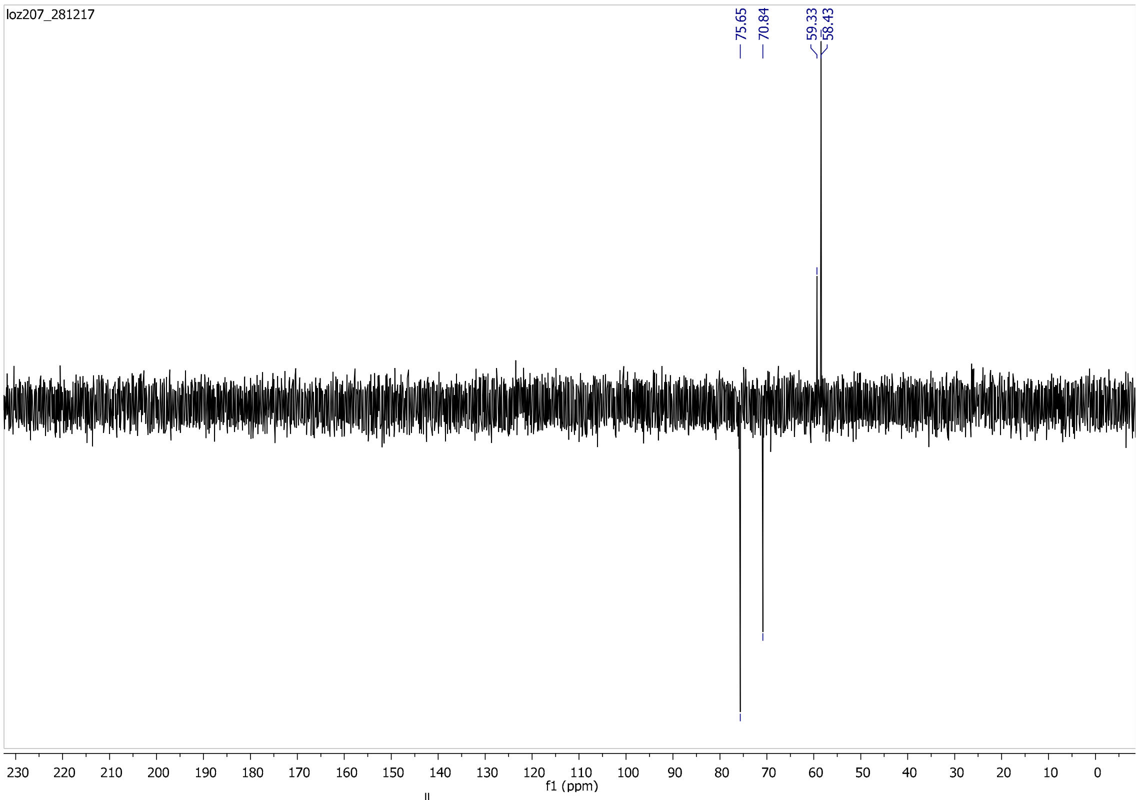
loz207_281217
75.65
70.84
59.33
58.43
230 220 210 200 190 180 170 160 150 140 130 120 110 100 90 80 70 60 50 40 30 20 10 0
f1 (ppm)

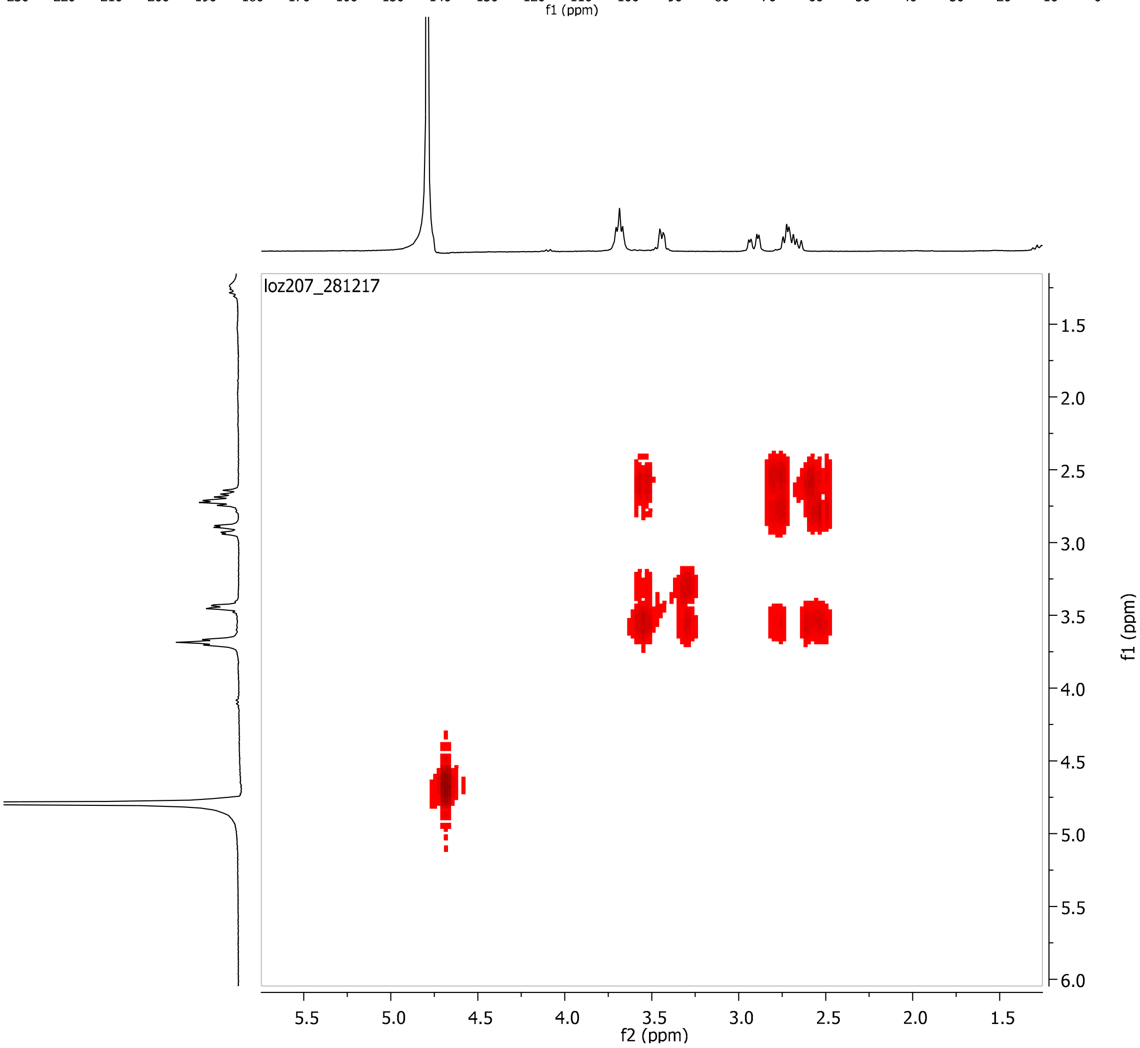
loz207_281217
5.5 5.0 4.5 4.0 3.5 3.0 2.5 2.0 1.5
f2 (ppm)
1.5 2.0 2.5 3.0 3.5 4.0 4.5 5.0 5.5 6.0
f1 (ppm)

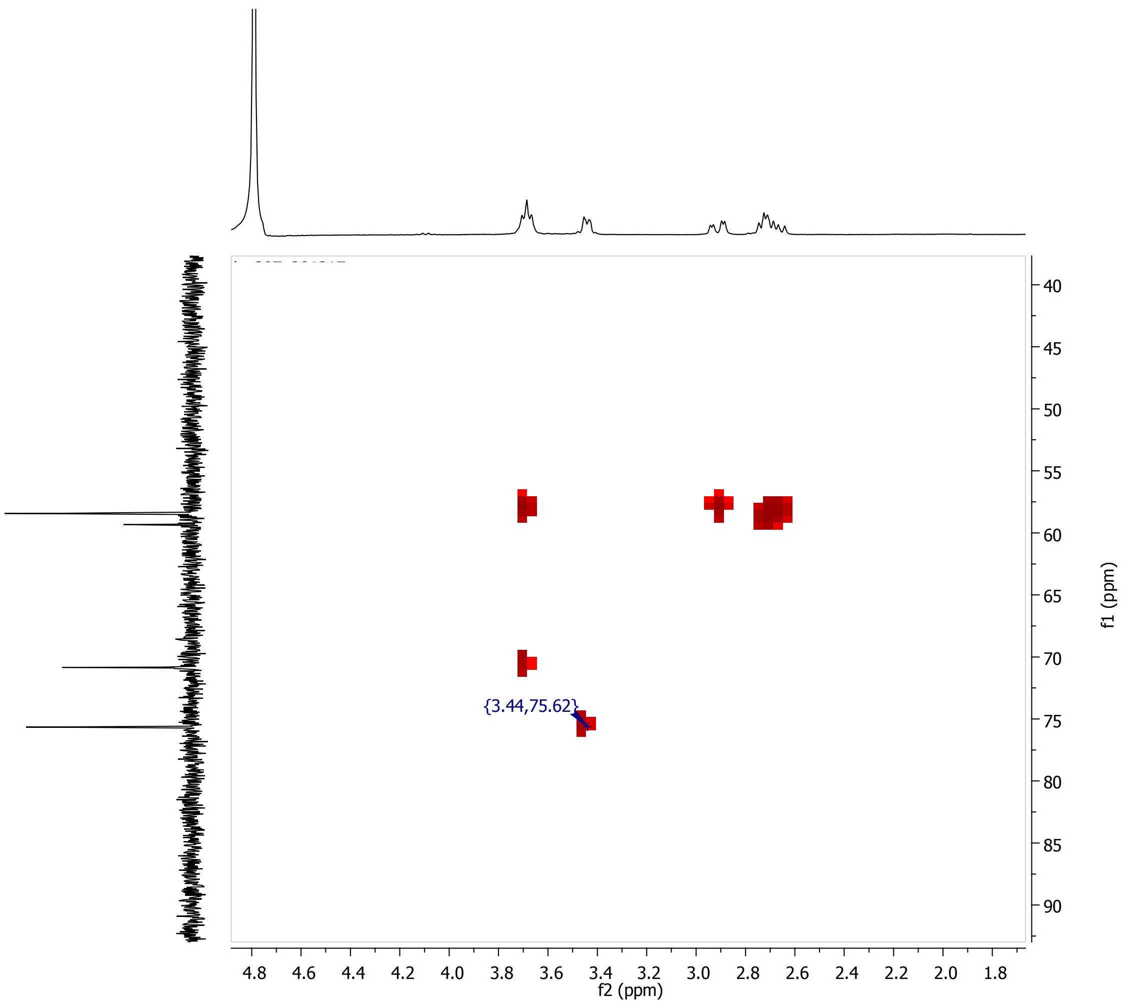

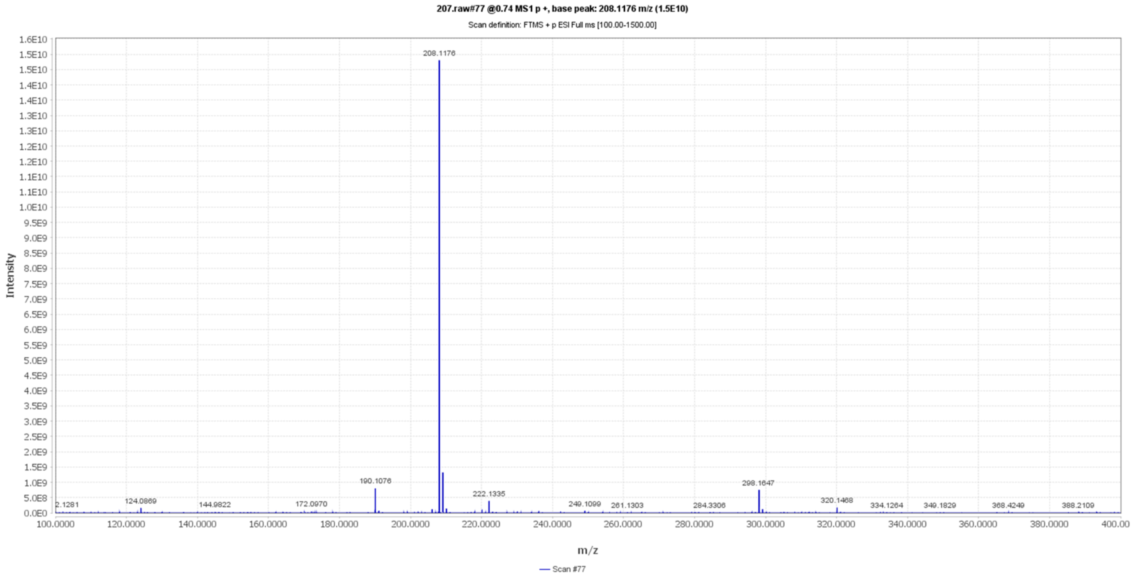

ESI HRMS: $[M+H]^+$ calcd for $C_8H_{18}NO_5$ 208.1180; found 208.1176

	Structure	*Yeast* α-Glucosidase	*Almond* β-Glucosidase
**DNJ**	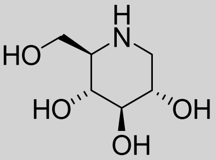	134.4±2.1	33.1±3.1
**26a**	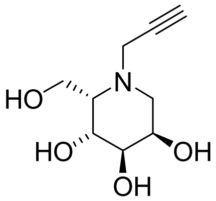	NI	1716±12.8
**26b**	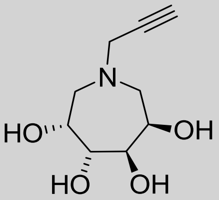	NI	NI
**27a**	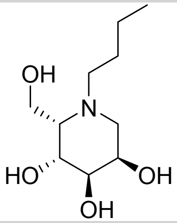	NI	109.7±9.3
**27b**	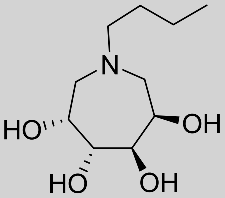	2031±17.1	184.6±2.6
**28a**	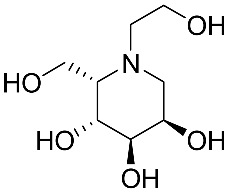	NI	NI
**28b**	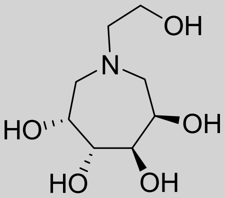	NI	NI
**29a**	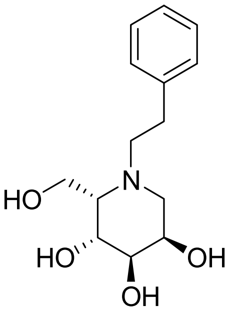	NI	NI

**29b**	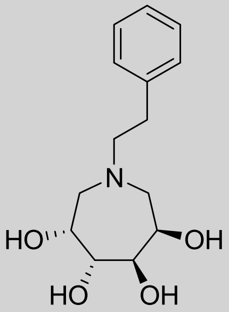	NI	NI
**39a**	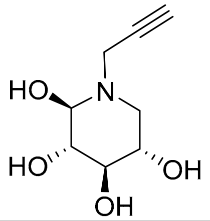	2527±82.2	635.7±8.5
**39b**	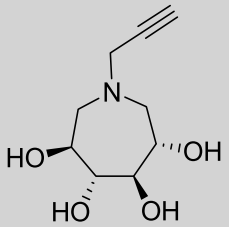	NI	3437±70.6
**40a**	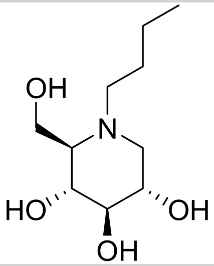	NI	172.8±1.7
**40b**	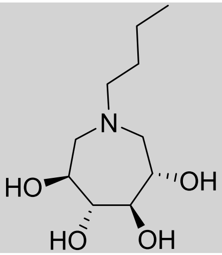	NI	80.0±4.9
**41a**	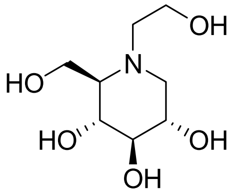	41.3±10.2	4.0±1.5
**41b**	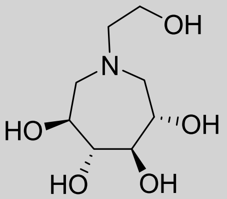	138.8±1.2	4.1±1.4

α-Glucosidase Yeast

26a

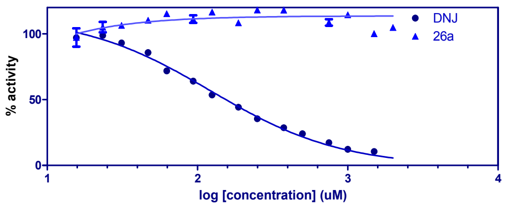

26b

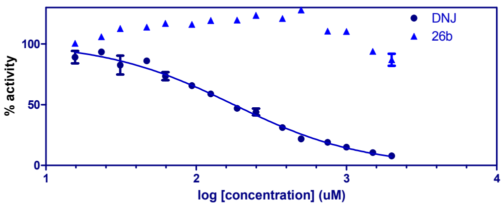

27a

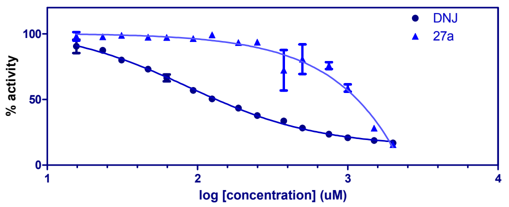

27b

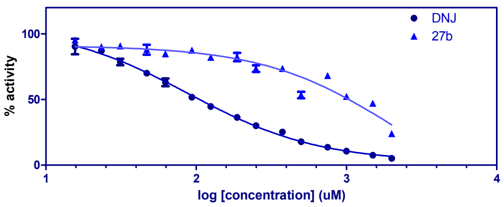

28a

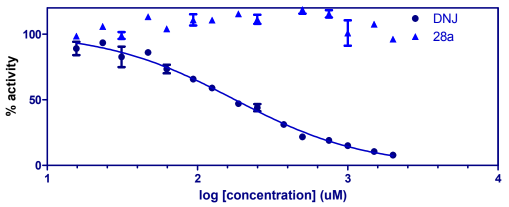

28b

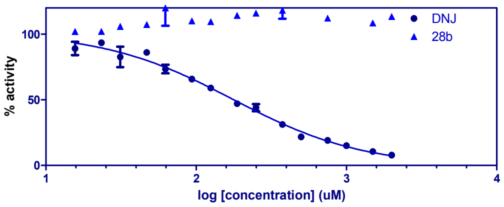

29a

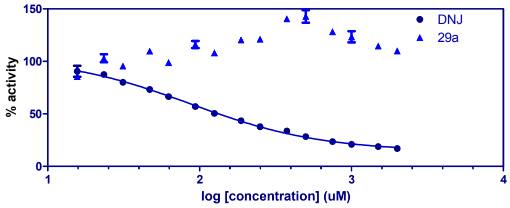

29b

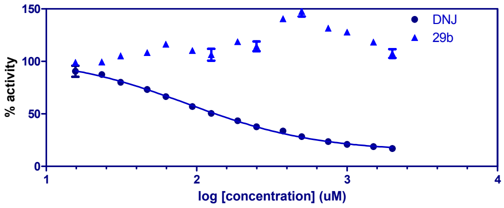

39a

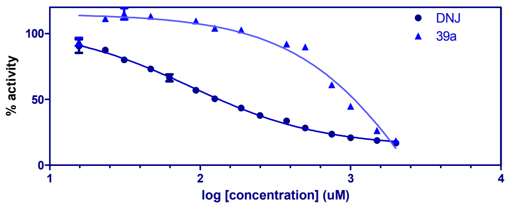

39b

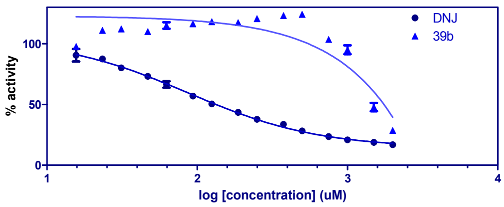

40a

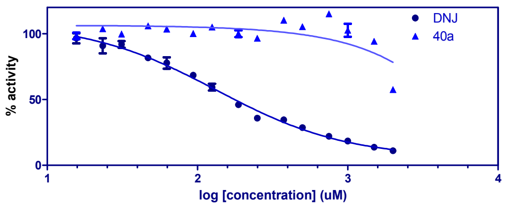

40b

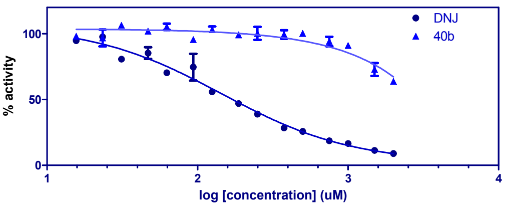

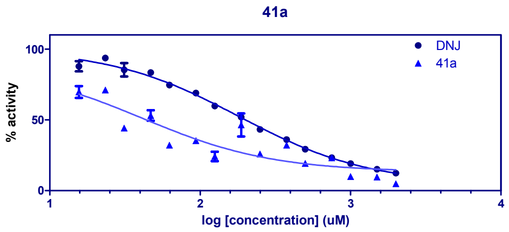
41a
DNJ
41a
100
50
0
% activity
1
2
3
4
log [concentration] (uM)

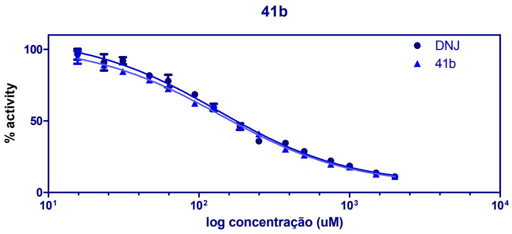
41b
DNJ
41b
100
50
0
% activity
10^1
10^2
10^3
10^4
log concentração (uM)

β-Glucosidase Almond

26a

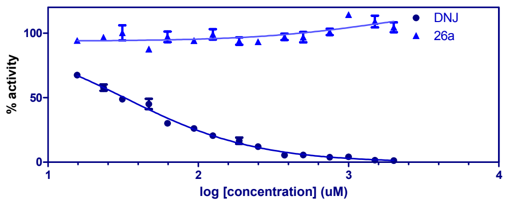

26b

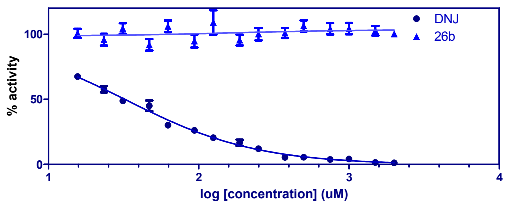

27a

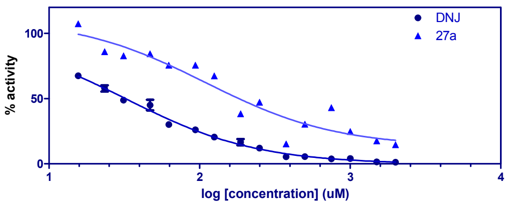

27b

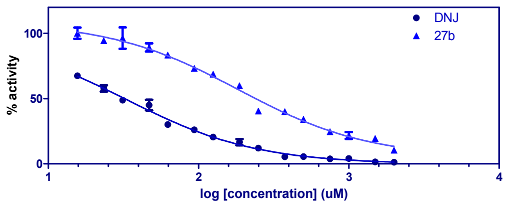

28a

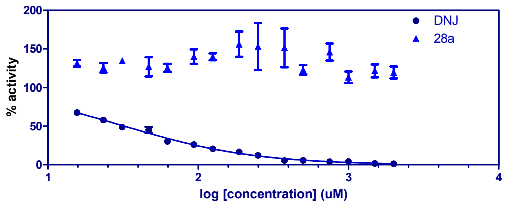

28b

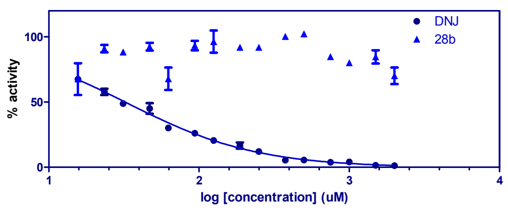

29a

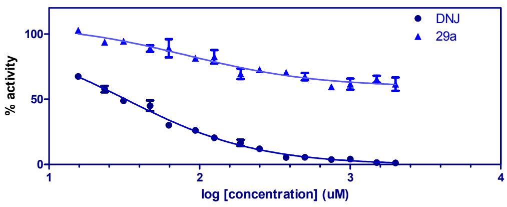

29b

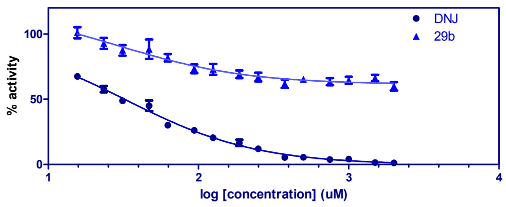

39a

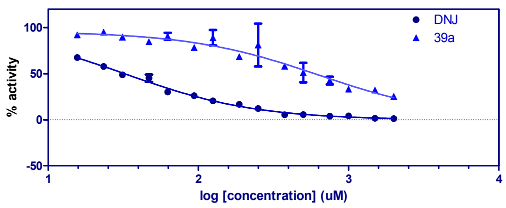

39b

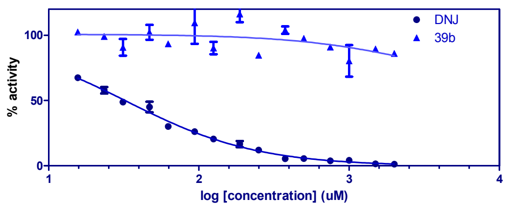

40a

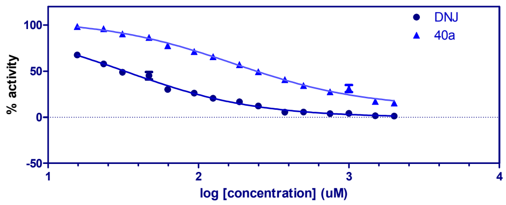

40b

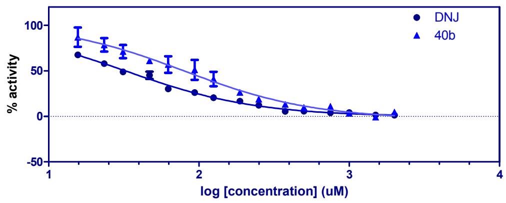

41a

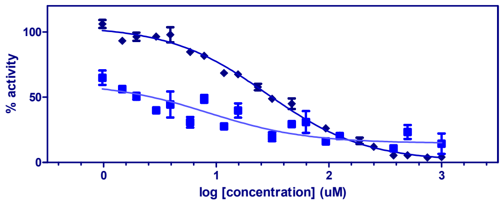

41b

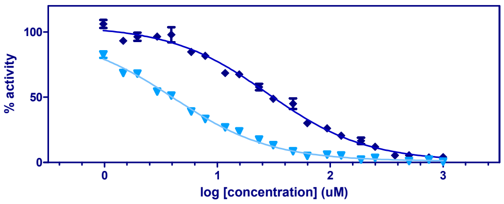

Supplement: Supplementary file 1 [file pharmaceuticals-12-00108-s001.pdf]
